# Population health outcomes in Nigeria compared with other west African countries, 1998–2019: a systematic analysis for the Global Burden of Disease Study

**DOI:** 10.1016/S0140-6736(21)02722-7

**Published:** 2022-03-19

**Authors:** Blake Angell, Olutobi Sanuade, Ifedayo M O Adetifa, Iruka N Okeke, Aishatu Lawal Adamu, Muktar H Aliyu, Emmanuel A Ameh, Fatima Kyari, Muktar A Gadanya, Diana A Mabayoje, Adesola Yinka-Ogunleye, Tolu Oni, Rabiu Ibrahim Jalo, Fatimah I Tsiga-Ahmed, Sarah L Dalglish, Seye Abimbola, Tim Colbourn, Obinna Onwujekwe, Eme Theodora Owoaje, Gambo Aliyu, Sani H Aliyu, Belinda Archibong, Alex Ezeh, Chikwe Ihekweazu, Zubairu Iliyasu, Stephen Obaro, Ebenezer Babatunde Obadare, Friday Okonofua, Muhammed Pate, Babatunde L Salako, Fatima H Zanna, Scott Glenn, Ally Walker, Maha Ezalarab, Mohsen Naghavi, Ibrahim Abubakar

**Affiliations:** a UCL Institute for Global Health, University College London, London, UK; b The George Institute for Global Health, University of New South Wales, Sydney, Sydney, NSW, Australia; c Center for Global Cardiovascular Health, Northwestern University Feinberg School of Medicine, Chicago, IL, USA; d Department of Infectious Diseases Epidemiology, London School of Hygiene & Tropical Medicine, London, UK; e Department of Epidemiology and Demography, Kenya Medical Research Institute-Wellcome Trust Research Programme, Kilifi, Kenya; f Department of Paediatrics and Child Health, College of Medicine, University of Lagos, Lagos, Nigeria; g Department of Pharmaceutical Microbiology, Faculty of Pharmacy, University of Ibadan, Ibadan, Nigeria; h Department of Community Medicine, Bayero University, Aminu Kano Teaching Hospital, Kano, Nigeria; i Vanderbilt Institute for Global Health, Vanderbilt University Medical Center, Nashville, TN, USA; j Division of Paediatric Surgery, National Hospital, Abuja, Nigeria; k College of Health Sciences, University of Abuja, Abuja, Nigeria; l University College London Hospitals NHS Foundation Trust, London, UK; m Nigeria Centre for Disease Control, Abuja, Nigeria; n MRC Epidemiology Unit, University of Cambridge, Cambridge, UK; o Research Initiative for Cities Health and Equity, School of Public Health and Family Medicine, University of Cape Town, Cape Town, South Africa; p School of Public Health, University of Sydney, Sydney, NSW, Australia; q Health Policy Research Group, University of Nigeria Enugu Campus, Enugu, Nigeria; r Department of Community Medicine, University of Ibadan College of Medicine, Ibadan, Nigeria; s National Agency for the Control of AIDS, Abuja, Nigeria; t Infectious Disease and Microbiology, Addenbrookes Hospital, Cambridge University Hospitals NHS Foundation Trust, Cambridge, UK; u Barnard College, New York, NY, USA; v Dornsife School of Public Health, Drexel University, Philadelphia, PA, USA; w Department of Pediatric Infectious Diseases, University of Nebraska Medical Center, Omaha, NE, USA; x Sociology Department, University of Kansas, Lawrence, KA, USA; y Centre of Excellence in Reproductive Health Innovation, University of Benin, Benin City, Edo State, Nigeria; z University of Medical Sciences, Ondo City, Nigeria; aa Health, Nutrition, and Population Global Practice and Global Financing Facility for Women, Children and Adolescents, World Bank, Washington, DC, USA; ab Harvard T H Chan School of Public Health, Cambridge, MA, USA; ac Nigeria Institute for Medical Research, Lagos, Nigeria; ad Institute for Health Metrics and Evaluation, University of Medicine Washington, Seattle, WA, USA

## Abstract

**Background:**

Population-level health and mortality data are crucial for evidence-informed policy but scarce in Nigeria. To fill this gap, we undertook a comprehensive assessment of the burden of disease in Nigeria and compared outcomes to other west African countries.

**Methods:**

In this systematic analysis, using data and results of the Global Burden of Diseases, Injuries, and Risk Factors Study 2019, we analysed patterns of mortality, years of life lost (YLLs), years lived with disability (YLDs), life expectancy, healthy life expectancy (HALE), and health system coverage for Nigeria and 15 other west African countries by gender in 1998 and 2019. Estimates of all-age and age-standardised disability-adjusted life-years for 369 diseases and injuries and 87 risk factors are presented for Nigeria. Health expenditure per person and gross domestic product were extracted from the World Bank repository.

**Findings:**

Between 1998 and 2019, life expectancy and HALE increased in Nigeria by 18% to 64·3 years (95% uncertainty interval [UI] 62·2–66·6), mortality reduced for all age groups for both male and female individuals, and health expenditure per person increased from the 11th to third highest in west Africa by 2018 (US$18·6 in 2001 to $83·75 in 2018). Nonetheless, relative outcomes remained poor; Nigeria ranked sixth in west Africa for age-standardised mortality, seventh for HALE, tenth for YLLs, 12th for health system coverage, and 14th for YLDs in 2019. Malaria (5176·3 YLLs per 100 000 people, 95% UI 2464·0–9591·1) and neonatal disorders (4818·8 YLLs per 100 000, 3865·9–6064·2) were the leading causes of YLLs in Nigeria in 2019. Nigeria had the fourth-highest under-five mortality rate for male individuals (2491·8 deaths per 100 000, 95% UI 1986·1–3140·1) and female individuals (2117·7 deaths per 100 000, 1756·7–2569·1), but among the lowest mortality for men older than 55 years. There was evidence of a growing non-communicable disease burden facing older Nigerians.

**Interpretation:**

Health outcomes remain poor in Nigeria despite higher expenditure since 2001. Better outcomes in countries with equivalent or lower health expenditure suggest health system strengthening and targeted intervention to address unsafe water sources, poor sanitation, malnutrition, and exposure to air pollution could substantially improve population health.

**Funding:**

The Bill & Melinda Gates Foundation.

## Introduction

West Africa is home to 16 countries with a combined population of 400 million people, 206 million of whom live in Nigeria, Africa's most populous nation.^1^ Nigeria faces numerous challenges that affect population health, many of which are also faced by other west African countries. Historically, health-care investment in Nigeria has been low by global standards, with high individual out-of-pocket costs restricting access to care.^2^ Nigeria has Africa's largest gross domestic product (GDP). However, economic growth has been variable and insufficient to keep pace with rapid population growth, with negative growth in per-capita incomes since 2015.^1^, ^3^, ^4^ Primary health care remains underdeveloped and other challenges such as extreme poverty, health worker shortages and absenteeism, so-called brain drain, and maldistribution of skilled health professionals compound these issues, further restricting access to care and reducing its quality for large parts of the population.^5^, ^6^, ^7^ High mobility between Nigeria and neighbouring countries combined with adverse climatic and environmental factors makes west Africa highly susceptible to rapid spread of infections, and there have been persistent disease outbreaks in the region over recent decades, including outbreaks of meningitis, Lassa fever, monkeypox, and Ebola (along with COVID-19).^8^, ^9^, ^10^, ^11^ Civil unrest around border communities in Nigeria influences health security in the wider region. West African countries also share similar health-care-seeking and health-risk behaviours that heighten the risk and burdens of non-communicable diseases (NCDs) across the region.^12^

**Research in context**

**Evidence before this study**

As indicated in *The Lancet* Nigeria Commission Report, no nationally representative primary or systematic review data were found to assess disease and mortality patterns across Nigeria. This poses challenges for policy makers looking to make evidence-informed policy and investment decisions. No studies were identified that provided a comprehensive comparison of the burden of diseases across west African nations either, which could be instructive for cross-country learning and diffusion of best practice across the region. The Global Burden of Diseases, Injuries, and Risk Factors Study 2019 estimated patterns of years of mortality, life expectancy, healthy life expectancy, years lived with disability, and disability-adjusted life years for 369 diseases and injuries and 87 risk factors for 204 countries and territories. However, no publications were found that analysed these data for Nigeria nor other west African countries.

**Added value of this study**

To our knowledge, this study is the first to provide a comprehensive assessment of the burden of diseases and risk factors and their trends over time in Nigeria and other west African countries. We provide age-standardised estimates for the number of disability-adjusted life years attributable to 369 conditions and 87 risk factors in Nigeria in 1998 and 2019 and, using standardised methods, compare key outcomes to 15 other west African countries. We show that despite notable gains in population health in Nigeria over this 21-year period, and some increased health expenditure per person, overall health outcomes remain poor, even compared with those of neighbouring countries with lower resources and health expenditure. Although infectious and neonatal conditions continue to be the primary drivers of morbidity and mortality in Nigeria and west Africa, there is evidence of a growing burden of non-communicable diseases. Our analysis of age-specific mortality in Nigeria highlights large discrepancies in the performance of the Nigerian health system, with under-five mortality remaining high by regional and global standards, whereas outcomes for men older than 55 years are among the best in the region. Despite limitations of data sources to parameterise models and the inability of models to account for all sources of bias, the international comparability of our data provides useful information to support evidence-informed policy making and have informed the work of The Lancet Nigeria Commission.

**Implications of all the available evidence**

There is substantial scope for Nigeria to improve population health outcomes. Health expenditure in Nigeria is low by global standards; however, comparisons with neighbouring countries with fewer resources suggest that important gains could be made through more efficient and equitable investment of existing resources, including shifting the burden of health-care financing away from out-of-pocket payments and by targeting preventive and primary-care interventions. Addressing key risk factors outside the health system, specifically, malnutrition, unsafe water sources and unsafe sanitation, and exposure to air pollution, would result in substantial improvements in population health and reduce the direct burden and demands on the health system. Overcoming the burden of maternal, neonatal, and child mortality must be the priority for the health system. Nigeria has among the lowest mortality in the region for men older than 55 years; however, the growing burden of non-communicable diseases represents a major threat to continued improvements in population health. Investments to address this growing burden must focus on preventive and primary health to improve population health and reduce the need for higher cost, later-stage care for these conditions that could redirect scarce resources away from the pressing need to reduce child and maternal mortality. Key policy implications of the findings include the need to identify feasible strategies to improve health system coverage in Nigeria, and to address the burden of maternal and child mortality and key risk-factors outside the health system. Examining policy settings in better-performing countries of the region in these areas might be instructive in guiding health-sector reform and multisectoral investments into prevention programmes in Nigeria.

Overcoming these challenges and improving population health in Nigeria requires targeted, evidence-based intervention to address the key drivers of ill health in the nation. Yet, robust population health and mortality data to inform the development and implementation of such interventions are scarce in Nigeria, posing further difficulties for policy makers.^13^ Further, although the health systems and underlying social and economic determinants of health vary across the region, many threats to population health in Nigeria are common across west Africa. Regional bodies such as the Economic Community of West African States including the West African Health Organization have demonstrated the potential for regional cooperation and cross-country learning for policy making in the region.^14^ Identifying areas of good and poor performance in countries with similar resources and facing similar challenges could help to identify practical, low-cost approaches to address the health problems facing Nigeria. To do so requires robust, comprehensive, and comparable data on the disease burden for the countries of the region, which, again, do not currently exist. To fill these gaps, we provide an analysis of the Global Burden of Diseases, Injuries, and Risk Factors Study (GBD) 2019. GBD provides policy-relevant annual estimates of mortality, morbidity, and attributable risk for 204 countries and territories, allowing international comparison and benchmarking over time. We used these estimates to examine historical health outcomes in Nigeria and compared these to other west African countries.

## Methods

### Overview

The data sources, strategies to ensure data quality and external validity, statistical modelling approaches, and metrics for GBD 2019 have been reported in detail.^15^, ^16^ Briefly, GBD 2019 lists 369 diseases and injuries and 87 risk factors for 204 countries and territories. This analysis presents estimates of health loss and mortality for all causes and risk factors for Nigeria and 15 countries in west Africa (Benin, Burkina Faso, Cape Verde, Cote D'Ivoire, The Gambia, Ghana, Guinea, Guinea-Bissau, Liberia, Mali, Mauritania, Niger, Senegal, Sierra Leone, and Togo) across 23 age groups; for male individuals, female individuals, and both sexes combined. Health losses related to specific causes were reported as estimates of the mortality, years of life lost (YLLs), years lived with disability (YLDs), and disability-adjusted life-years (DALYs) associated with conditions or risk factors for 1998 and 2019. Uncertainty intervals (UIs) for all estimates were derived from 1000 samples of each outcome.

### Data sources

GBD 2019 relied on a total of 86 249 data sources to estimate the global burden of disease and 30 652 data sources to estimate risk-attributable burden; these data sources included censuses, household surveys, civil registration and vital statistics, disease registries, health service use, disease notifications, and other sources obtained from the published literature, governments, and collaborators. This included 578 Nigeria-specific sources (appendix p 1 and available via the GBD Sources Tool).^17^ We obtained estimates of GDP and health expenditure for each country from the World Bank data repository.^4^

### Morbidity, mortality, and causes of death

Estimates for outcome measures of interest by age, sex, location, and year were modelled using standardised GBD tools. The Cause of Death Ensemble model developed for the GBD study and Bayesian meta-regression were used to generate cause estimates of mortality, incidence, and prevalence for all causes, countries, age groups, and sexes mapped to the GBD cause list. Adult and child mortality were modelled separately. As per established GBD study methods, YLLs were estimated as the product of cause-specific deaths within each age group and standard life expectancy at each age. Disability weights were measured for 220 health states covering 1160 disease and injury sequelae through two rounds of surveys across nine countries (including Tanzania as the only African nation) and more than 60 000 respondents in total. These weights were multiplied by the prevalence of sequelae to estimate YLDs, adjusting for disease severity, exclusivity, and comorbidity. All-cause and cause-specific DALYs were estimated for 369 conditions and attributed to 87 risk factors by cause as the sum of YLDs and YLLs for each year, age group, sex, and location. The leading 15 causes of age-standardised mortality, DALYs, and YLLs in Nigeria were compared to rates in the other nations. Life expectancy was calculated for each country as was healthy life expectancy (HALE), which summarises YLDs and YLLs in a single measure of average population health, by extending the life table to capture adjustments for non-fatal health outcomes.^15^, ^16^ Time trends were examined for the leading 25 causes of YLLs in Nigeria for the entire population and adults aged 20–54 years, because they constitute most of the working-age population and to better examine the burden of NCDs among adults.

### Risk factor attribution

The contribution of risk factors to country disease burdens was assessed in GBD 2019 using a comparative risk-assessment framework that includes risk–outcome pairs (derived from systematic reviews) in the analysis to quantify the impact of 87 risk factors (behavioural, environmental, occupational, and metabolic). Estimates of risk exposure levels and distributions were modelled on the basis of published studies, household surveys, censuses, and other sources. These risk exposures were modelled relative to a counterfactual level of theoretical minimum risk for each risk factor, to estimate population-attributable fractions and the attributable burden of mortality and morbidity for each age group, sex, country, and year. For combinations of risk factors presented, estimates accounted for the mediation of risk factors through other risk factors to correct for overestimation of the attributable burdens of individual factors. The attributable burden of deaths or YLLs for a risk–outcome pair was calculated as the total number of deaths or YLLs for that outcome, multiplied by the calculated fraction of cases in a population that were attributable to a specific exposure.

### Role of the funding source

The funders of this study had no role in study design, data collection, data analysis, data interpretation, writing of this manuscript, or decision to submit this for publication.

## Results

**Figure 1:**
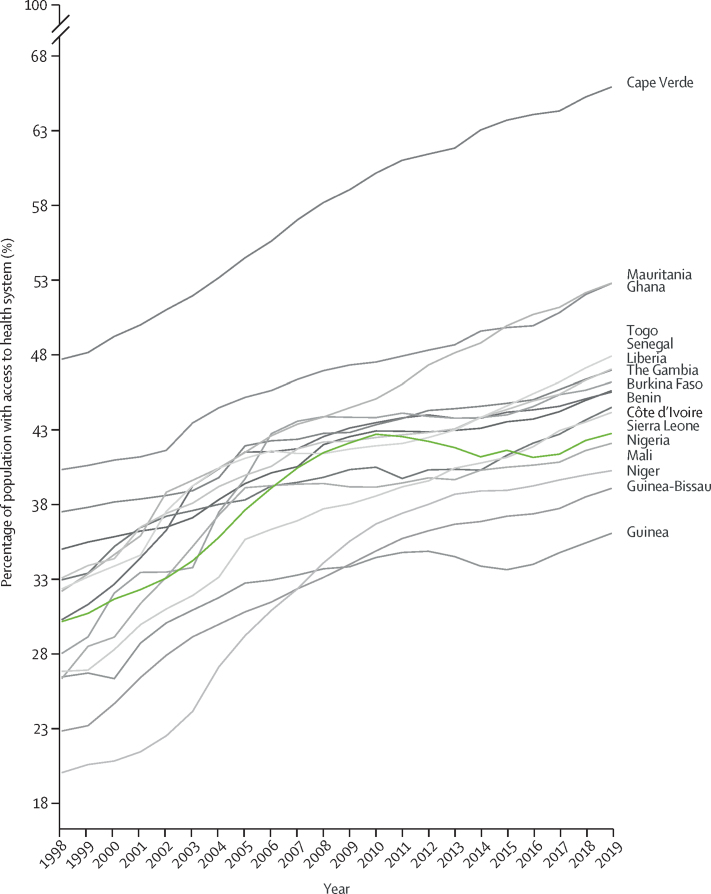
Health system coverage in west African nations, 1998–2019

Population health improved in Nigeria between 1998 and 2019. Life expectancy increased by 17·7% to 64·3 years (95% UI 62·2–66·6) and HALE increased by 17·9% to 56·0 years (53·0–58·9; table 1). Health expenditure per person increased by more than four times from US$18·6 in 2001 to $83·75 in 2018, which represented an increase from seventh-highest to third-highest ranked country in west Africa, and GDP per person increased from 11th highest to fourth highest in the region (table 2). Nonetheless, Nigeria had poorer outcomes than other countries in the region ranking; sixth in age-standardised death rates (ninth in 1998), tenth in YLLs (tenth in 1998), seventh in HALE (tenth in 1998), and 14th in YLD (13th in 1998; table 1). Nigeria had the highest proportion of health expenditure paid out of pocket in west Africa (77%), but the relatively high expenditure of the country did not translate to greater health system coverage. According to the GBD Universal Health Coverage Indicator, coverage was estimated at 43% in 2019 and has not kept up with other west African countries (ranked 12th in 2019, down from tenth in 1998; figure 1).

**Table 1:** Age-standardised death rates, YLLs, and YLDs for male and female sex combined in 1998 and 2019

	**Age-standardised death rate (per 100 000)**				**Age-standardised YLLs (per 100 000)**				**Age-standardised YLDs (per 100 000)**			
	1998		2019		1998		2019		1998		2019	
	Rate	Rank	Rate	Rank	Rate	Rank	Rate	Rank	Rate	Rank	Rate	Rank
Nigeria	1668 (1494–1842)	9	1143 (1007–1286)	6	70 701 (64 568– 76 789)	10	41 880 (36 535–48 133)	10	12 142 (8992– 15 840)	13	11 350 (8433–14 788)	14
Benin	1559 (1422–1705)	7	1185 (1003–1433)	8	60 185 (54 805– 65 719)	7	40 455 (32 729–50 529)	8	11 382 (8451– 14 888)	7	10 788 (8022–13 991)	4
Burkina Faso	1872 (1733–2019)	13	1324 (1190–1489)	14	77 532 (70 842– 84 352)	11	47 700 (41 690–55 254)	13	12 287 (9 039– 16 216)	14	11 069 (8140–14 499)	9
Cape Verde	874 (770– 983)	1	805 (736–878)	1	29 580 (26 140– 33 181)	1	19 582 (17 334–22 276)	1	10 428 (7725– 13 488)	1	10 349 (7714–13 387)	1
Côte d'Ivoire	2030 (1810– 2285)	15	1217 (1079–1395)	10	80 211 (70 567– 91 937)	12	40 597 (35 282–46 826)	9	12 355 (9226– 16 080)	16	11 251 (8348–14 669)	11
The Gambia	1402 (1241– 1579)	3	1172 (1029–1340)	7	49 178 (42 889– 55 778)	3	33 596 (28 952–39 083)	4	11 565 (8553– 15 150)	9	11 407 (8556–14 828)	15
Ghana	1464 (1319–1622)	5	1139 (1029–1289)	5	52 330 (47 463– 57 647)	5	35 124 (30 073–40 442)	5	11 093 (8219– 14 414)	3	10 841 (8115–14 133)	5
Guinea	1690 (1570–1829)	10	1373 (1154–1622)	15	69 668 (63 958– 75 899)	9	48 152 (39 249–58 528)	15	11 132 (8205– 14 623)	4	11 012 (8124–14 267)	8
Guinea-Bissau	2135 (1915–2356)	16	1485 (1256–1764)	16	84 681 (75 346– 93 957)	15	48 183 (40 139–58 154)	16	11 651 (8589– 15 251)	11	11 279 (8328–14 728)	12
Liberia	1522 (1291–1794)	6	1122 (985–1322)	4	49 913 (41 676– 60 139)	4	36 173 (30 837–42 793)	6	11 310 (8391– 14 769)	6	12 342 (9157–16 066)	16
Mali	1844 (1732–1961)	12	1274 (1086–1531)	12	80 627 (75 243– 86 084)	13	47 894 (39 148–59 324)	14	12 021 (8819– 15 746)	12	10 986 (8128–14 374)	7
Mauritania	1305 (1171–1425)	2	898 (792–1064)	2	45 264 (40 674– 49 865)	2	26 174 (21 257–32 958)	2	10 543 (7835– 13 811)	2	10 430 (7787–13 522)	2
Niger	1826 (1697–1968)	11	1264 (1078–1515)	11	81 762 (74 917– 89 624)	14	45 885 (37 503–56 760)	11	11 263 (8305– 14 751)	5	10 696 (7953–13 969)	3
Senegal	1438 (1360–1534)	4	1021 (923–1180)	3	53 456 (50 200– 57 123)	6	30 752 (26 658–35 989)	3	11 417 (8452– 14 991)	8	10 956 (8206–14 189)	6
Sierra Leone	1945 (1801–2100)	14	1313 (1136–1557)	13	85 402 (78 620– 92 270)	16	47 387 (39 033–57 391)	12	12 319 (9074– 16 044)	15	11 330 (8342–14 734)	13
Togo	1627 (1459–1803)	8	1186 (1042–1379)	9	61 565 (54 471– 69 106)	8	38 353 (32 508–45 199)	7	11 611 (8615– 15 112)	10	11 090 (8268–14 411)	10

Generally, 1 indicates best rank and 16 indicates worst rank. Particularly, countries with the lowest values in death rates, YLLs, and YLDs are ranked 1. YLD=years lived with disability. YLL=years of life lost.

**Table 2:** Age-standardised life expectancy at birth and HALE at birth for both sexes combined in 1998 and 2019

	**Life expectancy at birth (years)**				**HALE at birth (years)**			
	1998		2019		1998		2019	
	Life expectancy	Rank	Life expectancy	Rank	HALE	Rank	HALE	Rank
Nigeria	54·6 (52·7–56·8)	9	64·3 (62·2–66·6)	7	47·5 (44·8–50·1)	10	56·0 (53·0–58·9)	7
Benin	57·2 (55·3–59)	7	64·5 (60·6–67·7)	9	50·1 (47·6–52·5)	7	56·6 (52·8–60·2)	9
Burkina Faso	51·6 (49·9–53·5)	11	61·7 (59·2–63·9)	3	45·0 (42·5–47·3)	11	54·2 (51·1–56·9)	4
Cape Verde	69·7 (67·9–71·6)	1	73·7 (72·2–75·1)	16	61·1 (58·3–63·7)	1	64·6 (61·8–67·2)	16
Côte d'Ivoire	50·9 (47·9–53·8)	14	64·3 (61·9–66·5)	7	44·6 (41·5–47·6)	13	56·2 (53·2–59·1)	8
The Gambia	61·5 (59·4–63·7)	3	66·7 (64·4–68·9)	13	53·8 (51·0–56·5)	3	58·3 (55·2–61·1)	13
Ghana	59·7 (57·8–61·6)	5	66·3 (64·2–68·3)	12	52·5 (49·9–54·9)	5	58·2 (55·4–60·8)	12
Guinea	54·3 (52·6–56·1)	10	61·2 (57·9–64·6)	2	47·8 (45·4–50·2)	9	53·9 (50·5–57·3)	2
Guinea-Bissau	49·8 (47·8–51·9)	15	61·0 (57·7–64·1)	1	43·9 (41·6–46·3)	15	53·6 (50·3–56·7)	1
Liberia	60·3 (57·1–63·4)	4	66·1 (63·5–68·5)	11	53·1 (49·9–56·1)	4	56·9 (53·5–59·9)	10
Mali	51·0 (49·5–52·6)	13	61·8 (57·9–65·2)	4	44·5 (42·2–46·7)	14	54·3 (50·3–57·8)	5
Mauritania	62·6 (60·8–64·5)	2	70·8 (67·8–73·1)	15	55·3 (52·6–57·8)	2	62·2 (58·7–65·3)	15
Niger	51·1 (48·9–53)	12	62·5 (58·5–65·9)	6	44·9 (42·6–47·3)	12	55·1 (51·2–58·5)	6
Senegal	59·7 (58·6–60·9)	5	68·5 (66·3–70·3)	14	52·3 (49·9–54·5)	6	59·9 (56·7–62·6)	14
Sierra Leone	49·8 (48·2–51·5)	15	61·8 (58·4–65)	4	43·3 (41·0–45·5)	16	54·1 (50·5–57·5)	3
Togo	56·6 (54·3–59)	8	65·0 (62·5–67·4)	10	49·6 (46·9–52·3)	8	57 (53·9–59·9)	11

Generally, 1 indicates best rank and 16 indicates worst rank. For life expectancy at birth and HALE at birth, 1 indicates the country with the highest values. HALE=healthy life expectancy.

**Figure 2:**
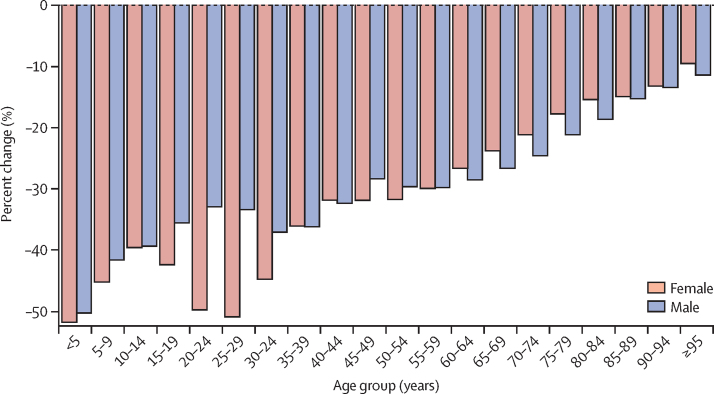
Change in mortality by age group in Nigeria, 1998–2019

**Figure 3:**
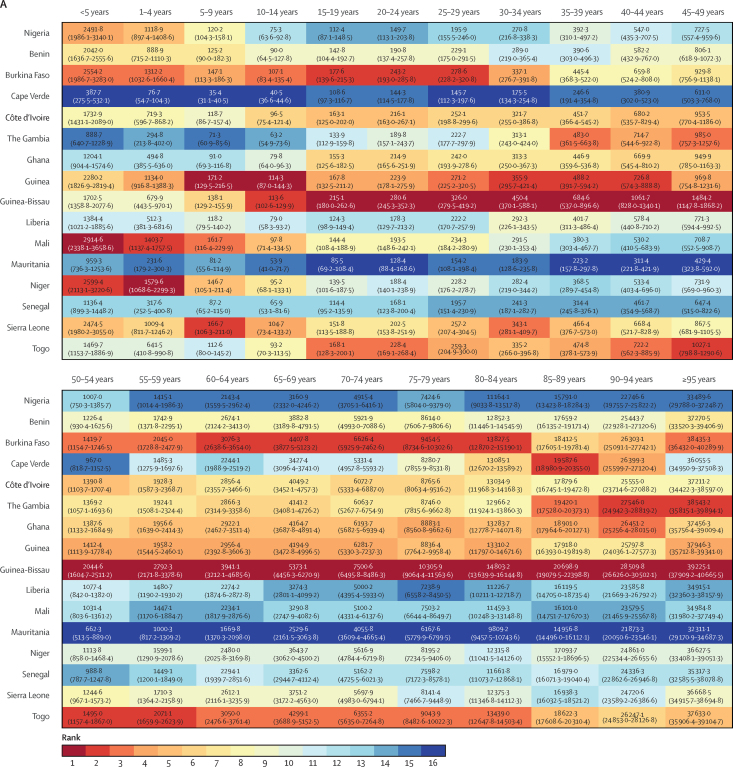

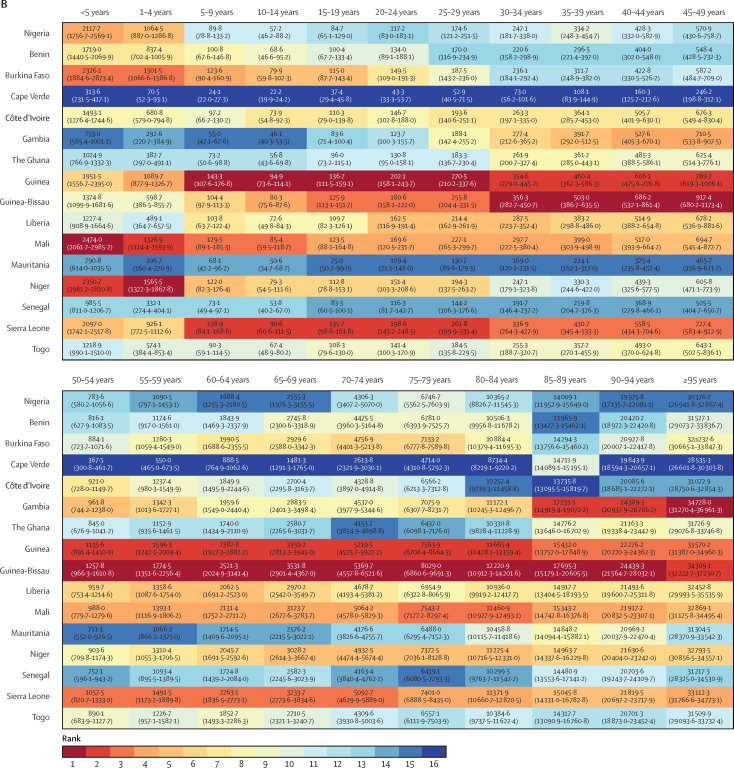
Rank of west African countries for age-specific mortality in 2019 Mortality in male (A) and female (B) individuals.

Mortality rates decreased for all ages in Nigeria between 1998 and 2019, with large gains for children younger than 5 years (more than 50% reductions for both boys and girls), those aged 5–9 years, and women aged 15–34 years, who had reductions in mortality of more than 40% (figure 2). Nonetheless, Nigeria had the fourth-worst under-five mortality in the region for male and female individuals in 2019, behind Mali, Niger, and Burkina Faso (figure 3), all poorer countries with lower health expenditure (table 2). Nigeria had among the lowest mortality for male individuals older than 55 years, ranked second-best across most older age groups behind Mauritania. Mortality rates in each age group were lower for female than male individuals. Compared with other west African nations, however, the relative rank of Nigeria was lower or equal in each age group for female mortality versus male mortality except for girls aged 5–9 years, for which Nigeria had the 11th-highest mortality compared with the eight-highest mortality for boys.

**Figure 4:**
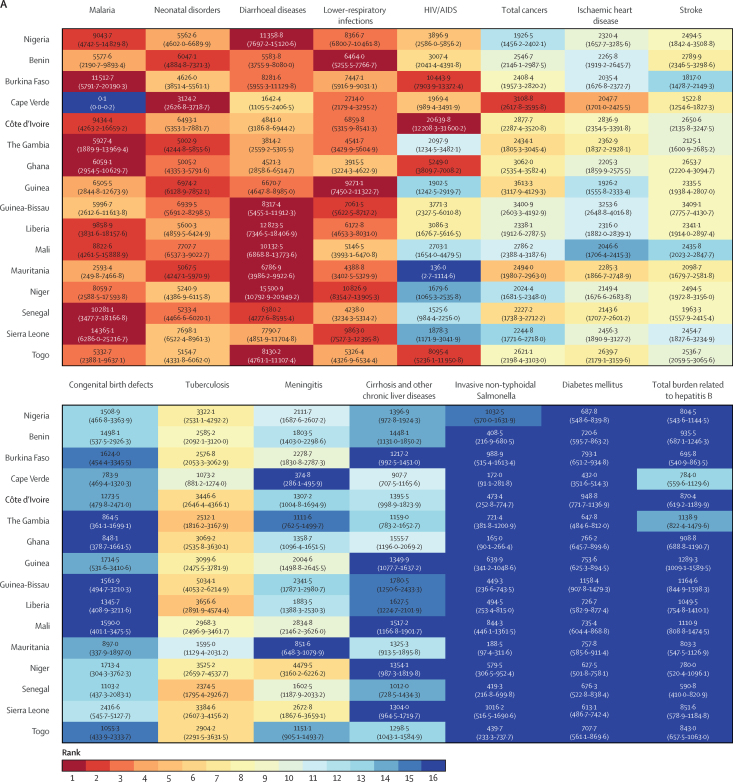

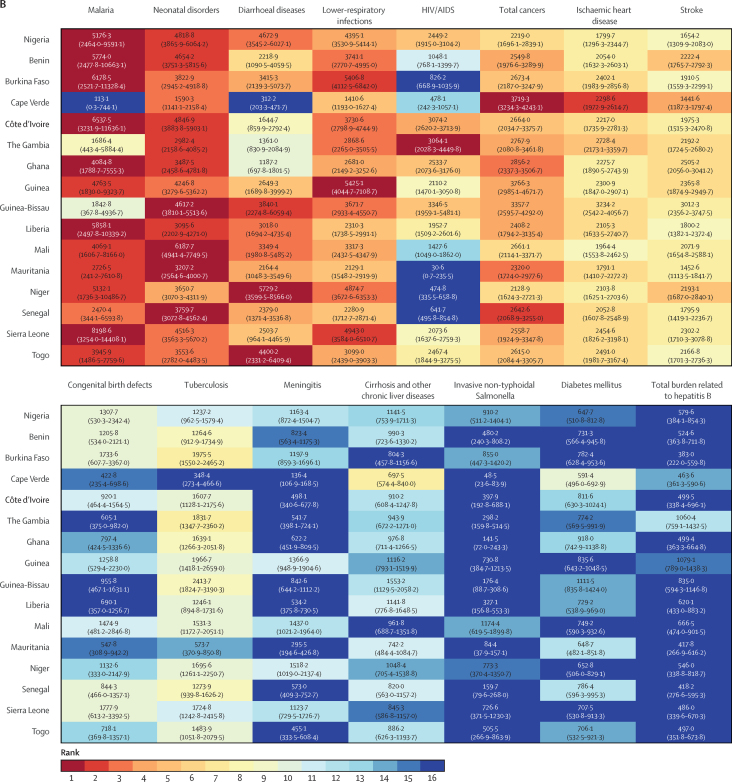
Rank of age-standardised years of life lost for the top 15 causes in Nigeria across west African countries for 1998 (A) and 2019 (B).

Infections and neonatal disorders were the dominant drivers of mortality in Nigeria, accounting for the top six causes of YLLs in 1998 and top five causes in 2019. In 1998, diarrhoeal diseases had the highest rate of YLLs in Nigeria, the 3rd-highest rate of YLLs in the region behind Niger and Liberia (figure 4). By 2019, despite a 59% reduction in the rate of YLLs, diarrhoeal diseases remained the third leading cause of YLLs in the country (figure 4; appendix p 36). Malaria was the second-leading cause of YLLs in Nigeria in 1998 and was among the top four causes of YLLs across all countries in the region, except for Cape Verde. By 2019, malaria was the leading cause of YLLs in Nigeria, Benin, Burkina Faso, Cote d'Ivoire, Ghana, Liberia, and Sierra Leone, and the second highest in Guinea (behind HIV and AIDS), Mali, Mauritania (behind neonatal disorders), Niger, and Togo (behind diarrhoeal diseases). Neonatal disorders, lower-respiratory infections, and HIV and AIDS were the other major drivers of YLLs in Nigeria and across most countries in the region in both 1998 and 2019. Although they remained the dominant driver of mortality in Nigeria, considerable gains were made in reducing the rate of age-standardised YLLs for infections and neonatal conditions with large reductions between 1998 and 2019 in the YLLs caused by tuberculosis (–63%, 95% UI –74 to –49), diarrhoeal diseases (–59%, –70 to –41), lower-respiratory infections (–48%, –59 to –32), malaria (–43%, –67 to –2), and HIV and AIDS (–37%, –50 to –19; appendix p 36). Across all countries, the relative contribution of cancers, stroke, and ischaemic heart disease to YLLs grew between 1998 and 2019 (figure 4). In Nigeria, there was an increase in the relative contribution of NCDs to mortality, and ischaemic heart disease, stroke, and congenital defects were in the top-ten causes of age-standardised YLLs in 2019, whereas the importance of cirrhosis (11th), chronic kidney disease (13th), and breast (18th) and prostate cancers (19th) has grown since 1998 (appendix p 36).

For Nigerians aged 20–54 years, HIV and AIDS was the leading contributor to YLLs, accounting for 4113·6 (95% UI 3085·0–5397·9) YLLs per 100 000 people (appendix p 37). As for the full population, significant gains were made in the rate of YLLs caused by infectious diseases over this period; however, YLLs caused by many NCDs grew in importance. Cirrhosis became the fifth largest contributor, ischaemic heart disease became the seventh largest contributor, and stroke became the eighth largest contributor to YLLs for this group in 2019, and breast cancer became the 12th leading cause of YLLs. Road injuries, interpersonal violence, and self-harm were major drivers of disease burden for people aged 20–54 years, with interpersonal violence accounting for 477·9 YLLs (95% UI 307·3–697·4), road injuries accounting for 415·7 YLLs (291·0–590·1), and self-harm accounting for 272·4 YLLs (175·4–418·9) per 100 000.

The pattern of age-standardised DALYs reflected that of YLLs in 1998 and 2019, with infections and neonatal disorders driving DALYs in Nigeria and all other west African countries (appendix pp 39–40). A complete table of DALYs attributable to all conditions is provided (appendix p 45). There was a preponderance of NCDs in the top fifteen causes of YLDs (accounting for 12 of the top 15 causes along with road injuries as the 15th leading cause) in Nigeria in 1998 and 2019. The rate of YLDs caused by diabetes (18th to 12th), other musculoskeletal conditions (17th to 11th), endocrine and metabolic conditions (15th to 13th), anxiety disorders (12th to tenth), and gynaecological diseases (ninth to eighth) all increased over the period (appendix p 41). The greatest progress in YLD reduction was seen for malaria, which dropped four places from 11th in 1998 to 15th in 2019, with considerable declines also observed for neglected tropical diseases, such as lymphatic filariasis, onchocerciasis, and intestinal nematodes (although not schistosomiasis). For every 100 000 people, children and young people contribute the least YLDs in Nigeria (appendix p 42); however, with the young age structure of the population, 88·9% of total YLDs occurred before 60 years of age. For those younger than 10 years, nutritional deficiencies and communicable diseases were the main causes of morbidity. From early adolescence (10–14 years) onwards, NCDs accounted for a larger share of YLDs initially through an increased burden of mental disorders and then sense-organ diseases (blindness and vision loss caused by eye diseases and age-related and other types of hearing loss) for older Nigerians. There was a marked reduction in age-standardised DALYs attributable to key risk factors in Nigeria between 1998 and 2019 (a full list of DALYs associated with 87 risk factors is provided; appendix pp 66–71). The five leading contributors to DALY loss in 2019 were risk factors affecting children (appendix p 43), including child growth failure, low birthweight, and short gestation. Other top-ten contributors to DALY loss were particulate-matter pollution, unsafe water sources, solid-fuel pollution, unsafe sanitation, and scarcity of handwashing facilities. The past decade has seen a substantial increase in the relative contribution of metabolic risk factors to the ill health of Nigerians (appendix p 44), which by 2019 accounted for 19% of deaths and almost 8% of total DALYs.

## Discussion

Estimated measures of population health improved in Nigeria between 1998 and 2019. Our estimates show that mortality decreased for all age groups in both male and female individuals, life expectancy increased by 10 years overall, HALE by 8 years, and the level of disability is estimated to have reduced across the population. Further, our estimates find that the nation increased health expenditures to the third highest in the region. Nonetheless, Nigeria had worse outcomes than many west African nations that showed greater improvements in health over the same period. Senegal with a lower GDP and health expenditure per person of US$59 and Mauritania with $54, for example, had lower mortality, higher HALE, and lower disability than Nigeria, with a health expenditure of $84. Although per-person health expenditure remains low in Nigeria by global standards and further investment is warranted, these patterns suggest substantial scope to increase the effectiveness of current amounts of expenditure by reallocating resources, redirecting investment to preventive and primary care, and improving the efficiency of the system.

Health system coverage remains low in Nigeria. Progress seen in some health outcomes is attributable to successful programmes, such as access to antiretroviral treatment, skilled birth attendants, immunisation, and improved diagnosis and treatment of malaria.^18^, ^19^, ^20^ There is room for substantial gains if the system can fully implement successful programmes across Nigeria. Cost-effective strategies to improve system coverage, for example by building on the existing community health workforce, may generate substantial gains in population health if this workforce is properly trained to deliver appropriate, cost-effective care.^21^, ^22^ At 77%, Nigeria had the greatest proportion of expenditure financed by out-of-pocket payments in the region, an inefficient and inequitable form of health-care financing and substantial barrier to health care for many people in the population.^23^, ^24^, ^25^ Widespread improvements in population health and system coverage will require a rebalancing of health-care financing in Nigeria away from out-of-pocket payments towards greater public investment into health. Although our estimates indicate that women experienced lower mortality than male individuals across all age groups and women of childbearing age in particular had large reductions in mortality over the study period, the performance of Nigeria for age-specific mortality estimates was consistently worse for female individuals than male individuals relative to other west African countries. Outcomes for men older than 55 years in Nigeria were particularly good relative to other countries; however, an increasing burden of NCDs threatens this good outcome and represents a major challenge to an already stretched health system. In keeping with *The Lancet* Nigeria Commission recommendations,^26^ effective intersectoral action to prevent NCDs would improve outcomes, reduce demands on the health system, and avoid shifting scarce resources towards late-stage care for these conditions at the expense of interventions to prevent and reduce neonatal, child, and maternal mortality and infections.^26^ Further investment needs to eliminate, rather than reinforce, existing inefficiencies and inequities in resource allocation across the system. Policy interventions will need to improve the effectiveness and resilience of the health system and specific recommendations have been outlined in *The Lancet* Nigeria Commission report to achieve better value and outcomes in Nigeria.^26^

Our analysis also suggests potential for large health gains through strategic investment outside the health system with nutrition-related, sanitation-related, and air pollution-related risk factors the most pressing drivers of disease burden in Nigeria and responsible for almost 90% of under-five mortality. The proportion of Nigerians with access to clean water (68·5%) and improved sanitation facilities (29%) is lower than many other west African nations and most nations globally, with some evidence of recent deterioration.^27^, ^28^ Without population-wide access to clean water and sanitary conditions, under-five mortality will remain stubbornly high in Nigeria. Ghana, for example, with similar GDP and health expenditure per person, has an under-five mortality rate less than half that of Nigeria and, despite Nigeria's higher baseline rate, there was a marked reduction in YLLs from diarrhoeal disease in Ghana (a decrease of 71·4%) relative to Nigeria (a decrease of 56·3%) over the study period. This probably reflects a variety of factors, including higher health system coverage in Ghana, higher rates of infant immunisation, but also a larger proportion of households with improved water sources (88·7% in Ghana).^28^ Malnutrition accounted for 54% of under-five mortality in Nigeria and addressing shortcomings in the food supply is fundamental to improving health. Recent improvements in access to fertilizer and support for farmers will assist malnutrition; however, there is an urgent need to build better agricultural distribution networks and tackle insecurity. Improving access to quality education for girls has been shown to be effective in improving early childhood nutrition.^29^ Air pollution is also having a major impact on illness and premature deaths in Nigeria, consistent with work done in Lagos that also highlighted the economic impact of air pollution on GDP.^30^ Multisectoral action to reduce exposure to these three risk factors (poor nutrition, poor sanitation, and air pollution) has the potential to drastically improve population health in Nigeria. Further, the combined contribution of injuries, self-harm, and interpersonal violence to the burden of ill health in Nigeria is substantial, particularly for adults aged 20–54 years. Known effective interventions to prevent these problems exist but have not yet been well implemented in Nigeria.^31^, ^32^, ^33^, ^34^, ^35^

We have provided comprehensive estimates for the burden of diseases in Nigeria, filling a gap in evidence that has severely curtailed the ability of policy makers to improve the health of Nigerians. Through standardised methods we have been able to investigate patterns of health loss between time periods and across comparable nations of west Africa. In doing so, we were able to highlight areas of good and poor performance and demonstrate that, even in resource-constrained systems, a judicious use of health expenditure might be more important than a simple increase in funding (as shown in Nigeria) to improving population health outcomes, contrary to some other suggestions.^36^, ^37^, ^38^, ^39^ There were several limitations to this work, however. Although uncertainties of the modelling process are incorporated into the estimates, because of the paucity of Nigeria-specific data these estimates rely on epidemiological projections based on population characteristics and disease-burden data in similar countries and regions, and thus might differ from the true burden if there are systemic differences between these. For example, data suggests a higher burden of typhoid fever relative to non-typhoidal salmonellosis, than presented in this analysis.^40^, ^41^ UIs do not take into account this measurement bias, selection bias caused by missing data, or model specification bias. Estimates for disability weights relied on respondents in other nations and assumed that DALYs consider premature deaths and YLDs to contribute equally to GBD estimates, irrespective of country of residence.^15^, ^16^ Despite our efforts, there is an urgent need for work to improve population health and mortality data in Nigeria, including subnational estimates. *The Lancet* Nigeria Commission Report outlines recommendations to achieve this improvement, most notably by ensuring that data collection, collation, and analysis are strengthened through a digital infrastructure linking data at the local, state, and federal level to the National Population Commission and the National Bureau of Statistics. We relied on World Bank estimates for health expenditure, which are derived from national estimates of WHO.

Between 1998 and 2019, healthy life expectancy and health expenditure increased in Nigeria; however, our estimates show disease-specific and overall health outcomes remain poor, even compared with poorer west African countries with much smaller health expenditure. This finding suggests important scope to improve population health through more efficient allocation and management of existing resources. This will require rebalancing the source of health-care financing from out-of-pocket costs to public investment and directing greater resources to preventive and primary care, including halting the increasing burden of NCDs facing the country. The contribution of environmental risk factors to the burden of disease in Nigeria highlights further opportunities for widespread gains if access to clean water, sanitation facilities, and food security is improved and exposure to air pollution reduced.

## Data sharing

All data used for this analysis are available via the GHDx portal (http://ghdx.healthdata.org/).

## Declaration of interests

GA is a current head of the National Agency for the Control of AIDS. CI and SA held leadership roles in the Nigerian Government during the period of writing of this paper. SA is editor in chief of *BMJ Global Health*. IA was a scientific and technical adviser to the Nigerian Government Presidential Task Force on COVID-19 and ZI is chair of the Nigerian national ethics committee. IA, FO, GA, SA, and IMOA are members of the Presidential Health Reform Committee. All other authors declare no competing interests.

## References

[1] UN (2019). World population prospects. https://population.un.org/wpp/.

[2] World Bank Current health expenditure (% of GDP). https://data.worldbank.org/indicator/SH.XPD.CHEX.GD.ZS?end=2018&start=2000.

[3] Vollset SE, Goren E, Yuan C-W (2020). Fertility, mortality, migration, and population scenarios for 195 countries and territories from 2017 to 2100: a forecasting analysis for the Global Burden of Disease Study. Lancet.

[4] World Bank World Bank Open Data. https://data.worldbank.org/.

[5] Kress DH, Su Y, Wang H (2016). Assessment of primary health care system performance in Nigeria: using the primary health care performance indicator conceptual framework. Health Syst Ref.

[6] Onwujekwe O, Agwu P, Orjiakor C (2019). Corruption in anglophone west Africa health systems: a systematic review of its different variants and the factors that sustain them. Health Pol Plan.

[7] Global Health Workforce Alliance Nigeria. https://www.who.int/workforcealliance/countries/nga/en/.

[8] Althaus CL, Low N, Musa EO, Shuaib F, Gsteiger S (2015). Ebola virus disease outbreak in Nigeria: transmission dynamics and rapid control. Epidemics.

[9] Yinka-Ogunleye A, Aruna O, Dalhat M (2019). Outbreak of human monkeypox in Nigeria in 2017–18: a clinical and epidemiological report. Lancet Infect Dis.

[10] Njidda AM, Oyebanji O, Obasanya J (2018). The Nigeria Centre for Disease Control. BMJ Global Health.

[11] Omoleke SA, Mohammed I, Saidu Y (2016). Ebola viral disease in west Africa: a threat to global health, economy and political stability. J Pub Health Africa.

[12] Afshin A, Sur PJ, Fay KA (2019). Health effects of dietary risks in 195 countries, 1990–2017: a systematic analysis for the Global Burden of Disease Study 2017. Lancet.

[13] Makinde OA, Odimegwu CO, Udoh MO (2020). Death registration in Nigeria: a systematic literature review of its performance and challenges. Glob Health Action.

[14] Uneke CJ, Sombie I, Johnson E, Uneke BI, Okolo S (2020). Promoting the use of evidence in health policymaking in the ECOWAS region: the development and contextualization of an evidence-based policymaking guidance. Glob Health.

[15] Vos T, Lim SS, Abbafati C (2020). Global burden of 369 diseases and injuries in 204 countries and territories, 1990–2019: a systematic analysis for the Global Burden of Disease Study 2019. Lancet.

[16] Murray CJ, Aravkin AY, Zheng P (2020). Global burden of 87 risk factors in 204 countries and territories, 1990–2019: a systematic analysis for the Global Burden of Disease Study 2019. Lancet.

[17] Institute for Health Metrics and Evaluation GHDx. http://ghdx.healthdata.org/.

[18] Murray CJ, Ortblad KF, Guinovart C (2014). Global, regional, and national incidence and mortality for HIV, tuberculosis, and malaria during 1990–2013: a systematic analysis for the Global Burden of Disease Study 2013. Lancet.

[19] National Malaria Elimination Program (2021). HMIS indicators. https://nmcp.gov.ng/hmis-indicators/.

[20] UNAIDS (2021). AIDS info. http://aidsinfo.unaids.org/.

[21] Joshi R, Alim M, Kengne AP (2014). Task shifting for non-communicable disease management in low and middle income countries–a systematic review. PloS one.

[22] Olaniran A, Madaj B, Bar-Zev S, van den Broek N (2019). The roles of community health workers who provide maternal and newborn health services: case studies from Africa and Asia. BMJ Glob Health.

[23] Kutzin J (2013). Health financing for universal coverage and health system performance: concepts and implications for policy. Bull World Health Organ.

[24] WHO (2014).

[25] Evans DB, Etienne C (2010). Health systems financing and the path to universal coverage. Bull World Health Org.

[26] Abubakar I, Dalglish SL, Angell B, et al. The *Lancet* Nigeria Commission: Investing in health and the future of the nation. *Lancet* (in press). 10.1016/S0140-6736(21)02488-0 PMC8943278 35303470

[27] WHO (2019).

[28] Agyepong IA, Sewankambo N, Binagwaho A (2017). The path to longer and healthier lives for all Africans by 2030: the Lancet Commission on the future of health in sub-Saharan Africa. Lancet.

[29] Shekar M, McDonald C, Okorosobo T (2015). Costed plan for scaling up nutrition in Nigeria. https://openknowledge.worldbank.org/handle/10986/21808?show=full.

[30] Croitoru L, Chang JC, Kelly A (2019). The cost of air pollution in Lagos. https://openknowledge.worldbank.org/handle/10986/33038?cid=env_tt_environment_en_ext.

[31] Matzopoulos R, Bowman B, Mathews S, Myers J (2010). Applying upstream interventions for interpersonal violence prevention: an uphill struggle in low-to middle-income contexts. Health Pol.

[32] WHO (2014).

[33] Forjuoh SN (2003). Traffic-related injury prevention interventions for low-income countries. J Inj Contr Saf Promot.

[34] Stevenson M, Yu J, Hendrie D (2008). Reducing the burden of road traffic injury: translating high-income country interventions to middle-income and low-income countries. Injur Prevent.

[35] Aggarwal S, Patton G, Berk M, Patel V (2021). Psychosocial interventions for self-harm in low-income and middle-income countries: systematic review and theory of change. Soc Psychiatry Psychiatr Epidemiol.

[36] Arthur E, Oaikhenan HE (2017). The effects of health expenditure on health outcomes in Sub-Saharan Africa (SSA). Afr Dev Rev.

[37] Kiross GT, Chojenta C, Barker D, Loxton D (2020). The effects of health expenditure on infant mortality in sub-Saharan Africa: evidence from panel data analysis. Health Econ Rev.

[38] Bein MA, Unlucan D, Olowu G, Kalifa W (2017). Healthcare spending and health outcomes: evidence from selected east African countries. Afr Health Sci.

[39] Rahman MM, Khanam R, Rahman M (2018). Health care expenditure and health outcome nexus: new evidence from the SAARC-ASEAN region. Glob Health.

[40] Akinyemi KO, Oyefolu AOB, Mutiu WB (2018). Typhoid fever: tracking the trend in Nigeria. Am J Trop Med.

[41] Popoola O, Kehinde A, Ogunleye V (2019). Bacteremia among febrile patients attending selected healthcare facilities in Ibadan, Nigeria. Clin Infect Dis.

