# Supplementary material for: Population health outcomes in Nigeria compared with other west African countries, 1998–2019: a systematic analysis for the Global Burden of Disease Study
Source: Lancet. 2022 Mar 19;399(10330):1117–29. doi: 10.1016/S0140-6736(21)02722-7 (PMC8943279; doi:10.1016/S0140-6736(21)02722-7)
Supplement: Supplementary appendix [file mmc1.pdf]

THE LANCET

Supplementary appendix

This appendix formed part of the original submission and has been peer reviewed. We post it as supplied by the authors.

Supplement to: Angell B, Sanuade O, Adetifa IMO, et al. Population health outcomes in Nigeria compared with other west African countries, 1998–2019: a systematic analysis for the Global Burden of Disease Study. *Lancet* 2022; published online March 15. https://doi.org/10.1016/S0140-6736(21)02722-7.

Supplementary Appendix

Table S1 – Sources used in GBD 2019 for Nigeria-specific estimates

For the Global Burden of Disease Study 2019 Data Input Sources Tool see http://ghdx.healthdata.org/.

- Aarhus University, Addiction Switzerland Research Institute, Alcohol Research Group, Public Health Institute, Centre for Addiction and Mental Health (Canada), Centre for Alcohol Policy Research, Turning Point Alcohol and Drug Centre (Australia), Kettil Bruun Society for Social and Epidemiological Research on Alcohol, University of North Dakota. Nigeria Gender, Alcohol and Culture: An International Study (GENACIS) 2003
- Abegunde KA, Owoaje ET. Health problems and associated risk factors in selected urban and rural elderly population groups of South-West Nigeria. Ann Afr Med. 2013; 12(2): 90-7
- Abu-Aisha H, Elamin S. Peritoneal dialysis in Africa. Perit Dial Int. 2010; 30(1): 23¬ñ8
- Abudu OO, Uguru V, Olude O. Contribution of congenital malformation to perinatal mortality in Lagos, Nigeria. Int J Gynaecol Obstet. 1988; 27(1): 63-7
- Adah MI, Rohwedder A, Olaleye OD, Durojaiye OA, Werchau H. Serotype of Nigerian rotavirus strains. Trop Med Int Health. 1997; 2(4): 363-70
- Adamu AN, Ekele BA, Ahmed Y, Mohammed BA, Isezuo SA, Abdullahpi AA. Pregnancy outcome in women with eclampsia at a tertiary centre in northern Nigeria. Afr J Med Med Sci. 2012; 41(2): 211-9
- Adamu AN, Okusanya BO, Tukur J, Ashimi AO, Oguntayo OA, Tunau KA, Ekele BA, Oladapo OT. Maternal near-miss and death among women with hypertensive disorders in pregnancy: a secondary analysis of the Nigeria Near-miss and Maternal Death Survey. BJOG. 2018
- Adebami OJ. Factors associated with the incidence of acute bilirubin encephalopathy in Nigerian population. J Pediatr Neurol. 2011; 9(3): 347-53
- Adedoyin D, Fagbule M a. &. Splenomegaly, malarial parasitaemia and anaemia in two Nigerian villages. Cent Afr J Med. 1992; 38(9): 371¬ñ5 as it appears in Malaria Atlas Project. Malaria Atlas Project Plasmodium Falciparum Parasite Rate Database. Oxford, United Kingdom: Malaria Atlas Project
- Adedoyin MA. The Pattern of Measles in Ilorin. West Afr J Med. 1990; 9(2): 103¬ñ7
- Adedoyin RA, Mbada CE, Balogun MO, Martins T, Adebayo RA, Akintomide A, Akinwusi PO. Prevalence and pattern of hypertension in a semiurban community in Nigeria. Eur J Cardiovasc Prev Rehabil. 2008; 15(6): 683-7
- Adegbembo AO, el-Nadeef MA. National survey of periodontal status and treatment need among Nigerians. Int Dent J. 1995; 45(3): 197-203
- Adegoke AA, Campbell M, Ogundeji MO, Lawoyin T, Thomson AM. Place of birth or place of death: An evaluation of 1139 maternal deaths in Nigeria. Midwifery. 2013; 29(11): e115-21
- Adegoke OA, Adedoyin RA, Balogun MO, Adebayo RA, Bisiriyu LA, Salawu AA. Prevalence of metabolic syndrome in a rural community in Nigeria. Metab Syndr Relat Disord. 2010; 8(1): 59¬ñ62
- Adekanle O, Ayodeji O, Olatunde L. Tetanus in a rural setting of South-Western Nigeria: a ten-year retrospective study. Libyan J Med. 2009; 4(2): 78-80
- Ademowo OG, Falusi AG, Mewoyeka OO. Prevalence of asymptomatic parasitaemia in an urban and rural community in south western Nigeria. Cent Afr J Med. 1995; 41(1): 18-21 as it appears in Malaria Atlas Project. Malaria Atlas Project Plasmodium Falciparum Parasite Rate Database. Oxford, United Kingdom: Malaria Atlas Project

- Adeoye IA, Onayade AA, Fatusi AO. Incidence, determinants and perinatal outcomes of near miss maternal morbidity in Ile-Ife Nigeria: a prospective case control study. BMC Pregnancy Childbirth. 2013; 93
- Adetoro OO. A 15-year study of illegally induced abortion mortality at Ilorin, Nigeria. Int J Gynaecol Obstet. 1989; 29(1): 65-72
- Adewemimo A, Kalter HD, Perin J, Koffi AK, Quinley J, Black RE. Direct estimates of cause-specific mortality fractions and rates of under-five deaths in the northern and southern regions of Nigeria by verbal autopsy interview. PLoS One. 2017; 12(5): e0178129
- Adeyoju AB, Olujohungbe AB, Morris J, Yardumian A, Bareford D, Akenova A, Akinyanju O, Cinkotai K, O'Reilly PH. Priapism in sickle-cell disease; incidence, risk factors and complications ¬ñ an international multicentre study. BJU Int. 2002; 90(9): 898¬ñ902
- Adinma ED. Maternal and perinatal outcome of eclampsia in tertiary health institution in Southeast Nigeria. J Matern Fetal Neonatal Med. 2013; 26(2): 211-4
- Adudu OP, Ogunrin OA, Adudu OG. Morbidity and mortality patterns among neurological patients in the intensive care unit of a tertiary health facility. Ann Afr Med. 2007; 6(4): 174-9
- African Cancer Registry Network (AFCRN), Calabar Cancer Registry. Five years Cancer Incidence in Calabar, Nigeria 2009-2013. Calabar, Nigeria: Calabar Cancer Registry, 2015
- Ahaneku GI, Osuji CU, Anisiuba BC, Ikeh VO, Oguejiofor OC, Ahaneku JE. Evaluation of blood pressure and indices of obesity in a typical rural community in eastern Nigeria. Ann Afr Med. 2011; 10(2): 120¬ñ6
- Airede AI. Birth asphyxia and hypoxic-ischaemic encephalopathy: incidence and severity. Ann Trop Paediatr. 1991; 11(4): 331-5
- Ajah LO, Ozonu NC, Ezeonu PO, Lawani LO, Obuna JA, Onwe EO. The Feto-Maternal Outcome of Preeclampsia with Severe Features and Eclampsia in Abakaliki, South-East Nigeria. J Clin Diagn Res. 2016; 10(9): QC18-QC21
- Ajenifuja KO, Adepiti CA, Ogunniyi SO. Post partum haemorrhage in a teaching hospital in Nigeria: a 5-year experience. Afr Health Sci. 2010; 10(1): 71-4
- Akenami FO, Koskiniemi M, Ekanem EE, Bolarin DM, Vaheri A. Seroprevalence and coprevalence of HIV and HBsAg in Nigerian children with/without protein energy malnutrition. Acta Trop. 1997; 64(34): 16774
- Akinremi, Taofeeq, Adewumi, Gbenga, Bratton, Michael, Olusegun, Olaniyan, Olusola, Moses, Liasu, Idris, ¬Ö Mattes, Robert. Afrobarometer Round 4: The Quality of Democracy and Governance in Nigeria, 2008. Ann Arbor, MI: Inter-university Consortium for Political and Social Research [distributor], 2012-08-06. https://doi.org/10.3886/ICPSR34009.v1
- Akogun OB, Akogun MK, Apake E, Kale OO. Rapid community identification, pain and distress associated with lymphoedema and adenolymphangitis due to lymphatic filariasis in resource-limited communities of North-eastern Nigeria. Addict Behav Rep. 2011; 120 Suppl 1: S62-68
- Akogun OB. Filariasis in Gongola State Nigeria. I: Clinical and parasitological studies in Mutum-Biyu District. Angew Parasitol. 1992; 33(3): 125-31 as it appears in London School of Hygiene and Tropical Medicine. Global Atlas of Helminth Infections - Lymphatic Filariasis. London, United Kingdom: London School of Hygiene and Tropical Medicine
- Akpala CO. Perinatal mortality in a northern Nigerian rural community. J R Soc Health. 1993; 113(3): 124-7
- Alabi O, Doctor HV, Jumare A, Sahabi N, Abdulwahab A, Findley SE, Abubakar SD. Health & demographic surveillance system profile: the Nahuche Health and Demographic Surveillance System, Northern Nigeria (Nahuche HDSS). Int J Epidemiol. 2014; 43(6): 1770-80

- Alasia DD, Emem-Chioma P, Wokoma FS. A single-center 7-year experience with end-stage renal disease care in Nigeria-a surrogate for the poor state of ESRD care in Nigeria and other sub-saharan african countries. Int J Nephrol. 2012; 2012: 639653
- Alasia DD, Emem-Chioma PC, Wokoma FS. Association of lead exposure, serum uric acid and parameters of renal function in Nigerian lead-exposed workers. Int J Occup Environ Med. 2010; 1(4): 182-90
- Alemika, Etannibi, Michael Bratton, and Peter Lewis. Afrobarometer: Round 1.5 Survey of Nigeria, 2001 . ICPSR04235-v1. Ann Arbor, MI:Bibliographic Citation: Inter-university Consortium for Political and Social Research [distributor], 2005-11-22. http://doi.org/10.3886/ICPSR04235.v1
- Alhaji MA, Bello MA, Elechi HA, Akuhwa RT, Bukar FL, Ibrahim HA. A review of neonatal tetanus in University of Maiduguri Teaching Hospital, North-eastern Nigeria. Niger Med J. 2013; 54(6): 398-401
- Alikor CA, Emem-Chioma PC. EPIDEMIOLOGY OF DIABETES AND IMPAIRED FASTING GLUCOSE IN A RURAL COMMUNITY OF NIGERIAN NIGER DELTA REGION. Niger J Med. 2015; 24(2): 114¬ñ24
- Aliyu A, Dahiru T, Obiako RO, Amadu L, Biliaminu LB, Akase EI. PATTERN AND OUTCOME OF TETANUS IN A TERTIARY HEALTH FACILITY IN NORTH WEST NIGERIA. Ethiop Med J. 2016; 54(2): 69-75
- Allison AC, Charles LJ, McGregor IA. Erythrocyte Glucose-6-Phosphate Dehydrogenase Deficiency in West Africa. Nature. 1961; 190(4782): 1198-9
- Aminu M, Ahmad AA, Umoh JU. Rotavirus infection in four states in north-western Nigeria. Niger J Med. 2008; 17(3): 285¬ñ90
- Amodu OK, Adeyemo AA, Olumese PE, Ketiku O, Gbadegesin RA. Intraleucocyte malaria pigment in asymptomatic and uncomplicated malaria. East Afr Med J. 1997; 74(11): 714¬ñ6 as it appears in Malaria Atlas Project. Malaria Atlas Project Plasmodium Falciparum Parasite Rate Database. Oxford, United Kingdom: Malaria Atlas Project
- Amodu OK, Olumide AO, Uchendu OC, Amodu FA, Omotade OO. Differences in the malariometric indices of asymptomatic carriers in three communities in Ibadan, Nigeria. Adv Prev Med. 2014 as it appears in Malaria Atlas Project. Malaria Atlas Project Plasmodium Falciparum Parasite Rate Database. Oxford, United Kingdom: Malaria Atlas Project
- Andersson N, Omer K, Caldwell D, Dambam MM, Maikudi AY, Effiong B, Ikpi E, Udofia E, Khan A, Ansari U, Ansari N, Hamel C. Male responsibility and maternal morbidity: a cross-sectional study in two Nigerian states. BMC Health Serv Res. 2011; S7
- Andy JJ, Peters EJ, Ekrikpo UE, Akpan NA, Unadike BC, Ekott JU. Prevalence and correlates of hypertension among the Ibibio/Annangs, Efiks and Obolos: a cross sectional community survey in rural South-South Nigeria. Ethn Dis. 2012; 22(3): 335¬ñ9
- Anejo-Okopi JA, Okojokwu JO, Ebonyi AO, Ejeliogu EU, Isa SE, Audu O, Akpakpan EE, Nwachukwu EE, Ifokwe CK, Ali M, Lar P, Oguche S. Molecular characterization of cryptosporidium in children aged 0- 5 years with diarrhea in Jos, Nigeria. Pan Afr Med J. 2016; 25: 253
- Angastiniotis M, Modell B, Englezos P, Boulyjenkov V, World Health Organization (WHO). Prevention and control of haemoglobinopathies. Bull World Health Organ. 1995; 73(3): 375-86
- Animasahun BA, Gbelee OH, Ogunlana AT, Njokanma OF, Odusanya O. Profile and outcome of patients with post-neonatal tetanus in a tertiary centre in south west Nigeria: any remarkable reduction in the scourge?. Pan Afr Med J. 2015; 21: 254

- Annual Report for Ikaram, Nigeria, Millenium Village. Year 1: February 2006 - February 2007 as it appears in Malaria Atlas Project. Malaria Atlas Project Plasmodium Falciparum Parasite Rate Database. Oxford, United Kingdom: Malaria Atlas Project
- Annual Report for Pampaida, Nigeria, Millennium Village. Year 1: February 2006 - February 2007 as it appears in Malaria Atlas Project. Malaria Atlas Project Plasmodium Falciparum Parasite Rate Database. Oxford, United Kingdom: Malaria Atlas Project
- Anosike JC. The status of human filariasis in north-western zone of Bauchi State, Nigeria. Appl Parasitol. 1994; 35(2): 133-40
- Antia-Obong OE, Ekanem EE, Udo JJ, Utsalo SJ. Septicaemia among neonates with tetanus. J Trop Pediatr. 1992; 38(4): 173-5
- Antony KK. Pattern of cardiac failure in Northern Savanna Nigeria. Trop Geogr Med. 1980; 32(2): 118-25
- Anumudu C, Afolami M, Igwe C, Nwagwu M, Keshinro O. Nutritional anaemia and malaria in pre-school and school age children. Ann Afr Med. 2008; 7(1): 11 as it appears in Malaria Atlas Project. Malaria Atlas Project Plasmodium Falciparum Parasite Rate Database. Oxford, United Kingdom: Malaria Atlas Project
- Anumudu CI, Adepoju A, Adediran M, Adeoye O, Kassim A, Oyewole I, Nwuba RI. Malaria prevalence and treatment seeking behaviour of young Nigerian adults. Ann Afr Med. 2007; 5(2): 82-8 as it appears in Malaria Atlas Project. Malaria Atlas Project Plasmodium Falciparum Parasite Rate Database. Oxford, United Kingdom: Malaria Atlas Project
- Aribodor D, Njoku O, Eneanya C, Onyali I. Studies on prevalence of malaria and management practices of the Azia community, Ihiala L.G.A., Anambra State, south-east Nigeria. Niger J Parasitol. 2007; 24(1): 33-38 as it appears in Malaria Atlas Project. Malaria Atlas Project Plasmodium Falciparum Parasite Rate Database. Oxford, United Kingdom: Malaria Atlas Project
- Arinola O. Complement factors and circulating immune complexes in children with urinary schistosomiasis and asymptomatic malaria. Afr J Med Med Sci. 2005; 34(1): 9-13 as it appears in Malaria Atlas Project. Malaria Atlas Project Plasmodium Falciparum Parasite Rate Database. Oxford, United Kingdom: Malaria Atlas Project
- Arinze AUH, Onyebuchi NV, Isreal J. Genital chlamydia trachomatis infection among female undergraduate students of University of Port Harcourt, Nigeria. Niger Med J. 2014; 55(1): 9¬ñ13
- Arogundade FA, Bello IS, Kuteyi EA, Akinsola A. Patterns of presentation and mortality in tetanus: a 10-year retrospective review. Niger Postgrad Med J. 2004; 11(3): 198-202
- Arogundade FA, Sanusi AA, Hassan MO, Akinsola A. The pattern, clinical characteristics and outcome of ESRD in Ile-Ife, Nigeria: is there a change in trend?. Afr Health Sci. 2011; 11(4): 594-601
- Asekun-Olarinmoye EO, Lawoyin TO, Onadeko MO. Risk factors for neonatal tetanus in Ibadan, Nigeria. Eur J Pediatr. 2003; 162(7-8): 526-7
- Ashaye A, Ashaolu O, Komolafe O, Ajayi BG, Olawoye O, Olusanya B, Adeoti C. Prevalence and types of glaucoma among an indigenous African population in southwestern Nigeria. Invest Ophthalmol Vis Sci. 2013; 54(12): 7410-6
- Auld AF, Agolory SG, Shiraishi RW, Wabwire-Mangen F, Kwesigabo G, Mulenga M, Hachizovu S, Asadu E, Tuho MZ, Ettiegne-Traore V, Mbofana F, Okello V, Azih C, Denison JA, Tsui S, Koole O, Kamiru H, Nuwagaba-Biribonwoha H, Alfredo C, Jobarteh K, Odafe S, Onotu D, Ekra KA, Kouakou JS, Ehrenkranz P, Bicego G, Torpey K, Mukadi YD, van Praag E, Menten J, Mastro T, Dukes Hamilton C, Swaminathan M, Dokubo EK, Baughman AL, Spira T, Colebunders R,

Bangsberg D, Marlink R, Zee A, Kaplan J, Ellerbrock TV, Centers for Disease Control and Prevention (CDC). Antiretroviral therapy enrollment characteristics and outcomes among HIV-infected adolescents and young adults compared with older adults--seven African countries, 2004-2013. MMWR Morb Mortal Wkly Rep. 2014; 63(47): 1097¬ñ103

- Ayanbimpe GM, Taghir H, Diya A, Wapwera S. Tinea capitis among primary school children in some parts of central Nigeria. Mycoses. 2008; 51(4): 336-40
- Ayoola EA. Antibody to hepatitis A virus in healthy Nigerians. J Natl Med Assoc. 1982; 74(5): 465-8
- Babalola OJ, Ibrahim IN, Kusfa IU, Gidado S, Nguku P, Olayinka A, Abubakar A. Measles outbreak investigation in an urban slum of Kaduna Metropolis, Kaduna State, Nigeria, March 2015. Pan Afr Med J. 2019; 32: 150
- Bada AS, Olatunji PO, Adewuyi JO, Iseniyi JO, Onile BA. Hepatitis B surface antigenaemia in Ilorin, Kwara State, Nigeria. Cent Afr J Med. 1996; 42(5): 139-41
- Bakare TI, Sowande OA, Adejuyigbe OO, Chinda JY, Usang UE. Epidemiology of external birth defects in neonates in Southwestern Nigeria. Afr J Paediatr Surg. 2009; 6(1): 28-30
- Bamgboye EA, Familusi JB. Mortality pattern at a children's emergency ward, University College Hospital, Ibadan, Nigeria. Afr J Med Med Sci. 1990; 19(2): 127-32
- Bashir M, Umar-Tsafe N, Getso K, Kaita IM, Nasidi A, Sani-Gwarzo N, Nguku P, Davis L, Brown MJ, Centers for Disease Control and Prevention (CDC). Assessment of blood lead levels among children aged MMWR Morb Mortal Wkly Rep. 2014; 63(15): 325-7
- Basic Support for Institutionalizing Child Survival (BASICS), Centers for Disease Control and Prevention (CDC), Johns Hopkins University, United States Agency for International Development (USAID). Nigeria Integrated Baseline Survey 1995
- Bellizzi S, Ali MM, Abalos E, Betran AP, Kapila J, Pileggi-Castro C, Vogel JP, Merialdi M. Are hypertensive disorders in pregnancy associated with congenital malformations in offspring? Evidence from the WHO Multicountry cross sectional survey on maternal and newborn health. BMC Pregnancy Childbirth. 2016; 16(1): 198
- Bello BT, Raji YR, Sanusi I, Braimoh RW, Amira OC, Mabayoje OM. Challenges of providing maintenance hemodialysis in a resource poor country: Experience from a single teaching hospital in Lagos, Southwest Nigeria. Hemodial Int. 2013; 17(3): 427¬ñ33
- Bhutani VK, Zipursky A, Blencowe H, Khanna R, Sgro M, Ebbesen F, Bell J, Mori R, Slusher TM, Fahmy N, Paul VK, Du L, Okolo AA, de Almeida MF, Olusanya BO, Kumar P, Cousens S, Lawn JE. Neonatal hyperbilirubinemia and Rhesus disease of the newborn: incidence and impairment estimates for 2010 at regional and global levels. Pediatr Res. 2013; 74(Suppl 1): 86-100
- Bratton, Michael, and Peter Lewis. Afrobarometer: Round I Survey of Nigeria, January-February 2000. ICPSR03440-v1. Ann Arbor, MI:Inter-university Consortium for Political and Social Research [distributor], 2003. http://doi.org/10.3886/ICPSR03440.v1
- Brieger W, Sesay H, Adesina H, Mosanya M, Ogunlade P, Ayodele J, Orisasona S. Urban malaria treatment behaviour in the context of low levels of malaria transmission in Lagos, Nigeria. Afr J Med Med Sci. 2000; 30(Suppl): 7-15 as it appears in Malaria Atlas Project. Malaria Atlas Project Plasmodium Falciparum Parasite Rate Database. Oxford, United Kingdom: Malaria Atlas Project
- Brinkhof MWG, Dabis F, Myer L, Bangsberg DR, Boulle A, Nash D, Schechter M, Laurent C, Keiser O, May M, Sprinz E, Egger M, Anglaret X, ART-LINC, IeDEA. Early loss of HIV-infected patients on potent antiretroviral therapy programmes in lower-income countries. Bull World Health Organ. 2008; 86(7): 559-67

- British Geological Survey, Natural Environment Research Council. World Mineral Production 2007-2011. Nottingham, United Kingdom: British Geological Survey, Natural Environment Research Council, 2013
- Brown I, Elliott P. SODIUM INTAKES AROUND THE WORLD [Internet]. Paris: World Health Organization;. 2006; 85 as it appears in Brown I, Elliott P. SODIUM INTAKES AROUND THE WORLD [Internet]. Paris: World Health Organization;. 2006; 85
- Butali A, Ezeaka C, Ekhaguere O, Weathers N, Ladd J, Fajolu I, Esezobor C, Makwe C, Odusanya B, Anorlu R, Adeyemo W, Iroha E, Egri-Okwaji M, Adejumo P, Oyeneyin L, Abiodun M, Badejoko B, Ryckman K. Characteristics and risk factors of preterm births in a tertiary center in Lagos, Nigeria. Pan Afr Med J. 2016; 24: 1
- Byass P,¬†Adedeji MD,¬†Mongdem JG,¬†Zwandor AC,¬†Brew-Graves SH,¬†Clements¬†CJ. Assessment and possible control of endemic measles in urban Nigeria. J Public Health Med. 1995; 17(2): 140-5
- CDC Foundation, Centers for Disease Control and Prevention (CDC), Federal Ministry of Health (Nigeria), Johns Hopkins Bloomberg School of Public Health, National Bureau of Statistics (Nigeria), World Health Organization (WHO). Nigeria Global Adult Tobacco Survey 2012
- Centers for Disease Control and Prevention (CDC), National Tuberculosis Control Programme (Nigeria), World Health Organization (WHO). Nigeria National Tuberculosis Prevalence Survey 2012
- Centers for Disease Control and Prevention (CDC). 2014-2016 Ebola Outbreak in West Africa. Atlanta, United States of America: Centers for Disease Control and Prevention (CDC), 2017
- Centers for Disease Control and Prevention (CDC). Guinea Worm Wrap No. 31. Atlanta, United States: Centers for Disease Control and Prevention (CDC), 1991
- Centers for Disease Control and Prevention (CDC). Progress Toward Global Eradication of Dracunculiasis, January 2008-June 2009. MMWR Morb Mortal Wkly Rep. 2008; 58(40): 1123-1125
- Central Bank of Nigeria, National Bureau of Statistics (Nigeria), Nigerian Communications Commission (NCC). Nigeria General Household Survey 2007. Abuja, Nigeria: National Bureau of Statistics (Nigeria)
- Central Bank of Nigeria, National Bureau of Statistics (Nigeria), Nigerian Communications Commission (NCC). Nigeria General Household Survey 2008
- Central Bank of Nigeria. Nigeria Annual Report and Statement of Accounts 2001. Abuja, Nigeria: Central Bank of Nigeria, 2002
- Central Bank of Nigeria. Nigeria Annual Report and Statement of Accounts 2002. Abuja, Nigeria: Central Bank of Nigeria, 2003
- Centre for Population and Reproductive Health (CPRH), University of Ibadan; Centre for Research, Evaluation Resources and Development (CRERD); Population and Reproductive Health Program (PRHP), Obafemi Awolowo University (OAU); Bayero University Kano (BUK); and The Bill & Melinda Gates Institute for Population and Reproductive Health at The Johns Hopkins Bloomberg School of Public Health. Performance Monitoring and Accountability 2020 (PMA2020) Survey round 1, PMA2014/Nigeria-R1 (Kaduna & Lagos). 2014. Nigeria and Baltimore, Maryland, USA
- Centre for Research, Evaluation Resources and Development (CRERD), Bayero University Kano (BUK), and The Bill & Melinda Gates Institute for Population and Reproductive Health at The Johns Hopkins Bloomberg School of Public Health. Performance Monitoring

and Accountability 2020 (PMA2020) Survey round 2, PMA2015/Nigeria-R2 (Kaduna & Lagos). 2015. Nigeria and Baltimore, Maryland, USA

- Centre for Research, Evaluation Resources and Development (CRERD), Bayero University Kano (BUK), and The Bill & Melinda Gates Institute for Population and Reproductive Health at The Johns Hopkins Bloomberg School of Public Health. Performance Monitoring and Accountability 2020 (PMA2020) Survey round 3, PMA2016/Nigeria-R3 (National). 2016. Nigeria and Baltimore, Maryland, USA
- Centre for Research, Evaluation Resources and Development (CRERD), Bayero University Kano (BUK), and The Bill & Melinda Gates Institute for Population and Reproductive Health at The Johns Hopkins Bloomberg School of Public Health. Performance Monitoring and Accountability 2020 (PMA2020) Survey round 4, PMA2017/Nigeria-R4 (National). 2017. Nigeria and Baltimore, Maryland, USA
- Centre for Research, Evaluation Resources and Development (CRERD), Bayero University Kano (BUK), and The Bill & Melinda Gates Institute for Population and Reproductive Health at The Johns Hopkins Bloomberg School of Public Health. Performance Monitoring and Accountability 2020 (PMA2020) Survey round 5, PMA2018/Nigeria-R5 (National). 2018. Nigeria and Baltimore, Maryland, USA
- Chapp-Jumbo EN. Neurologic infections in a Nigerian university teaching hospital. Afr Health Sci. 2006; 6(1): 55-8
- Characteristics of P. Falciparum Infections in Children in a Suburb of Ibadan, Nigeria as it appears in Malaria Atlas Project. Malaria Atlas Project Plasmodium Falciparum Parasite Rate Database. Oxford, United Kingdom: Malaria Atlas Project
- Chike-Obi U. Preterm delivery in Ilorin: multiple and teenage pregnancies as major aetiological factors. West Afr J Med. 1993; 12(4): 228¬ñ30
- Christiana O, Olajumoke M, Oyetunde S. Lymphatic filariasis and associated morbidities in rural communities of Ogun State, Southwestern Nigeria. Travel Med Infect Dis. 2014; 12.0(1): 95-101
- Chukudebelu WO, Esege N, Megafu U. Etiological factors in infertility in Enugu, Nigeria. Infertility. 1979; 2(2): 193-200
- Chukwubike OA, God'spower AE. A 10-year review of outcome of management of tetanus in adults at a Nigerian tertiary hospital. Ann Afr Med. 2009; 8(3): 168-72
- Chukwuma, Innocent, Akinremi, Taofeeq, Bratton, Michael, Gyimah-Boadi, E., and Mattes, Robert. Afrobarometer Round 3: The Quality of Democracy and Governance in Nigeria, 2005. Ann Arbor, MI: Inter-university Consortium for Political and Social Research [distributor], 2008-10-17. https://doi.org/10.3886/ICPSR22209.v1
- Chukwuma, Innocent, Akinremi, Taofeeq, Gyimah-Boadi, E., Bratton, Michael, Mattes, Robert, Logan, Carolyn, and Dulani, Boniface. Afrobarometer Round 5: The Quality of Democracy and Governance in Nigeria, 2012. Ann Arbor, MI: Inter-university Consortium for Political and Social Research [distributor], 2015-08-17. https://doi.org/10.3886/ICPSR35561.v1
- Community-Based Management of Acute Malnutrition (CMAM) Forum. Putting Child Kwarshiorkor on the Map. Community-Based Management of Acute Malnutrition (CMAM) Forum, 2016
- Cooper R, Rotimi C, Ataman S, McGee D, Osotimehin B, Kadiri S, Muna W, Kingue S, Fraser H, Forrester T, Bennett F, Wilks R. The prevalence of hypertension in seven populations of West African origin. Am J Public Health. 1997; 87(2): 160-8

- Cooper R, Rotimi C, Ataman S, McGee D, Osotimehin B, Kadiri S, Muna W, Kingue S, Fraser H, Forrester T, Bennett F, Wilks R. The prevalence of hypertension in seven populations of West African origin. Am J Public Health. 1997; 87(2): 160-8 as it appears in Cooper R, Rotimi C, Ataman S, McGee D, Osotimehin B, Kadiri S, Muna W, Kingue S, Fraser H, Forrester T, Bennett F, Wilks R. The prevalence of hypertension in seven populations of West African origin. Am J Public Health. 1997; 87(2): 160-8
- Cornelius AC, Onyegbule A, Onyema null, Uchenna ET, Duke OA. A five year review of ectopic pregnancy at Federal Medical Centre, Owerri, South East, Nigeria. Niger J Med. 2014; 23(3): 207¬ñ12
- Cystic Echinococcosis Endemicity Estimates identified through systematic review and personal communication, as provided by GBD 2015 expert. [Unpublished]
- Dada TO. Epilepsy in Lagos, Nigeria. Afr J Med Sci. 1970; 1( ): 161-184
- Dahiru T, Jibo A, Hassan AA, Mande AT. Prevalence of diabetes in a semi-urban community in Northern Nigeria. Niger J Med. 2008; 17(4): 414-6
- Dalhat MM, Isa AN, Nguku P, Nasir S-G, Urban K, Abdulaziz M, Dankoli RS, Nsubuga P, Poggensee G. Descriptive characterization of the 2010 cholera outbreak in Nigeria. BMC Public Health. 2014; 14(1): 1167
- Dalhatu I, Onotu D, Odafe S, Abiri O, Debem H, Agolory S, Shiraishi RW, Auld AF, Swaminathan M, Dokubo K, Ngige E, Asadu C, Abatta E, Ellerbrock TV. Outcomes of Nigeria's HIV/AIDS Treatment Program for Patients Initiated on Antiretroviral Treatment between 2004-2012. PLoS One. 2016; 11(11): e0165528
- Damasceno A, Mayosi BM, Sani M, Ogah OS, Mondo C, Ojji D, Dzudie A, Kouam CK, Suliman A, Schrueder N, Yonga G, Ba SA, Maru F, Alemayehu B, Edwards C, Davison BA, Cotter G, Sliwa K. The causes, treatment, and outcome of acute heart failure in 1006 Africans from 9 countries. Arch Intern Med. 2012; 172(18): 1386-94
- Danesi MA, Oni K. Features of partial epilepsy in Nigerians: a 3 year clinical and electroencephalographic study of 282 cases seen at the Lagos University Teaching Hospital. Afr J Neurol Sci. 1983; 2( ): 1-6
- Danesi MA. Acquired aetiological factors in Nigerian epileptics (an investigation of 378 patients). Trop Geogr Med. 1983; 35(3): 293-297
- Daniel OJ, Adejumo OA, Adejumo EN, Owolabi RS, Braimoh RW. Prevalence of hypertension among urban slum dwellers in Lagos, Nigeria. J Urban Health. 2013; 90(6): 1016¬ñ25
- Das SC, Isichei UP, Egbuta JO, Banwo AI. Cations and anions in drinking water as putative contributory factors to endemic goitre in Plateau State, Nigeria. Trop Geogr Med. 1989; 41(4): 346-52 as it appears in World Health Organization (WHO). WHO Global Database on Iodine Deficiency. Geneva, Switzerland: World Health Organization (WHO)
- Dawaki S, Al-Mekhlafi HM, Ithoi I, Ibrahim J, Atroosh WM, Abdulsalam AM, Sady H, Elyana FN, Adamu AU, Yelwa SI, Ahmed A, Al-Areeqi MA, Subramaniam LR, Nasr NA, Lau Y-L. Is Nigeria winning the battle against malaria? Prevalence, risk factors and KAP assessment among Hausa communities in Kano State. Malar J. 2016; 15: 351 as it appears in Malaria Atlas Project. Malaria Atlas Project Plasmodium Falciparum Parasite Rate Database. Oxford, United Kingdom: Malaria Atlas Project
- Dawodu AH, Laditan AA. Low birthweight in an urban community in Nigeria. Ann Trop Paediatr. 1985; 5(2): 61-6
- Demyttenaere K, Bruffaerts R, Lee S, Posada-Villa J, Kovess V, Angermeyer MC, Levinson D, de Girolamo G, Nakane H, Mneimneh Z, Lara C, de Graaf R, Scott KM, Gureje O, Stein DJ, Haro JM, Bromet EJ, Kessler RC, Alonso J, Von Korff M. Mental disorders among persons with

chronic back or neck pain: results from the World Mental Health Surveys. Pain. 2007; 129(3): 332-42

- Denloye O, Popoola B, Ifesanya J. Association between dental caries and body mass index in 12¬ñ15 year old private school children in Ibadan, Nigeria. Pediatr Dent J. 2016; 26(1): 28¬ñ33
- Department for International Development (DFiD) (United Kingdom), National Population Commission of Nigeria, ORC Macro, United Nations Children's Fund (UNICEF), United Nations Population Fund (UNFPA). Nigeria Demographic and Health Survey 2003. Fairfax, United States of America: ICF International
- Department of Statistics (Nigeria). Nigeria Population Census 1952-1953
- Dogara MM, Nock HI, Agbede RIS, Ndams SI, Joseph KK. Prevalence Of Lymphatic Filariasis In Three Villages In Kano State, Nigeria. Internet J Trop Med. 2012; 8(1) as it appears in London School of Hygiene and Tropical Medicine. Global Atlas of Helminth Infections - Lymphatic Filariasis. London, United Kingdom: London School of Hygiene and Tropical Medicine
- Dunmade AD, Segun-Busari S, Olajide TG, Ologe FE. Profound bilateral sensorineural hearing loss in Nigerian children: any shift in etiology?. J Deaf Stud Deaf Educ. 2007; 12(1): 112-8
- Ebenezer A, Amadi EC, Agi PI. Studies on the microfilaria, antigenemia and clinical signs of bancroftian filariasis in Epie creek communities, Niger Delta, Nigeria. Int Res J Microbiol. 2011; 2(9): 370-4 as it appears in London School of Hygiene and Tropical Medicine. Global Atlas of Helminth Infections - Lymphatic Filariasis. London, United Kingdom: London School of Hygiene and Tropical Medicine
- Edetanlen BE, Saheeb BD. Blood lead concentrations as a result of retained lead pellets in the craniomaxillofacial region in Benin City, Nigeria. Br J Oral Maxillofac Surg. 2016; 54(5): 551-5
- Edungbola LD, Watts S. An outbreak of dracunculiasis in a peri-urban community of Ilorin, Kwara State, Nigeria. Acta Trop. 1984; 41(2): 155¬ñ63
- Eguzo KN, Lawal AK, Eseigbe CE, Umezurike CC. Determinants of Mortality among Adult HIV-Infected Patients on Antiretroviral Therapy in a Rural Hospital in Southeastern Nigeria: A 5-Year Cohort Study. AIDS Res Treat. 2014
- Eigege A, Richards FO, Blaney DD, Miri ES, Gontor I, Ogah G, Umaru J, Jinadu MY, Mathai W, Amadiegwu S, Hopkins DR. Rapid assessment for lymphatic filariasis in central Nigeria: A comparison of the immunochromatographic card test and hydrocele rates in an area of high endemicity. Am J Trop Med Hyg. 2003; 68(6): 643-6
- Ejezie GC, Akpan IF. Human ecology and parasitic infections. 1. The effect of occupation on the prevalence of parasitic infections in Calabar, Nigeria. J Hyg Epidemiol Microbiol Immunol. 1992; 36(2): 161-7 as it appears in Malaria Atlas Project. Malaria Atlas Project Plasmodium Falciparum Parasite Rate Database. Oxford, United Kingdom: Malaria Atlas Project
- Ejezie GC, Ezedinachi ENU, Usanga EA, Gemade EII, Ikpatt NW, Alaribe AAA. Malaria and its treatment in rural villages of Aboh Mbaise, Imo State, Nigeria. Acta Trop. 1990; 48(1): 17¬ñ24 as it appears in Malaria Atlas Project. Malaria Atlas Project Plasmodium Falciparum Parasite Rate Database. Oxford, United Kingdom: Malaria Atlas Project
- Ejezie GC, Onyezili NI, Okeke GC, Enwonwu CO. Ijanikin: a study of environmental health in a rural Nigerian community. J Hyg Epidemiol Microbiol Immunol. 1987; 31(2): 163-72 as it appears in Malaria Atlas Project. Malaria Atlas Project Plasmodium Falciparum Parasite Rate Database. Oxford, United Kingdom: Malaria Atlas Project

- Ejim EC, Onwubere BJ, Okafor CI, Ulasi II, Emehel A, Onyia U, Akabueze J, Mendis S. Cardiovascular risk factors in middle-aged and elderly residents in South-East Nigeria: the influence of urbanization. Niger J Med. 2013; 22(4): 286¬ñ91
- Ejimadu CS, Adio AO. The burden of low vision in farming communities in South-South Nigeria. Niger J Med. 2012; 21(2): 218-22
- Ekanem EE, Asindi AA, Okoi OU. Community-based surveillance of paediatric deaths in Cross River State, Nigeria. Trop Geogr Med. 1994; 46(5): 305-8
- Ekanem EE, Ochigbo SO, Kwagtsule JU. Unprecedented decline in measles morbidity and mortality in Calabar, south-eastern Nigeria. Trop Doct. 2000; 30(4): 207¬ñ9
- Ekanem US, Opara DC, Akwaowo CD. High blood pressure in a semi-urban community in south-south Nigeria: a community-based study. Afr Health Sci. 2013; 13(1): 56¬ñ61
- Eke AC, Ezebialu IU, Okafor C. Presentation and outcome of eclampsia at a tertiary center in South East Nigeria--a 6-year review. Hypertens Pregnancy. 2011; 30(2): 125-32
- Ekenna O, Chikwem JO, Mohammed I, Durojaiye SO. Epidemic yellow fever in Borno State of Nigeria: characterisation of hospitalised patients. West Afr J Med. 2010; 29(2): 91-7
- Ekpenyong EA, Eyo JE. Malaria control and treatment strategies among school children in semi-urban tropical communities. West Indian Med J. 2008; 57(5): 456-61 as it appears in Malaria Atlas Project. Malaria Atlas Project Plasmodium Falciparum Parasite Rate Database. Oxford, United Kingdom: Malaria Atlas Project
- Ekrikpo UE, Udo AI, Ikpeme EE, Effa EE. Haemodialysis in an emerging centre in a developing country: a two year review and predictors of mortality. BMC Nephrol. 2011; 12: 50
- Eneanya CI. Seasonal variation in malaria episodes among residents in a semi-urban community in South-East Nigeria. Niger J Parasitol. 1998; 19: 39-43 as it appears in Malaria Atlas Project. Malaria Atlas Project Plasmodium Falciparum Parasite Rate Database. Oxford, United Kingdom: Malaria Atlas Project
- Engelbrecht F, T √ ðgel E, Beck H-P, Enwezor F, Oettli A, Felger I. Analysis of Plasmodium falciparum infections in a village community in Northern Nigeria: determination of msp2 genotypes and parasite-specific IgG responses. Addict Behav Rep. 2000; 74(1): 63-71 as it appears in Malaria Atlas Project. Malaria Atlas Project Plasmodium Falciparum Parasite Rate Database. Oxford, United Kingdom: Malaria Atlas Project
- Enosolease ME, Bazuaye GN. Distribution of ABO and Rh-D blood groups in the Benin area of Niger-Delta: Implication for regional blood transfusion. Asian J Transfus Sci. 2008; 2(1): 3-5
- Esan O, Makanjuola V, Oladeji B, Gureje O. Determinants of transition across the spectrum of alcohol use and misuse in Nigeria. Alcohol. 2013; 47(3): 249¬ñ55
- Eteng MU. Effect of Plasmodium falciparum parasitaemia on some haematological parameters in adolescent and adult Nigerian HbAA and HbAS blood genotypes. Cent Afr J Med. 2002; 48(11-12): 129-32 as it appears in Malaria Atlas Project. Malaria Atlas Project Plasmodium Falciparum Parasite Rate Database. Oxford, United Kingdom: Malaria Atlas Project
- Etukumana EA, Puepet FH, Obadofin MO. Risk factors for diabetes mellitus among rural adults in Nigeria. Niger J Med. 2014; 23(3): 213¬ñ9
- Euromonitor International. Euromonitor Passport - Alcoholic Drinks Statistics . London, United Kingdom: Euromonitor International
- Euromonitor International. Euromonitor Passport - Smoking Tobacco Statistics . London, United Kingdom: Euromonitor International

- Euromonitor Passport - Fresh Foods Market Statistics as it appears in Euromonitor International. Euromonitor Passport - Fresh Foods Market Statistics. London, United Kingdom: Euromonitor International
- Expanded Social Marketing Project in Nigeria (ESMPIN), Federal Ministry of Health (Nigeria), Joint United Nations Program on HIV/AIDS (UNAIDS), National Population Commission (NPC), Society for Family Health (Nigeria), University College Hospital, Ibadan, World Health Organization (WHO). Nigeria National HIV/AIDS and Reproductive Health Survey 2012
- Ezeaka VC, Iroha EO, Akinsulie AO, Temiye EO, Adetifa IMO. Anthropometric indices of infants born to HIV-1-infected mothers: a prospective cohort study in Lagos, Nigeria. Int J STD AIDS. 2009; 20(8): 545-8
- Ezechi OC, Ndububa VI, Loto OM, Ezeobi PM, Kalu BKE, Njokanma OF, Nwokoro CA. Pregnancy, obstetric and neonatal outcome after assisted reproduction in Nigerians. J Matern Fetal Neonatal Med. 2008; 21(4): 261-6
- Ezedinachi EN, Ejezie GC, Usanga EA, Gemade EI, Ikpatt NW, Alaribe AA. New trends in chloroquine efficacy in the treatment of malaria: chloroquine-resistant Plasmodium falciparum in Anambra and Benue States of Nigeria. Cent Afr J Med. 1991; 37(6): 180-6 as it appears in Malaria Atlas Project. Malaria Atlas Project Plasmodium Falciparum Parasite Rate Database. Oxford, United Kingdom: Malaria Atlas Project
- Ezugwu EC, Onah HE, Ezugwu FO, Okafor II. Maternal mortality in a transitional hospital in Enugu, south east Nigeria. Afr J Reprod Health. 2009; 13(4): 67-72
- Fafowora OF. Prevalence of blindness in a rural ophthalmically underserved Nigerian community. West Afr J Med. 1996; 15(4): 228-31
- Falade AG, Ige OM, Yusuf BO, Onadeko MO, Onadeko BO. Trends in the prevalence and severity of symptoms of asthma, allergic rhinoconjunctivitis, and atopic eczema. J Natl Med Assoc. 2009; 101(5): 414-8
- Falade CO, Nash O, Akingbola TS, Michael OS, Olojede F, Ademowo OG. Blood banking in a malaria-endemic area: evaluating the problem posed by malarial parasitaemias. Ann Trop Med Parasitol. 2009; 103(5): 383-92 as it appears in Malaria Atlas Project. Malaria Atlas Project Plasmodium Falciparum Parasite Rate Database. Oxford, United Kingdom: Malaria Atlas Project
- FAO Supply Utilization Accounts 1961-2013 as it appears in FAO Supply Utilization Accounts 1961-2013. Personal Correspondence with Dr. Josef Schmidhuber, 2016. [Unpublished]
- FAO Supply Utilization Accounts 1961-2013. Personal Correspondence with Dr. Josef Schmidhuber, 2016. [Unpublished]
- FAOSTAT Food Balance Sheets, October 2014 as it appears in Food and Agriculture Organization of the United Nations (FAO). FAOSTAT Food Balance Sheets, October 2014. Rome, Italy: Food and Agriculture Organization of the United Nations (FAO)
- Fatiregun AA, Olowookere SA, Abubakar O, Aderibigbe A. Small-scale outbreak of measles in the Irewole local government area of Osun State in Nigeria. Asian Pac J Trop Med. 2009; 2(6): 33¬ñ6
- Fawibe AE. The pattern and outcome of adult tetanus at a sub-urban tertiary hospital in Nigeria. J Coll Physicians Surg Pak. 2010; 20(1): 68-70
- Federal Ministry of Health (Nigeria), ICF International, National Population Commission (NPC). Nigeria Demographic and Health Survey 2018. Fairfax, United States of America: ICF International, 2020

- Federal Ministry of Health (Nigeria), Macro Systems, Inc.; Institute for Resource Development, National Population Bureau (Nigeria). Nigeria - Ondo Special Demographic and Health Survey 1986-1987. Fairfax, United States of America: ICF International
- Federal Ministry of Health (Nigeria), National Agency for the Control of AIDS (NACA) (Nigeria), National Bureau of Statistics (Nigeria), National Population Commission (NPC), Nigerian Institute of Medical Research, Society for Family Health (Nigeria), University College Hospital, Ibadan. Nigeria National HIV/AIDS and Reproductive Health Survey 2007
- Federal Ministry of Health (Nigeria), Nigerian Heart Foundation, World Health Organization (WHO). Nigeria - Lagos STEPS Noncommunicable Disease Risk Factors Survey 2003
- Federal Ministry of Health (Nigeria). Nigeria National HIV/AIDS and Reproductive Health Survey 2005
- Federal Ministry of Health (Nigeria). Nigeria National HIV/Syphilis Seroprevalence Sentinel Survey 2005
- Federal Ministry of Health [Nigeria]. 2003. National HIV/AIDS and Reproductive Health Survey, 2003. Federal Ministry of Health Abuja, Nigeria
- Federal Ministry of Water Resources (Nigeria), National Water Resources Institute (Nigeria), United Nations Children's Fund (UNICEF), World Health Organization (WHO). Nigeria Rapid Assessment of Drinking-Water Quality Pilot 2004-2005. Geneva, Switzerland: WHO/UNICEF Joint Monitoring Programme for Water Supply and Sanitation
- Federal Office of Statistics (Nigeria), Institute for Resource Development, Macro Systems. Nigeria Demographic and Health Survey 1990. Fairfax, United States of America: ICF International
- Federal Office of Statistics (Nigeria), Macro International, Inc, National Population Commission of Nigeria, UK Department for International Development (DFID), United Nations Children's Fund (UNICEF), United Nations Population Fund (UNFPA). Nigeria Demographic and Health Survey - Complete Birth History Data
- Federal Office of Statistics (Nigeria), United Nations Children's Fund (UNICEF). Nigeria Multiple Indicator Cluster Survey 1995
- Federal Office of Statistics (Nigeria). Nigeria Living Standards Survey 2003-2004
- Feenstra, Robert C., Robert Inklaar and Marcel P. Timmer (2015), "The Next Generation of the Penn World Table" American Economic Review, 105(10), 3150-3182, available for download at www.ggdc.net/pwt
- Fetuga BM, Ogunlesi TA, Adekanmbi FA. Risk factors for mortality in neonatal tetanus: a 15-year experience in Sagamu, Nigeria. World J Pediatr. 2010; 6(1): 71-5
- Fetuga MB, Njokanma OF, Ogunfowora OB, Runsewe-Abiodun. A ten-year study of measles admissions in a Nigerian teaching hospital. Niger J Clin Pract. 2007; 10(1): 41¬ñ6
- Food and Agriculture Organization of the United Nations (FAO). FAOSTAT Commodity Balances - Crops Primary Equivalent. Rome, Italy: Food and Agriculture Organization of the United Nations (FAO)
- Food Fortification Initiative (United States), Global Alliance for Improved Nutrition (GAIN), Iodine Global Network (Canada), Micronutrient Forum (Canada). Global Fortification Data Exchange: Legislation and Standards, Year When Food Fortification Mandated, and Count of Nutrients In Fortification Standards
- Forbi JC, Purdy MA, Campo DS, Vaughan G, Dimitrova ZE, Ganova-Raeva LM, Xia G-L, Khudyakov YE. Epidemic history of hepatitis C virus infection in two remote communities in Nigeria, West Africa. J Gen Virol. 2012; 93(Pt 7): 141021

- Forrester T, Adeyemo A, Soarres-Wynter S, Sargent L, Bennett F, Wilks R, Luke A, Prewitt E, Kramer H, Cooper RS. A randomized trial on sodium reduction in two developing countries. J Hum Hypertens. 2005; 19(1): 55-60 as it appears in Forrester T, Adeyemo A, Soarres-Wynter S, Sargent L, Bennett F, Wilks R, Luke A, Prewitt E, Kramer H, Cooper RS. A randomized trial on sodium reduction in two developing countries. J Hum Hypertens. 2005; 19(1): 55-60
- Franceschi S, Smith JS, van den Brule A, Herrero R, Arslan A, Anh P-T-H, Bosch FX, Hieu N-T, Matos E, Posso H, Qiao Y-L, Shin H-R, Sukvirach S, Thomas JO, Snijders PJF, Mu √ ±oz N, Meijer CJLM. Cervical infection with Chlamydia trachomatis and Neisseria gonorrhoeae in women from ten areas in four continents. A cross-sectional study. Sex Transm Dis. 2007; 34(8): 563-9
- Gallup. Nigeria World Poll 2005-2006
- Gallup. Nigeria World Poll 2007
- Garba JA, Umar S. Aetiology of maternal mortality using verbal autopsy at Sokoto, North-Western Nigeria. Afr J Prim Health Care Fam Med. 2013; 5(1): 6 pages
- Garcia-Marcos L, Robertson CF, Ross Anderson H, Ellwood P, Williams HC, Wong GW. Does migration affect asthma, rhinoconjunctivitis and eczema prevalence? Global findings from the international study of asthma and allergies in childhood. Int J Epidemiol. 2014; 43(6): 1846-54
- Ghana Center for Democratic Development (CDD-Ghana), Institute for Development Studies, University of Nairobi, Institute for Empirical Research in Political Economy (IERPE), Institute for Justice and Reconciliation (South Africa), Michigan State University, Practical Sampling International (PSI) (Nigeria), University of Cape Town. Nigeria Afrobarometer: The Quality of Democracy and Governance in Nigeria Round 7 2017. Afrobarometer
- Ghana Center for Democratic Development (CDD-Ghana), Institute for Development Studies, University of Nairobi, Institute for Empirical Research in Political Economy (IERPE), Institute for Justice and Reconciliation (South Africa), Michigan State University, Practical Sampling International (PSI) (Nigeria). Nigeria Afrobarometer: The Quality of Democracy and Governance in Nigeria Round 6 2014-2015. Afrobarometer, 2017
- Ghatak DP. A study of urinary ?stulae in Sokoto, Nigeria. J Indian Med Assoc. 1992; 80(11): 285-7 as it appears in Zheng A, Anderson F. Obstetric fistula in low-income countries. Int J Gynaecol Obstet. 2009; 104: 85-9
- Global Burden of Disease 2010 Expert Group Working Document on Sense and Hearing Disorders
- Global Burden of Disease Collaborative Network. Global Burden of Disease Study 2016 (GBD 2016) Covariates 1980-2016. Seattle, United States: Institute for Health Metrics and Evaluation (IHME), 2017
- Global Burden of Disease Collaborative Network. Global Burden of Disease Study 2017 (GBD 2017) Cause-Specific Mortality 1980-2017. Seattle, United States: Institute for Health Metrics and Evaluation (IHME), 2018
- Global Burden of Disease Collaborative Network. Global Health Spending 1995-2017. Seattle, United States of America: Institute for Health Metrics and Evaluation (IHME), 2020
- Goon DT, Toriola AL, Shaw BS. Screening for body-weight disorders in Nigerian children using contrasting definitions. Obes Rev. 2010; 11(7): 508-15
- Grais RF,¬†Dubray C,¬†Gerstl S,¬†Guthmann JP,¬†Djibo A,¬†Nargaye KD,¬†Coker J,¬†Alberti KP,¬†Cochet A,¬†Ihekweazu C,¬†Nathan N,¬†Payne L,¬†Porten K,¬†Sauvageot D,Schimmer B,¬†Fermon F,¬†Burny ME,¬†Hersh BS,¬†Guerin PJ. Unacceptably high mortality related to measles epidemics in Niger, Nigeria, and Chad. PLoS Med. 2007; 4(1): e16

- Gundiri MA, Lombonyi CA, Akogun OB. Malaria in an obligate nomadic Fulani camps in Adamawa State, north-eastern Nigeria. Niger J Parasitol. 2008; 28(2): 87-89 as it appears in Malaria Atlas Project. Malaria Atlas Project Plasmodium Falciparum Parasite Rate Database. Oxford, United Kingdom: Malaria Atlas Project
- Gureje O, Kola L, Afolabi E. Epidemiology of major depressive disorder in elderly Nigerians in the Ibadan Study of Ageing: a community-based survey. Lancet. 2007; 370(9591): 957-64
- Gureje O, Lasebikan VO, Kola L, Makanjuola VA. Lifetime and 12-month prevalence of mental disorders in the Nigerian Survey of Mental Health and Well-Being. Br J Psychiatry. 2006; 188(5): 465-71
- Harris JB, Larocque RC, Charles RC, Mazumder RN, Khan AI, Bardhan PK. Cholera¬ís western front. Lancet. 2010; 376(9757): 1961¬ñ5
- Health Emergencies Programme, World Health Organization Regional Office for Africa (WHO-AFRO). Compendium of Short Reports on Selected Outbreaks in the WHO African Region, October 2017 and Weekly Bulletins of Outbreaks and Other Emergencies 2017-2018.
- Henshaw EB, Olasode OA, Ogedegbe EE, Etuk I. Dermatologic conditions in teenage adolescents in Nigeria. Adolesc Health Med Ther. 2014; 5: 79¬ñ87
- Hilton P, Ward A. Epidemiological and surgical aspects of urogenital ?stulae: a review of 25 years' experience in southeast Nigeria. Int Urogynecol J Pelvic Floor Dysfunct. 1989; 9(4): 189-94 as it appears in Zheng A, Anderson F. Obstetric fistula in low-income countries. Int J Gynaecol Obstet. 2009; 104: 85-9
- Hopkins DR, Ruiz-Tiben E, Ruebush TK, Diallo N, Agle A, Withers PC Jr. Dracunculiasis eradication: delayed, not denied. Am J Trop Med Hyg. 2000; 62(2): 163-8
- Houben CH, Fleischmann H, G √ ºckel M. Malaria prevalence in north-eastern Nigeria: a cross-sectional study. Asian Pac J Trop Med. 2013; 6(11): 865-8 as it appears in Malaria Atlas Project. Malaria Atlas Project Plasmodium Falciparum Parasite Rate Database. Oxford, United Kingdom: Malaria Atlas Project
- Hult M, Tornhammar P, Ueda P, Chima C, Bonamy A-KE, Ozumba B, Norman M. Hypertension, diabetes and overweight: looming legacies of the Biafran famine. PLoS One. 2010; 5(10): e13582
- Human African Trypanosomiasis At-Risk Population Estimates 1980-2015, as provided by the Global Burden of Disease 2010 Nematode expert group. [Unpublished]
- I O George, A I Frank-Briggs. Pattern and clinical presentation of congenital heart diseases in Port-Harcourt. Niger J Med. 2008; 18(2): 211-4
- Ibadin MO, Ogbimi A. Antityphoid agglutinins in African school aged children with malaria. West Afr J Med. 2004; 23(4): 276-9 as it appears in Malaria Atlas Project. Malaria Atlas Project Plasmodium Falciparum Parasite Rate Database. Oxford, United Kingdom: Malaria Atlas Project
- Ibia EO, Asindi AA. Measles in Nigerian Children in Calabar During the Era of Expanded Program on Immunization. Trop Geogr Med. 1990; 42(3): 226¬ñ32
- Iboh CI, Okon OE, Opara KN, Asor JE, Etim SE. Lymphatic filariasis among the Yakurr people of Cross River State, Nigeria. Parasites Vectors. 2012; 203 as it appears in London School of Hygiene and Tropical Medicine. Global Atlas of Helminth Infections - Lymphatic Filariasis. London, United Kingdom: London School of Hygiene and Tropical Medicine
- Ibrahim BS, Usman R, Mohammed Y, Datti Z, Okunromade O, Abubakar AA, Nguku PM. Burden of measles in Nigeria: a five-year review of casebased surveillance data, 2012-2016. Pan Afr Med J. 2019; 32(Suppl 1): 5

- Ibrahim T, Sadiq AU, Daniel SO. Characteristics of VVF patients as seen at the specialist hospital Sokoto, Nigeria. West Afr J Med. 2000; 19(1): 59-63 as it appears in Zheng A, Anderson F. Obstetric fistula in low-income countries. Int J Gynaecol Obstet. 2009; 104: 85-9
- ICF International, National Bureau of Statistics (Nigeria), National Malaria Control Programme (Nigeria), National Population Commission of Nigeria. Nigeria Malaria Indicator Survey 2015. Fairfax, United States of America: ICF International, 2016
- ICF International, National Population Commission of Nigeria. Nigeria Demographic and Health Survey 2013. Fairfax, United States of America: ICF International
- ICF Macro, National Malaria Control Programme (Nigeria), National Population Commission of Nigeria. Nigeria Malaria Indicator Survey 2010. Fairfax, United States of America: ICF International
- Igbeneghu C, Odaibo AB, Olaleye DO. Impact of asymptomatic malaria on some hematological parameters in the Iwo community in Southwestern Nigeria. Med Princ Pract. 2011; 20(5): 459-63 as it appears in Malaria Atlas Project. Malaria Atlas Project Plasmodium Falciparum Parasite Rate Database. Oxford, United Kingdom: Malaria Atlas Project
- Igwegbe AO, Eleje GU, Ugboaja JO, Ofiaeli RO. Improving maternal mortality at a university teaching hospital in Nnewi, Nigeria. Int J Gynaecol Obstet. 2012; 116(3): 197-200
- Ijaiya MA, Aboyeji PA. Obstetric urogenital fistula: the Ilorin experience, Nigeria. West Afr J Med. 2004; 23(1): 7-9
- Ijaiya MA, Aboyeji PA. Obstetric urogenital fistula: the Ilorin experience, Nigeria. West Afr J Med. 2004; 23(1): 7-9 as it appears in Zheng A, Anderson F. Obstetric fistula in low-income countries. Int J Gynaecol Obstet. 2009; 104: 85-9
- Ikaraoha CI, Mbadiwe NC, Anyanwu CJ, Odekhian J, Nwadike CN, Amah HC. The Role of Blood Lead, Cadmium, Zinc and Copper in Development and Severity of Acne Vulgaris in a Nigerian Population. Biol Trace Elem Res. 2016; nan
- Ilegbodu VA, Christensen BL, Wise RA, Kale O, Steele JH, Chambers LA. Age and sex differences in new and recurrent cases of guinea worm disease in Nigeria. Trans R Soc Trop Med Hyg. 1987; 81(4): 674-676
- Iliyasu Z, Abubakar IS, Abubakar S, Lawan UM, Gajida AU, Jibo AM. A survey of weight perception and social desirability of obesity among adults in Kano Metropolis, Northern Nigeria. Niger J Med. 2013; 22(2): 101-8
- Insecticide Treated Net Procurement Data, Personal Communication with the World Health Organization 2016 as it appears in Malaria Atlas Project. Malaria Atlas Project Interventions Database
- Insecticide Treated Net Procurement Data, Personal Communication with the World Health Organization 2017 as it appears in Malaria Atlas Project. Malaria Atlas Project Interventions Database
- Insecticide Treated Nets Manufactured by Country, Personal Communication with the World Health Organization 2019 as it appears in Malaria Atlas Project. Malaria Atlas Project Interventions Database
- Institute of Social Medicine and Health Policy, Shandong University, Shandong University School of Medicine, World Health Organization (WHO). China WHO Multi-country Survey Study on Health and Health System Responsiveness 2000-2001
- International Fuel Quality Center (IFQC). Overview of Leaded Gasoline and Sulfur Levels in Gasoline and Diesel . 2002
- International Institute of Tropical Agriculture (IITA), and HarvestPlus of International Food Policy Research Institute (IFPRI). 2015. Dietary intakes, vitamin A, and iron status of women

of childbearing age and children 6-59 months of age from Akwa Ibom state in Nigeria. Washington, DC: International Food Policy Research Institute (IFPRI) [datasets]. http://dx.doi.org/10.7910/DVN/29604

- International Labour Organization (ILO). International Labour Organization Database (ILOSTAT) - Employment by Sex and Economic Activity. International Labour Organization (ILO)
- International Labour Organization (ILO). International Labour Organization Database (ILOSTAT) - Employment by Sex and Occupation. International Labour Organization (ILO)
- International Labour Organization (ILO). International Labour Organization Database (ILOSTAT) - Employment to Population Ratio by Sex and Age. International Labour Organization (ILO)
- International Monetary Fund (IMF). World Economic Outlook Database. Washington, D.C., United States of America: International Monetary Fund (IMF)
- International Road Federation. World Road Statistics 1963-1999. Geneva, Switzerland: International Road Federation
- International Road Federation. World Road Statistics 2009 Database and World Road Statistics 2007
- International Statistical Institute, National Population Bureau (Nigeria). Nigeria World Fertility Survey 1981-1982
- Iriemenam NC, Okafor CMF, Balogun HA, Ayede I, Omosun Y, Persson J-O, Hagstedt M, Anumudu CI, Nwuba RI, Troye-Blomberg M, Berzins K. Cytokine profiles and antibody responses to Plasmodium falciparum malaria infection in individuals living in Ibadan, southwest Nigeria. Afr Health Sci. 2009; 9(2): 66-74 as it appears in Malaria Atlas Project. Malaria Atlas Project Plasmodium Falciparum Parasite Rate Database. Oxford, United Kingdom: Malaria Atlas Project
- Iroezindu MO, Agaba EI, Okeke EN, Daniyam CA, Obaseki DO, Isa SE, Idoko JA. Prevalence of malaria parasitaemia in adult HIV-infected patients in Jos, North-central Nigeria. Niger J Med. 2012; 21(2): 209¬ñ13 as it appears in Malaria Atlas Project. Malaria Atlas Project Plasmodium Falciparum Parasite Rate Database. Oxford, United Kingdom: Malaria Atlas Project
- Isara AR, Okundia PO. The burden of hypertension and diabetes mellitus in rural communities in southern Nigeria. Pan Afr Med J. 2015; 103
- Isezuo SA, Sabir AA, Ohwovorilole AE, Fasanmade OA. Prevalence, associated factors and relationship between prehypertension and hypertension: a study of two ethnic African populations in Northern Nigeria. J Hum Hypertens. 2011; 25(4): 224¬ñ30
- Jeremiah ZA, Odumody C. Rh antigens and phenotype frequencies of the Ibibio, Efik, and Ibo ethnic nationalities in Calabar, Nigeria. Immunohematology. 2005; 21(1): 21-4
- Jeremiah ZA, Uko E. Childhood asymptomatic malaria and nutritional status among Port Harcourt children. East Afr J Public Health. 2007; 4(2): 55-8 as it appears in Malaria Atlas Project. Malaria Atlas Project Plasmodium Falciparum Parasite Rate Database. Oxford, United Kingdom: Malaria Atlas Project
- Jido TA. Ecalmpsia: maternal and fetal outcome. Afr Health Sci. 2012; 12(2): 148-52
- Joint United Nations Program on HIV/AIDS (UNAIDS), National Agency for the Control of AIDS (Nigeria). Nigeria Global AIDS Response Country Progress Report 2012. Geneva, Switzerland: Joint United Nations Program on HIV/AIDS (UNAIDS), 2012

- Joint United Nations Program on HIV/AIDS (UNAIDS), United Nations Children's Fund (UNICEF), World Health Organization (WHO). Nigeria Epidemiological Fact Sheet on HIV/AIDS and STIs 2004
- Joint United Nations Program on HIV/AIDS (UNAIDS), United Nations Children's Fund (UNICEF), World Health Organization (WHO). Nigeria Global AIDS Response Progress Reporting (GARPR) System - Antenatal Care Attendees Positive for Syphilis
- Kale, Oladele O. The clinico-epidemiological profile of guinea worm in the Ibadan district of Nigeria. Am J Trop Med Hyg. 1977; 26(2): 208-214
- Karaye KM, Sani MU. Factors associated with poor prognosis among patients admitted with heart failure in a Nigerian tertiary medical centre: a cross-sectional study. BMC Cardiovasc Disord. 2008; 8(1): 16
- Keating EM, Fischer PR, Pettifor JM, Pfitzner M, Isichei CO, Thacher TD. The effect of calcium supplementation on blood lead levels in Nigerian children. J Pediatr. 2011; 159(5): 845-850
- Kolawole OU, Ashaye AO, Mahmoud AO, Adeoti CO. Cataract blindness in Osun state, Nigeria: results of a survey. Middle East Afr J Ophthalmol. 2012; 19(4): 364-71
- Kullima AA, Kawuwa MB, Audu BM, Geidam AD, Mairiga AG. Trends in maternal mortality in a tertiary institution in Northern Nigeria. Ann Afr Med. 2009; 8(4): 221-4
- Kurz X. The yellow fever epidemic in Western Mali, September-November 1987: Why did epidemiological surveillance fail? Disasters. 1990; 14(1): 46-54
- Kuti O, Owa JA. Gestational age-specific neonatal mortality among preterm singleton births in a Nigerian tertiary institution. Int J Gynaecol Obstet. 2003; 80(3): 319-20
- Kyari F, Gudlavalleti MVS, Sivsubramaniam S, Gilbert CE, Abdull MM, Entekume G, Foster A. Prevalence of blindness and visual impairment in Nigeria: the National Blindness and Visual Impairment Study. Invest Ophthalmol Vis Sci. 2009; 50(5): 2033-9
- Labinjo M, Juillard C, Kobusingye OC, Hyder AA. The burden of road traffic injuries in Nigeria: results of a population-based survey. Inj Prev. 2009; 15(3): 157-62
- Lagunju IA1,¬†Orimadegun AE,¬†Oyedemi DG. Measles¬†in¬†Ibadan: a¬†continuous¬†scourge. Afr J Med Med Sci. 2005; 34(4): 383-7
- Lawal F, Alade O. Dental caries experience and treatment needs of an adult female population in Nigeria. Afr Health Sci. 2017; 17(3): 905-911
- Lawoyin TO, Asuzu MC, Kaufman J, Rotimi C, Johnson L, Owoaje E, Cooper R. Using verbal autopsy to identify and proportionally assign cause of death in Ibadan, southwest Nigeria. Niger Postgrad Med J. 2004; 11(3): 182-6
- Lawoyin TO, Oyediran AB. A prospective study on some factors which influence the delivery of low birth weight babies in a developing country. Afr J Med Med Sci. 1992; 21(1): 33-9
- London School of Hygiene and Tropical Medicine. Global Atlas of Helminth Infections - Lymphatic Filariasis. London, United Kingdom: London School of Hygiene and Tropical Medicine
- Luzzatto L, Allan NC. Relationship between the genes for glucose-6-phosphate dehydrogenase and for haemoglobin in a Nigerian population. Nature. 1968; 219(5158): 1041-2
- Macro International, Inc, National Population Commission of Nigeria. Nigeria Demographic and Health Survey 1999. Calverton, United States of America: Macro International, Inc
- Macro International, Inc, National Population Commission of Nigeria. Nigeria Demographic and Health Survey 2008. Fairfax, United States of America: ICF International, 2009

- Maddison Project Database, version 2018. Bolt, Jutta, Robert Inklaar, Herman de Jong and Jan Luiten van Zanden (2018), ¬ìRebasing ¬ëMaddison¬í: new income comparisons and the shape of long-run economic development¬î, Maddison Project Working paper 10
- Mairiga AG, Saleh W. Maternal mortality at the State Specialist Hospital Bauchi, Northern Nigeria. East Afr Med J. 2009; 86(1): 25-30
- Makusidi MA, Liman HM, Yakubu A, Isah MD, Abdullahi S, Chijioke A. Hemodialysis performance and outcomes among end stage renal disease patients from Sokoto, North-Western Nigeria. Indian J Nephrol. 2014; 24(2): 82¬ñ5
- Makusidi MA, Liman HM, Yakubu A, Isah MDA, Jega RM, Adamu H, Chijioke A. Prevalence of non-communicable diseases and its awareness among inhabitants of Sokoto metropolis: outcome of a screening program for hypertension, obesity, diabetes mellitus and overt proteinuria. Arab J Nephrol Transplant. 2013; 6(3): 189¬ñ91
- Malaria 1982-1997 as it appears in Malaria Atlas Project. Malaria Atlas Project Annual Parasite Incidence Database
- Malaria Among Asymptomatic School Children in Ezinihitte Local Government Area of Imo State, Nigeria as it appears in Malaria Atlas Project. Malaria Atlas Project Plasmodium Falciparum Parasite Rate Database. Oxford, United Kingdom: Malaria Atlas Project
- Malaria Atlas Project. Haemoglobin C (HbC) Allele Frequency Layer. Oxford, United Kingdom: Malaria Atlas Project, 2013
- Malaria Atlas Project. Malaria Atlas Project Annual Parasite Incidence Database
- Malaria Atlas Project. Malaria Atlas Project Plasmodium Falciparum Parasite Rate Database. Oxford, United Kingdom: Malaria Atlas Project
- Malaria Atlas Project. Sickle Haemoglobin (Hbs) Allele Frequency Layer. Oxford, United Kingdom: Malaria Atlas Project, 2013
- Malaria Situation in Nigeria as it appears in Malaria Atlas Project. Malaria Atlas Project Plasmodium Falciparum Parasite Rate Database. Oxford, United Kingdom: Malaria Atlas Project
- Manson J, Ameh E, Canvassar N, Chen T, den Hoeve AV, Lever F, Hesse A, Millar A, Emil S, Ade-Ajayi N. Gastroschisis: a multi-centre comparison of management and outcome. Afr J Paediatr Surg. 2012; 9(1): 17-21
- May J, Falusi AG, Mockenhaupt FP, Ademowo OG, Olumese PE, Bienzle U, Meyer CG. Impact of subpatent multi-species and multi-clonal plasmodial infections on anaemia in children from Nigeria. Trans R Soc Trop Med Hyg. 2000; 94(4): 399-403 as it appears in Malaria Atlas Project. Malaria Atlas Project Plasmodium Falciparum Parasite Rate Database. Oxford, United Kingdom: Malaria Atlas Project
- May J, Mockenhaupt FP, Ademowo OG, Falusi AG, Olumese PE, Bienzle U, Meyer CG. High rate of mixed and subpatent malarial infections in southwest Nigeria. Am J Trop Med Hyg. 1999; 61(2): 339-43 as it appears in Malaria Atlas Project. Malaria Atlas Project Plasmodium Falciparum Parasite Rate Database. Oxford, United Kingdom: Malaria Atlas Project
- Mbachu I, Udigwe GO, Okafor CI, Umeonunihu OS, Ezeama C, Eleje GU. The pattern and obstetric outcome of hypertensive disorders of pregnancy in Nnewi, Nigeria. Niger J Med. 2013; 22(2): 117-22
- MEASURE Evaluation Project, Carolina Population Center, University of North Carolina, Center for Research, Evaluation, and Resource Development (CRERD), Center for Communication Programs, Bloomberg School of Public Health, Johns Hopkins, Creative Associates International, Constella Futures, Adolescent Health and Information Project (Nigeria), Federation of Muslim Women¬ís Associations of Nigeria (FOMWAN), Nigerian

Medical Association, Management Sciences for Health (MSH), Civil Society Action Coalition on Education For All. Nigeria Reproductive Health, Child Health, and Education Household, School, and Health Facility Baseline Surveys 2005. Chapel Hill, United States: MEASURE Evaluation Project, Carolina Population Center, University of North Carolina

- MEASURE Evaluation Project, Carolina Population Center, University of North Carolina, Center for Research, Evaluation, and Resource Development (CRERD), Center for Communication Programs, Bloomberg School of Public Health, Johns Hopkins, Creative Associates International, Constella Futures, Adolescent Health and Information Project (Nigeria), Federation of Muslim Women¬ís Associations of Nigeria (FOMWAN), Nigerian Medical Association, Management Sciences for Health (MSH), Civil Society Action Coalition on Education For All. Nigeria Reproductive Health, Child Health, and Education Household, School, and Health Facility Midline Surveys 2007. Chapel Hill, United States: MEASURE Evaluation Project, Carolina Population Center, University of North Carolina
- MEASURE Evaluation Project, Carolina Population Center, University of North Carolina, Center for Research, Evaluation, and Resource Development (CRERD), Center for Communication Programs, Bloomberg School of Public Health, Johns Hopkins, Creative Associates International, Futures Group International, Adolescent Health and Information Project (Nigeria), Federation of Muslim Women¬ís Associations of Nigeria (FOMWAN), Nigerian Medical Association, Management Sciences for Health (MSH), Civil Society Action Coalition on Education For All. Nigeria Reproductive Health, Child Health, and Education Household, School, and Health Facility End-of-Project Surveys. Chapel Hill, United States: MEASURE Evaluation Project, Carolina Population Center, University of North Carolina
- Michael IO, Gabreil OE. Chronic renal failure in children of benin, Nigeria. Saudi J Kidney Dis Transpl. 2004; 15(1): 79¬ñ83
- Miri ES, Hopkins DR, Ruiz-Tiben E, Keana AS, Withers PC Jr, Anagbogu N, Sadiq LK, Kale OO, Edungbola LD, Braide El, Ologe JO, Ityonzughul C. Nigeria's triumph: dracunculiasis eradicated. Am J Trop Med Hyg. 2010; 83(2): 215-25
- Molta NB, Daniel HI, Watila IM, Oguche SO, Otu TI, Ameh JO, Gadzama NM. Efficacies of chloroquine, pyrimethamine/sulphadoxine and pyrimethamine/sulphalene against P. falciparum in northeastern Nigeria. J Trop Med Hyg. 1992; 95(4): 253-9 as it appears in Malaria Atlas Project. Malaria Atlas Project Plasmodium Falciparum Parasite Rate Database. Oxford, United Kingdom: Malaria Atlas Project
- Mosquito Control Strategies and Prevalence of Malaria in Children (0¬ñ15 years) in Amucha Community, Abia State, Nigeria as it appears in Malaria Atlas Project. Malaria Atlas Project Plasmodium Falciparum Parasite Rate Database. Oxford, United Kingdom: Malaria Atlas Project
- Murthy GVS, Fox S, Sivasubramaniam S, Gilbert CE, Mahdi AM, Imam AU, Entekume G, Nigeria National Blindness and Visual Impairment study group. Prevalence and risk factors for hypertension and association with ethnicity in Nigeria: results from a national survey. Cardiovasc J Afr. 2013; 24(9-10): 344¬ñ50
- Mustafa Adelaja L, Olufemi Taiwo O. Maternal and fetal outcome of obstetric emergencies in a tertiary health institution in South-Western Nigeria. ISRN Obstet Gynecol. 2011; 2011(160932)
- Mustapha AF, Eegunranti BA, Fawale MB. Tetanus remains a formidable health challenge in Nigeria: The experience from a single Teaching Hospital in Osun State, Nigeria. Nig Q J Hosp Med. 2015; 25(3): 151-5

- Mutebi J-P, Barrett ADT. The epidemiology of yellow fever in Africa. Microbes Infect. 2002; 4(14): 1459-68
- Mutihir JT, Utoo BT. Postpartum maternal morbidity in Jos, north-central Nigeria. Niger J Clin Pract. 2011; 14(1): 38-42
- Naicker S. Burden of end-stage renal disease in sub-Saharan Africa. Clin Nephrol. 2010; 74(Suppl 1): S13-6
- National Agency for the Control of AIDS (Nigeria), National Bureau of Statistics (Nigeria), National Primary Health Care Development Agency (NPHCDA) (Nigeria), United Nations Children's Fund (UNICEF). Nigeria Multiple Indicator Cluster Survey with National Immunization Coverage Survey Supplement 2016-2017. New York, United States of America: United Nations Children's Fund (UNICEF), 2018
- National Bureau of Statistics (Nigeria), Minnesota Population Center. Nigeria General Household Survey 2007 from the Integrated Public Use Microdata Series, International: [Machine-readable database]. Minneapolis: University of Minnesota
- National Bureau of Statistics (Nigeria), Minnesota Population Center. Nigeria General Household Survey 2008 from the Integrated Public Use Microdata Series, International: [Machine-readable database]. Minneapolis: University of Minnesota
- National Bureau of Statistics (Nigeria), Minnesota Population Center. Nigeria General Household Survey 2009 from the Integrated Public Use Microdata Series, International: [Machine-readable database]. Minneapolis: University of Minnesota
- National Bureau of Statistics (Nigeria), Minnesota Population Center. Nigeria General Household Survey 2010 from the Integrated Public Use Microdata Series, International: [Machine-readable database]. Minneapolis: University of Minnesota
- National Bureau of Statistics (Nigeria), Minnesota Population Center. Nigeria General Household Survey 2010-2011 from the Integrated Public Use Microdata Series, International: [Machine-readable database]. Minneapolis: University of Minnesota
- National Bureau of Statistics (Nigeria), National Population Commission of Nigeria, United Nations Children's Fund (UNICEF). Nigeria Standardized Monitoring and Assessment of Relief and Transitions Survey, December 2010
- National Bureau of Statistics (Nigeria), National Population Commission of Nigeria, United Nations Children's Fund (UNICEF). Nigeria Standardized Monitoring and Assessment of Relief and Transitions Survey, February-March 2012
- National Bureau of Statistics (Nigeria), National Population Commission of Nigeria, United Nations Children's Fund (UNICEF). Nigeria Standardized Monitoring and Assessment of Relief and Transitions Survey, July-August 2011
- National Bureau of Statistics (Nigeria), National Population Commission of Nigeria, United Nations Children's Fund (UNICEF). Nigeria Standardized Monitoring and Assessment of Relief and Transitions Survey, September-October 2012
- National Bureau of Statistics (Nigeria), United Nations Children's Fund (UNICEF). Nigeria Multiple Indicator Cluster Survey 1999. Abuja, Nigeria: National Bureau of Statistics (Nigeria)
- National Bureau of Statistics (Nigeria), United Nations Children's Fund (UNICEF). Nigeria Multiple Indicator Cluster Survey 2011. New York, United States of America: United Nations Children's Fund (UNICEF), 2013
- National Bureau of Statistics (Nigeria), United Nations Children's Fund (UNICEF). Nigeria Standardized Monitoring and Assessment of Relief and Transitions Survey, Round V 2013

- National Bureau of Statistics (Nigeria), United Nations Children's Fund (UNICEF). Nigeria Standardized Monitoring and Assessment of Relief and Transitions Survey, Round V 2013 - Women and Children Tabulations
- National Bureau of Statistics (Nigeria), World Bank. Nigeria General Household Survey Panel 2015-2016 Wave 3. Washington DC, United States of America: World Bank
- National Bureau of Statistics (Nigeria). National Nutrition and Health Survey 2014-v1.0
- National Bureau of Statistics (Nigeria). Nigeria Annual Abstract of Statistics 2009. Abuja, Nigeria: National Bureau of Statistics (Nigeria)
- National Bureau of Statistics (Nigeria). Nigeria Annual Abstract of Statistics 2010. Abuja, Nigeria: National Bureau of Statistics (Nigeria), 2010
- National Bureau of Statistics (Nigeria). Nigeria Core Welfare Indicators Questionnaire Survey 2006. Abuja, Nigeria: National Bureau of Statistics (Nigeria)
- National Bureau of Statistics (Nigeria). Nigeria General Household Survey 2010-2011. Abuja, Nigeria: National Bureau of Statistics (Nigeria)
- National Bureau of Statistics (Nigeria). Nigeria General Household Survey 2012-2013. Washington DC, United States of America: World Bank
- National Bureau of Statistics (Nigeria). Nigeria Living Standards Survey 2008-2010. Abuja, Nigeria: National Bureau of Statistics (Nigeria)
- National Bureau of Statistics (Nigeria). Nigeria National Nutrition and Health Survey 2015. v1.0
- National Malaria Control Program Insecticide Treated Nets Data, Personal Communication with the World Health Organization 2019 as it appears in Malaria Atlas Project. Malaria Atlas Project Interventions Database
- National Population Commission of Nigeria, United Nations Children's Fund (UNICEF). Nigeria Report of Livebirths, Deaths and Stillbirths 1994-2007. Abuja, Nigeria: National Population Commission of Nigeria, 2008
- National Population Commission of Nigeria. Nigeria Population and Housing Census 1991
- National Population Commission of Nigeria. Nigeria Population and Housing Census 2006. Abuja, Nigeria: National Population Commission of Nigeria
- National Population Commission of Nigeria. Nigeria Report on Children, Adolescents and Youth. Abuja, Nigeria: National Population Commission of Nigeria
- National Population Commission of Nigeria. Nigeria Sentinel Survey of The National Population Program 1994
- National Primary Health Care Development Agency (NPHCDA) (Nigeria). Nigeria National Immunization Coverage Survey 2010
- National School of Public Health (Greece), World Health Organization (WHO). Greece WHO Multi-country Survey Study on Health and Health System Responsiveness 2000-2001
- Ngele S.O, Onwu F.K. Measurements of ambient air fine and coarse particulate matter in ten south-east Nigerian cities . Res J Chem Sci. 2015; 5(1): 71-7 as it appears in Shaddick G, Thomas ML. Particulate Matter 2.5 and 10 Surface Monitor Station Expanded Database 2008-2017. [Unpublished]
- Nigeria - Nutritional Status of Women and Children in Nigeria as it appears in World Health Organization (WHO). WHO Global Database on Anemia, Nutrition Landscape Information System. Geneva, Switzerland: World Health Organization (WHO)
- Nigeria Centre for Disease Control. An Update of Meningitis Outbreak in Nigeria for Week 21, June 2017. Nigeria: Nigeria Centre for Disease Control, 2017

- Nigeria Centre for Disease Control. An Update of Meningitis Outbreak in Nigeria for Week 21, June 2019. Nigeria: Nigeria Centre for Disease Control, 2019
- Nigeria Centre for Disease Control. Nigeria Weekly Epidemiological Report 2018 December Week 52. 2019
- Nigeria Centre for Disease Control. Yellow Fever Outbreak in Nigeria Situation Report: Epi Week 1, January 2nd, 2018. Nigeria: Nigeria Centre for Disease Control, 2018
- Nigeria Centre for Disease Control. Yellow Fever Situation Report in Nigeria: Epi Week 9, February 25 - March 3 2019. Nigeria: Nigeria Centre for Disease Control, 2019
- Nigeria Core Welfare Indicators Questionnaire Survey - 8 States 2002 as it appears in World Health Organization (WHO). WHO Household Energy Database 1974-2008. Geneva, Switzerland: World Health Organization (WHO), 2010
- Nigeria Demographic and Health Survey 1999 as it appears in World Health Organization (WHO). WHO Global Database on Child Growth and Malnutrition. Geneva, Switzerland: World Health Organization (WHO)
- Nigeria Demographic and Health Survey 2003 as it appears in Department for International Development (DFiD) (United Kingdom), National Population Commission of Nigeria, ORC Macro, United Nations Children's Fund (UNICEF), United Nations Population Fund (UNFPA). Nigeria Demographic and Health Survey 2003. Fairfax, United States of America: ICF International
- Nigeria Demographic and Health Survey 2003 as it appears in Malaria Atlas Project. Malaria Atlas Project Interventions Database
- Nigeria Demographic and Health Survey 2008 as it appears in Malaria Atlas Project. Malaria Atlas Project Interventions Database
- Nigeria Demographic and Health Survey 2013 as it appears in Malaria Atlas Project. Malaria Atlas Project Interventions Database
- Nigeria Malaria Indicator Survey 2010 as it appears in Malaria Atlas Project. Malaria Atlas Project Interventions Database
- Nigeria Malaria Indicator Survey 2010 as it appears in Malaria Atlas Project. Malaria Atlas Project Plasmodium Falciparum Parasite Rate Database. Oxford, United Kingdom: Malaria Atlas Project
- Nigeria Malaria Indicator Survey 2015 as it appears in Malaria Atlas Project. Malaria Atlas Project Plasmodium Falciparum Parasite Rate Database. Oxford, United Kingdom: Malaria Atlas Project
- Nigeria Multiple Indicator Cluster Survey 2007 as it appears in Malaria Atlas Project. Malaria Atlas Project Interventions Database
- Nigeria Multiple Indicator Cluster Survey 2011 as it appears in Malaria Atlas Project. Malaria Atlas Project Interventions Database
- Nigeria National Consumer Survey 1992-1993 as it appears in World Health Organization (WHO). WHO Household Energy Database 1974-2008. Geneva, Switzerland: World Health Organization (WHO), 2010
- Nigeria National Micronutrient Survey 1993 as it appears in World Health Organization (WHO). WHO Global Database on Anemia, Nutrition Landscape Information System. Geneva, Switzerland: World Health Organization (WHO)
- Nigeria National Micronutrient Survey 1993 as it appears in World Health Organization (WHO). WHO Global Database on Child Growth and Malnutrition - Historical. Geneva, Switzerland: World Health Organization (WHO)

- Nigeria National Micronutrient Survey 1993 as it appears in World Health Organization (WHO). WHO Global Database on Child Growth and Malnutrition. Geneva, Switzerland: World Health Organization (WHO)
- Nigeria National Micronutrient Survey 1993 as it appears in World Health Organization (WHO). WHO Global Database on Iodine Deficiency. Geneva, Switzerland: World Health Organization (WHO)
- Nigeria National Micronutrient Survey 1993 as it appears in World Health Organization (WHO). WHO Global Database on Vitamin A Deficiency. Geneva, Switzerland: World Health Organization (WHO)
- Nigeria Plasmodium Falciparum Parasite Rate Data 2007-2008, Personal Communication with P.M. Graves, F.O. Richards, P.M. Emerson, and E. Emukah, The Carter Center 2009 as it appears in Malaria Atlas Project. Malaria Atlas Project Plasmodium Falciparum Parasite Rate Database. Oxford, United Kingdom: Malaria Atlas Project
- Nigeria Plasmodium Falciparum Parasite Rate Data, Personal Communication with S. Awolola 2009 as it appears in Malaria Atlas Project. Malaria Atlas Project Plasmodium Falciparum Parasite Rate Database. Oxford, United Kingdom: Malaria Atlas Project
- Nigeria Population and Housing Census 1963 as it appears in United Nations Statistics Division (UNSD). United Nations Demographic Yearbook. New York City, United States: United Nations Statistics Division (UNSD)
- Nigeria Population and Housing Census 1991 as it appears in United Nations Statistics Division (UNSD). United Nations Demographic Yearbook. New York City, United States: United Nations Statistics Division (UNSD)
- Nigeria Population and Housing Census 2006 as it appears in United Nations Statistics Division (UNSD). United Nations Demographic Yearbook. New York City, United States: United Nations Statistics Division (UNSD)
- Nigeria World Poll 2005-2006 as it appears in Gallup. Gallup Analytics - World Poll Negative Experience Index. Washington, D.C., United States: Gallup, 2019
- Nigeria World Poll 2007 as it appears in Gallup. Gallup Analytics - World Poll Negative Experience Index. Washington, D.C., United States: Gallup, 2019
- Nigeria World Poll 2008 as it appears in Gallup. Gallup Analytics - World Poll Negative Experience Index. Washington, D.C., United States: Gallup, 2019
- Nigeria World Poll 2009 as it appears in Gallup. Gallup Analytics - World Poll Negative Experience Index. Washington, D.C., United States: Gallup, 2019
- Nigeria World Poll 2010 as it appears in Gallup. Gallup Analytics - World Poll Negative Experience Index. Washington, D.C., United States: Gallup, 2019
- Nigeria World Poll 2011 as it appears in Gallup. Gallup Analytics - World Poll Negative Experience Index. Washington, D.C., United States: Gallup, 2019
- Nigeria World Poll 2012 as it appears in Gallup. Gallup Analytics - World Poll Negative Experience Index. Washington, D.C., United States: Gallup, 2019
- Nigeria World Poll 2013 as it appears in Gallup. Gallup Analytics - World Poll Negative Experience Index. Washington, D.C., United States: Gallup, 2019
- Nigeria World Poll 2014 as it appears in Gallup. Gallup Analytics - World Poll Negative Experience Index. Washington, D.C., United States: Gallup, 2019
- Nigeria World Poll 2015 as it appears in Gallup. Gallup Analytics - World Poll Negative Experience Index. Washington, D.C., United States: Gallup, 2019
- Nigeria World Poll 2016 as it appears in Gallup. Gallup Analytics - World Poll Negative Experience Index. Washington, D.C., United States: Gallup, 2019

- Nigeria World Poll 2017 as it appears in Gallup. Gallup Analytics - World Poll Negative Experience Index. Washington, D.C., United States: Gallup, 2019
- Nigeria World Poll 2018 as it appears in Gallup. Gallup Analytics - World Poll Negative Experience Index. Washington, D.C., United States: Gallup, 2019
- Nriagu J, Afeiche M, Linder A, Arowolo T, Ana G, Sridhar MKC, Oloruntoba EO, Obi E, Ebenebe JC, Orisakwe OE, Adesina A. Lead poisoning associated with malaria in children of urban areas of Nigeria. Int J Hyg Environ Health. 2008; 211(5-6): 591-605
- Nriagu J, Oleru NT, Cudjoe C, Chine A. Lead poisoning of children in Africa, III. Kaduna, Nigeria. Sci Total Environ. 1997; 197(1-3): 13-9
- Nwagha UI, Nwachukwu D, Dim C, Ibekwe PC, Onyebuchi A. Maternal mortality trend in South East Nigeria: less than a decade to the millennium developmental goals. J Womens Health (Larchmt). 2010; 19(2): 323-7
- Nwawolo CC. The WHO Ear and Hearing Disorders Survey Protocol: Practical Challenges in its Use in a Developing Country (Nigeria). Presented at: Informal Consultation on Epidemiology of Deafness and Hearing Impairment in Developing Countries and Update of the WHO Protocol; 2003; Geneva, Switzerland
- Nwosu CM, Njeze GE, Opara C, Nwajuaku C, Chukwurah CK. Central nervous system infections in the rainforest zone of Nigeria. East Afr Med J. 2001; 78(2): 97-101
- Nyenwe EA, Odia OJ, Ihekwaba AE, Ojule A, Babatunde S. Type 2 diabetes in adult Nigerians: a study of its prevalence and risk factors in Port Harcourt, Nigeria. Diabetes Res Clin Pract. 2003; 62(3): 177-85
- Oak Ridge National Laboratory, University of Southampton (United Kingdom), Vaccination Tracking System (VTS) (Nigeria). Nigeria Geospatial Referenced Population Estimates. Seattle, United States of America: Geographic, Population, and Demographic Data (GeoPoDe)
- Obi CL, Coker AO, Epoke J, Ndip RN. Enteric bacterial pathogens in stools of residents of urban and rural regions in Nigeria: a comparison of patients with and without diarrhoea and controls without diarrhoea. J Diarrhoeal Dis Res. 1997; 15(4): 241¬ñ7
- Obi E, Orisakwe OE, Okafor C, Igwebe A, Ebenebe J, Afonne OJ, Ifediata F, Nils B, Nriagu J. Towards prenatal biomonitoring in eastern Nigeria: assessing lead levels and anthropometric parameters of newborns. J UOEH. 2014; 36(3): 159-70
- Obiako MN. Profound childhood deafness in Nigeria: a three year survey. Ear Hear. 1987; 8(2): 74-7
- Obunge OK, Brabin L, Dollimore N, Kemp J, Ikokwu-Wonodi C, Babatunde S, White S, Briggs ND, Hart CA. A flowchart for managing sexually transmitted infections among Nigerian adolescent females. Bull World Health Organ. 2001; 79(4): 301-5
- Odafe S, Idoko O, Badru T, Aiyenigba B, Suzuki C, Khamofu H, Onyekwena O, Okechukwu E, Torpey K, Chabikuli ON. Patients' demographic and clinical characteristics and level of care associated with lost to follow-up and mortality in adult patients on first-line ART in Nigerian hospitals. J Int AIDS Soc. 2012; 15(2): 17424
- Odhiambo JA, Williams HC, Clayton TO, Robertson CF, Asher MI, ISAAC Phase Three Study Group. Global variations in prevalence of eczema symptoms in children from ISAAC Phase Three. J Allergy Clin Immunol. 2009; 124(6): 1251-1258
- Odili AN, Abatta EO. Blood pressure indices, life-style factors and anthropometric correlates of casual blood glucose in a rural Nigerian community. Ann Afr Med. 2015; 14(1): 39¬ñ45

- Odugbo OP, Mpyet CD, Chiroma MR, Aboje AO. Cataract blindness, surgical coverage, outcome, and barriers to uptake of cataract services in Plateau State, Nigeria. Middle East Afr J Ophthalmol. 2012; 19(3): 282-8
- Odum CU, Akinkugbe A. The causes of maternal deaths in eclampsia in Lagos, Nigeria. West Afr J Med. 1991; 10(1): 371-6
- Ofoezie IE, Adeniyi IF. The prevalence and endemicity of dracunculiasis in Akowide village, Oyo State, Nigeria. Ann Trop Med Parasitol. 1990; 84(2): 163-169
- Ogah OS, Madukwe OO, Chukwuonye II, Onyeonoro UU, Ukegbu AU, Akhimien MO, Onwubere BJC, Okpechi IG. Prevalence and determinants of hypertension in Abia State Nigeria: results from the Abia State Non-Communicable Diseases and Cardiovascular Risk Factors Survey. Ethn Dis. 2013; 23(2): 161¬ñ7
- Ogunlesi TA, Dedeke IOF, Adekanmbi AF, Fetuga MB, Ogunfowora OB. The incidence and outcome of bilirubin encephalopathy in Nigeria: a bi-centre study. Niger J Med. 2007; 16(4): 354-9
- Ogunlesi TA, Okeniyi JA, Owa JA, Oyedeji GA. Neonatal tetanus at the close of the 20th century in Nigeria. Trop Doct. 2007; 37(3): 165-7
- Ogunniyi A, Hall KS, Gureje O, Baiyewu O, Gao S, Unverzagt FW, Smith-Gamble V, Evans RE, Dickens J, Musick BS, Hendrie HC. Risk factors for incident Alzheimers disease in African Americans and Yoruba. Metab Brain Dis. 2006; 21(23): 23540
- Ogunniyi A, Lane KA, Baiyewu O, Gao S, Gureje O, Unverzagt FW, Murrell JR, Smith-Gamble V, Hall KS, Hendrie HC. Hypertension and incident dementia in community-dwelling elderly Yoruba Nigerians. Acta Neurol Scand. 2011; 124(6): 396¬ñ402
- Ogunrin OA, Unuigbe EI. Tetanus: an analysis of the prognosticating factors of cases seen in a tertiary hospital in a developing African country between 1990 and 2000. Trop Doct. 2004; 34(4): 240-1
- Ojini FI, Danesi MA. Mortality of tetanus at the Lagos University Teaching Hospital, Nigeria. Trop Doct. 2005; 35(3): 178-81
- Ojuawo A, Joiner KT. Childhood epilepsy in Ilorin, Nigeria. East Afr Med J. 1997; 74(2): 72-75
- Ojurongbe O, Adegbayi AM, Bolaji OS, Akindele AA, Adefioye OA, Adeyeba OA. Asymptomatic falciparum malaria and intestinal helminths co-infection among school children in Osogbo, Nigeria. J Res Med Sci. 2011; 16: 680-6 as it appears in Malaria Atlas Project. Malaria Atlas Project Plasmodium Falciparum Parasite Rate Database. Oxford, United Kingdom: Malaria Atlas Project
- Ojurongbe O, Akinbo JA, Ogiogwa IJ, Bolaji OS, Adeyeba OA. Lymphatic filariasis in a rural community in Nigeria: a challenge ahead. Afr J Med Med Sci. 2010; 179-83 as it appears in London School of Hygiene and Tropical Medicine. Global Atlas of Helminth Infections - Lymphatic Filariasis. London, United Kingdom: London School of Hygiene and Tropical Medicine
- Okafor UH, Ahmed S, Unuigbe EI. Screening for risk factors of chronic kidney disease in a community in Niger Delta Nigeria. Ann Afr Med. 2015; 14(3): 137¬ñ42
- Okesina AB, Oparinde DP, Akindoyin KA, Erasmus RT. Prevalence of some risk factors of coronary heart disease in a rural Nigerian population. East Afr Med J. 1999; 76(4): 212-6
- Okonkwo P, Sagay AS, Agaba PA, Yohanna S, Agbaji OO, Imade GE, Banigbe B, Adeola J, Oyebode TA, Idoko JA, Kanki PJ. Treatment outcomes in a decentralized antiretroviral therapy program: a comparison of two levels of care in north central Nigeria. AIDS Res Treat. 2014; 560623

- Okonofua FE, Feyisetan BJ, Davies-Adetugbo A, Sanusi YO. Influence of socioeconomic factors on the treatment and prevention of malaria in pregnant and non-pregnant adolescent girls in Nigeria. J Trop Med Hyg. 1992; 95(5): 309-15 as it appears in Malaria Atlas Project. Malaria Atlas Project Plasmodium Falciparum Parasite Rate Database. Oxford, United Kingdom: Malaria Atlas Project
- Okoromah CN, Lesi FE, Egri-Okwaji MT, Iroha E. Clinical and management factors related to outcome in neonatal tetanus. Niger Postgrad Med J. 2003; 10(2): 92-5
- Okoye O, Umeh RE, Ezepue FU. Prevalence of eye diseases among school children in a rural south-eastern Nigerian community. Rural Remote Health. 2013; 13(3): 2357
- Okpechi IG, Chukwuonye II, Tiffin N, Madukwe OO, Onyeonoro UU, Umeizudike TI, Ogah OS. Blood pressure gradients and cardiovascular risk factors in urban and rural populations in Abia State South Eastern Nigeria using the WHO STEPwise approach. PLoS One. 2013; 8(9): e73403
- Okubadejo NU, Ojo OO, Oshinaike OO. Clinical profile of parkinsonism and Parkinson's disease in Lagos, Southwestern Nigeria. BMC Neurol. 2010; 10: 1
- Okunola Y, Ayodele O, Akinwusi P, Gbadegesin B, Oluyombo R. Haemodialysis practice in a resource-limited setting in the tropics. Ghana Med J. 2013; 47(1): 4¬ñ9
- Okusanya BO, Aigere EOS, Abe A, Ibrahim HM, Salawu RA. Maternal deaths: initial report of an on-going monitoring of maternal deaths at the Federal Medical Centre Katsina, Northwest Nigeria. J Matern Fetal Neonatal Med. 2013; 26(9): 885-8
- Okwerekwu FE. Maternal mortality in Nigerian women aged 35 years and above. Asia Oceania J Obstet Gynaecol. 1991; 17(1): 37-44
- Ola BA, Adewuya AO, Ajayi OE, Akintomide AO, Oginni OO, Ologun YA. Relationship between depression and quality of life in Nigerian outpatients with heart failure. J Psychosom Res. 2006; 61(6): 797-800
- Oladapo OO, Salako L, Sodiq O, Shoyinka K, Adedapo K, Falase AO. A prevalence of cardiometabolic risk factors among a rural Yoruba south-western Nigerian population: a population-based survey. Cardiovasc J Afr. 2010; 21(1): 26¬ñ31
- Oladapo OT,¬†Sule-Odu AO,¬†Olatunji AO,¬†Daniel OJ. "Near Miss" obstetric events and maternal deaths in Sagamu, Nigeria: a retrospective study. Reprod Health. 2005; 2(9): 1-9
- Olaleye OD, Omilabu SA, Faseru O, Fagbami AH. Nigeria: a survey for yellow fever virus haemagglutination inhibiting antibody in residents of two communities before and after the epidemics. Virologie. 1988; 39(4): 261-6
- Oluleye TS. Cataract blindness and barriers to cataract surgical intervention in three rural communities of Oyo State, Nigeria. Niger J Med. 2004; 13(2): 156-60
- Olusanya BO. Perinatal outcomes of multiple births in southwest Nigeria. J Health Popul Nutr. 2011; 29(6): 639-47
- Olutayo Alebiosu C, Ayodele OO, Abbas A, Ina Olutoyin A. Chronic renal failure at the Olabisi Onabanjo university teaching hospital, Sagamu, Nigeria. Afr Health Sci. 2006; 6(3): 132¬ñ8
- Oluyombo R, Okunola OO, Olanrewaju TO, Soje MO, Obajolowo OO, Ayorinde MA. Challenges of Hemodialysis in a New Renal Care Center: Call for Sustainability and Improved Outcome. Int J Nephrol Renovascular Dis. 2014; 7: 347¬ñ52
- Omosun YO, Anumudu CI, Adoro S, Odaibo AB, Sodeinde O, Holder AA, Nwagwu M, Nwuba RI. Variation in the relationship between anti-MSP-1(19) antibody response and age in children infected with Plasmodium falciparum during the dry and rainy seasons. Addict Behav Rep. 2005; 95(3): 233-47 as it appears in Malaria Atlas Project. Malaria Atlas Project

Plasmodium Falciparum Parasite Rate Database. Oxford, United Kingdom: Malaria Atlas Project

- Omotade OO, Adeyemo AA, Kayode CM, Falade SL, Ikpeme S. Gene frequencies of ABO and Rh (D) blood group alleles in a healthy infant population in Ibadan, Nigeria. West Afr J Med. 1999; 18(4): 294-7
- Onanuga A, Igbeneghu O, Lamikanra A. A study of the prevalence of diarrhoeagenic Escherichia coli in children from Gwagwalada, Federal Capital Territory, Nigeria. Pan Afr Med J. 2014; 17: 146
- Onwuchekwa AC, Mezie-Okoye MM, Babatunde S. Prevalence of hypertension in Kegbara-Dere, a rural community in the Niger Delta region, Nigeria. Ethn Dis. 2012; 22(3): 340-6
- Onwudiegwu U. The effect of a depressed economy of the utilisation of maternal health services: the Nigerian Experience II. J Obstet Gynaecol. 1997; 17(2): 143-8
- Onyema OA, Cornelius AC, Uchenna ET, Duke OA. PRIMARY POSTPARTUM HAEMORRHAGE IN FEDERAL MEDICAL CENTRE, OWERRI, NIGERIA: A SIX YEAR REVIEW. Niger J Med. 2015; 24(3): 242-5
- Onyiriuka AN. Trends in incidence of delivery of low birth weight infants in Benin City, southern Nigeria. Niger Postgrad Med J. 2006; 13(3): 189-94
- Oparaocha E. The impact of haemoglobin level and concomitant infections on malaria parasitaemia and on-set of fever during malaria attack in Ikwuano local government area of Abia state, Nigeria. Niger J Parasitol. 2003; 24: 25-32 as it appears in Malaria Atlas Project. Malaria Atlas Project Plasmodium Falciparum Parasite Rate Database. Oxford, United Kingdom: Malaria Atlas Project
- Orhue A, Aziken M. Experience with a comprehensive university hospital-based infertility program in Nigeria. Int J Gynaecol Obstet. 2008; 101(1): 11-5
- Oruamabo RS, Igbagiri FP. Neonatal tetanus in Port Harcourt. Afr J Med Med Sci. 1996; 25(3): 265-8
- Osinusi K, Njinyam MN. A new prognostic scoring system in neonatal tetanus. Afr J Med Med Sci. 1997; 26(3-4): 123-5
- Owoade OK, Fawole OG, Olise FS, Ogundele LT, Olaniyi HB, Almeida MS, Ho M-D, Hopke PK. Characterization and source identification of airborne particulate loadings at receptor site-classes of Lagos Mega-City, Nigeria. J Air Waste Manag Assoc. 2013; 63(9): 1026¬ñ35 as it appears in Shaddick G, Thomas ML. Particulate Matter 2.5 and 10 Surface Monitor Station Expanded Database 2008-2017. [Unpublished]
- Owoaje EE, Rotimi CN, Kaufman JS, Tracy J, Cooper RS. Prevalence of adult diabetes in Ibadan, Nigeria. East Afr Med J. 1997; 74(5): 299-302
- Oye-Adeniran BA, Odeyemi KA, Gbadegesin A, Ekanem EE, Osilaja OK, Akin-Adenekan O, Umoh AV. The use of the sisterhood method for estimating maternal mortality ratio in Lagos state, Nigeria. J Obstet Gynaecol. 2011; 31(4): 315-9
- Oziegbe EO, Esan TA. Prevalence and clinical consequences of untreated dental caries using PUFA index in suburban Nigerian school children. Eur Arch Paediatr Dent. 2013; 14(4): 227-31
- Ozoegwu PN, Onwurah AE. Prevalence Of Haemoglobinopathy And Malaria Diseases In The Population Of Old Aguata Division, Anambra State, Nigeria. Biokemistri. 2005; 15(2): 57¬ñ66 as it appears in Malaria Atlas Project. Malaria Atlas Project Plasmodium Falciparum Parasite Rate Database. Oxford, United Kingdom: Malaria Atlas Project
- Pew Research Center. The Future of the Global Muslim Population. Washington, DC, United States: Pew Research Center, 2011

- Plasmodium Falciparum Parasite Rate Data, Personal Communication with Mapping Malaria Risk In Africa 2009 as it appears in Malaria Atlas Project. Malaria Atlas Project Plasmodium Falciparum Parasite Rate Database. Oxford, United Kingdom: Malaria Atlas Project
- Popoola BO, Onyejaka N, Folayan MO. Prevalence of developmental dental hard-tissue anomalies and association with caries and oral hygiene status of children in Southwestern, Nigeria. BMC Oral Health. 2016; 17(1): 8
- Popova S, Lange S, Probst C, Gmel G, Rehm J, Centre for Addiction and Mental Health (Canada). Estimation of national, regional and global prevalence of alcohol use during pregnancy and fetal alcohol syndrome: a systematic review and meta-analysis. Lancet Glob Health. [Forthcoming]
- Porter IH, Boyer SH. Variation of glucose-6-phosphate dehydrogenase in different populations. Lancet. 1964; 2(7367): 1016
- Prevalence estimates based on the African Programme for Onchocerciasis Control Community-directed Treatment with Ivermectin Database and Onchocerciasis Control Programme, as provided by the GBD 2010 Onchocerciasis Expert Group
- Pugh RN, Bell DR, Gilles HM. Malumfashi Endemic Diseases Research Project, XV The potential medical importance of bilharzia in northern Nigeria: a suggested rapid, cheap and effective solution for control of Schistosoma haematobium infection. Ann Trop Med Parasitol. 1980; 74(6): 597-613
- Pullan RL, Smith JL, Jasrasaria R, Brooker SJ. Global numbers of infection and disease burden of soil transmitted helminth infections in 2010 [Unpublished data]. Parasit Vectors. 2014; 7(37). [Unpublished data as provided by the Global Burden of Disease 2010 soil transmitted helminths expert group]
- Rabiu MM, Muhammed N. Rapid Assessment of cataract surgical services in Birnin-Kebbi local government area of Kebbi State, Nigeria. Ophthalmic Epidemiol. 2008; 15(6): 359-65
- Rabiu MM, Ozemela CP, Apiafi DI. Gathering Baseline Data for a Rural Cataract Surgical Outreach Service in Igabi District, Nigeria. Niger J Ophthalmol. 2007; 15(2): 64-8
- Rabiu MM. Cataract blindness and barriers to uptake of cataract surgery in a rural community of northern Nigeria. Br J Ophthalmol. 2001; 85(7): 776-80
- Radin JM, Katz MA, Tempia S, Talla Nzussouo N, Davis R, Duque J, Adedeji A, Adjabeng MJ, Ampofo WK, Ayele W, Bakamutumaho B, Barakat A, Cohen AL, Cohen C, Dalhatu IT, Daouda C, Dueger E, Francisco M, Heraud J-M, Jima D, Kabanda A, Kadjo H, Kandeel A, Bi Shamamba SK, Kasolo F, Kronmann KC, Mazaba Liwewe ML, Lutwama JJ, Matonya M, Mmbaga V, Mott JA, Muhimpundu MA, Muthoka P, Njuguna H, Randrianasolo L, Refaey S, Sanders C, Talaat M, Theo A, Valente F, Venter M, Woodfill C, Bresee J, Moen A, Widdowson M-A. Influenza surveillance in 15 countries in Africa, 2006-2010. J Infect Dis. 2012; S14¬ñ21
- Sabir A, Ohwovoriole A, Isezuo S, Fasanmade O, Abubakar S, Iwuala S. Type 2 diabetes mellitus and its risk factors among the rural Fulanis of Northern Nigeria. Ann Afr Med. 2013; 12(4): 217-22
- Sabir AA, Isezuo SA, Ohwovoriole AE, Fasanmade OA, Abubakar SA, Iwuala S, Umar MT. Rural-urban difference in plasma lipid levels and prevalence of dyslipidemia in Hausa-Fulani of north-western Nigeria. Ethn Dis. 2013; 23(3): 374¬ñ8
- Salako LA, Ajayi FO, Sowunmi A, Walker O. Malaria in Nigeria: a revisit. Ann Trop Med Parasitol. 1990; 84(5): 435-45 as it appears in Malaria Atlas Project. Malaria Atlas Project Plasmodium Falciparum Parasite Rate Database. Oxford, United Kingdom: Malaria Atlas Project

- Shaddick G, Thomas ML. Particulate Matter 2.5 and 10 Surface Monitor Station Expanded Database 2008-2017. [Unpublished]
- Singh R, Godson II, Singh S, Singh RB, Isyaku NT, Ebere UV. High prevalence of asymptomatic malaria in apparently healthy schoolchildren in Aliero, Kebbi state, Nigeria. J Vector Borne Dis. 2014; 51(2): 128-32 as it appears in Malaria Atlas Project. Malaria Atlas Project Plasmodium Falciparum Parasite Rate Database. Oxford, United Kingdom: Malaria Atlas Project
- Singh S, Chukwunyere DN, Omembelede J, Onankpa B. Foetal congenital anomalies: An experience from a tertiary health institution in north-west Nigeria (2011-2013). Niger Postgrad Med J. 2015; 22(3): 174-8
- Sloan N, Durocher J, Aldrich T, Blum J, Winikoff B. What measured blood loss tells us about postpartum bleeding: a systematic review. BJOG. 2010; 117(7): 788-800
- Snow RW, Sartorius B, Kyalo D, Maina J, Amratia P, Mundia CW, Bejon P, Noor AM. The prevalence of Plasmodium falciparum in sub-Saharan Africa since 1900. Nature. 2017; 550(7677): 515¬ñ8 as it appears in Malaria Atlas Project. Malaria Atlas Project Plasmodium Falciparum Parasite Rate Database. Oxford, United Kingdom: Malaria Atlas Project
- Sofola OO, Folayan MO, Oginni AB. Changes in the prevalence of dental caries in primary school children in Lagos State, Nigeria. Niger J Clin Pract. 2014; 17(2): 127-33
- Sowunmi A. Body temperature and malaria parasitaemia in rural African children. East Afr Med J. 1995; 72(7): 427-30 as it appears in Malaria Atlas Project. Malaria Atlas Project Plasmodium Falciparum Parasite Rate Database. Oxford, United Kingdom: Malaria Atlas Project
- Sunday-Adeoye¬† I, Okonta¬† PI, Egwuatu¬† VE. Congenital malformations in singleton and twin births in rural Nigeria. Niger Postgrad Med J. 2007; 14(4): 277-80
- Taiwo JO, Omokhodion F. Pattern of tooth loss in an elderly population from Ibadan, Nigeria. Gerodontology. 2006; 23(2): 117-22
- Targema CN, Onwuliri COE, Mwansat GS. Bancroftian filariasis in Ushongo Local Government Area of Benue State,¬†Nigeria: Clinical, parasitological and serological studies in an endemic¬†community. Int J Environ Health Hum Develop. 2003; 1-4 as it appears in London School of Hygiene and Tropical Medicine. Global Atlas of Helminth Infections - Lymphatic Filariasis. London, United Kingdom: London School of Hygiene and Tropical Medicine
- Tayo BO, Luke A, McKenzie CA, Kramer H, Cao G, Durazo-Arvizu R, Forrester T, Adeyemo AA, Cooper RS. Pattern of sodium and potassium excretion and blood pressure in the African diaspora. J Hum Hypertens. 2012; 26(5): 315-24 as it appears in Tayo BO, Luke A, McKenzie CA, Kramer H, Cao G, Durazo-Arvizu R, Forrester T, Adeyemo AA, Cooper RS. Pattern of sodium and potassium excretion and blood pressure in the African diaspora. J Hum Hypertens. 2012; 26(5): 315-24
- Tebeu PM, Fomulu JN, Khaddaj S, de Bernis L, Delvaux T, Rochat CH. Risk factors for obstetric fistula: a clinical review. Int Urogynecol J. 2012; 23(4): 387-94
- Tobin AO, Ajayi IO. Common oral conditions and correlates: an oral health survey in Kwara State Nigeria. BMC Res Notes. 2017; 10(1): 568
- Tomori O. Yellow fever in Africa: public health impact and prospects for control in the 21st century. Biomedica. 2002; 22(2): 178-210
- U.S. Department of Agriculture (USDA). USDA Global Tobacco Database 1960-2005. Washington D.C. , United States: U.S. Department of Agriculture (USDA)
- Udo S, Chukwu J, Obasanya J. Leprosy situation in Nigeria. Lepr Rev. 2013; 84(3): 229-37

- Udoh EE, Oyo-Ita AE, Odey FA, Eyong KI, Oringanje CM, Oduwole OA, Okebe JU, Esu EB, Meremikwu MM, Asindi AA. Malariometric Indices among Nigerian Children in a Rural Setting. Malar Res Treat. 2013; 2013: 716805 as it appears in Malaria Atlas Project. Malaria Atlas Project Plasmodium Falciparum Parasite Rate Database. Oxford, United Kingdom: Malaria Atlas Project
- Udoidung NI, Braide EI, Opara KN, Atting IA, Adie HA. Current status of bancroftian filariasis in rural communities of the lower cross river basin, Nigeria: Parasitological and clinical aspects. J Public Health. 2008; 16(6): 383-8
- Udonsi JK. Bancroftian filariasis in the Igwun basin, Nigeria: an epidemiological, parasitological, and clinical study in relation to the transmission dynamics. Folia Parasitol (Praha). 1988; 35(2): 147-55
- Udonsi JK. The status of human filariasis in relation to clinical signs in endemic areas of the Niger Delta. Ann Trop Med Parasitol. 1986; 80(4): 425-32 as it appears in London School of Hygiene and Tropical Medicine. Global Atlas of Helminth Infections - Lymphatic Filariasis. London, United Kingdom: London School of Hygiene and Tropical Medicine
- Ufomadu GO, Nwoke BE, Akoh JI, Sato Y, Ekejindu GO, Uchida A, Shiwaku K, Tumbau M, Ugomo KK. The occurrence of loiasis, mansonellosis and wuchereriasis in the Jarawa River Valley, central Nigeria. Acta Trop. 1990; 48(2): 137-47
- Ugwu EO, Onwuka CI, Okezie OA. Pattern and outcome of infertility in Enugu: the need to improve diagnostic facilities and approaches to management. Niger J Med. 2012; 21(2): 180-4
- Ugwu RO, Okoro PE. Pattern, outcome and challenges of neonatal surgical cases in a tertiary teaching hospital. Afr J Paediatr Surg. 2013; 10(3): 226-30
- Ugwuja EI, Ogbu IS, Umeaku EA, Otuu FC. Blood lead levels in children attending a tertiary teaching hospital in Enugu, south-eastern Nigeria. Paediatr Int Child Health. 2014; 34(3): 216-9
- Ulasi II, Ijoma CK. The enormity of chronic kidney disease in Nigeria: the situation in a teaching hospital in South-East Nigeria. J Trop Med. 2010; 2010: 501957
- Umar MU, Obindo JT, Omigbodun OO. Prevalence and Correlates of ADHD Among Adolescent Students in Nigeria. J Atten Disord. 2015
- Umeora OUJ, Ande ABA, Onuh SO, Okubor PO, Mbazor JO. Incidence and risk factors for preterm delivery in a tertiary health institution in Nigeria. J Obstet Gynaecol. 2004; 24(8): 895-6
- Uneke CJ, Ogbu O, Inyama PU, Anyanwu GI, Uneke GI. Malaria infection in HIV-seropositive and HIV-seronegative individuals in Jos-Nigeria. J Vector Borne Dis. 2005; 42(4): 151¬ñ4 as it appears in Malaria Atlas Project. Malaria Atlas Project Plasmodium Falciparum Parasite Rate Database. Oxford, United Kingdom: Malaria Atlas Project
- United Nations Children's Fund (UNICEF), National Bureau of Statistics (Nigeria). Nigeria Multiple Indicator Cluster Survey 2007. New York, United States: United Nations Children's Fund (UNICEF)
- United Nations Children's Fund (UNICEF), World Health Organization (WHO). WHO and UNICEF Reported Disease Incidence Time Series. Geneva, Switzerland: World Health Organization (WHO)
- United Nations Children's Fund (UNICEF), World Health Organization (WHO). WHO and UNICEF Reported Official Target Population, Number of Doses Administered and Official Coverage 1966-2018. Geneva, Switzerland: World Health Organization (WHO), 2019

- United Nations Children's Fund (UNICEF). UNICEF Maternal and Newborn Health Coverage Database as of November 2019. New York, United States of America: United Nations Children's Fund (UNICEF), 2019
- United Nations Children's Fund (UNICEF). UNICEF State of the World's Children 2017. New York, United States: United Nations Children's Fund (UNICEF), 2017
- United Nations Environment Programme. Leaded Petrol Phase-out: Global Status, March 2018. Nairobi, Kenya: United Nations Environment Programme
- United Nations Office on Drugs and Crime (UNODC). World Drug Report 2012. Vienna, Austria: United Nations Office on Drugs and Crime (UNODC), 2012
- United Nations Population Division. Abortion Policies: A Global Review. New York, United States of America: United Nations (UN), 2002
- United Nations Population Division. World Abortion Policies 2007. New York, United States of America: United Nations (UN), 2007
- United Nations Population Division. World Abortion Policies 2013. New York, United States of America: United Nations (UN), 2013
- United Nations Statistics Division (UNSD). National Accounts Main Aggregates Database. New York City, United States of America: United Nations Statistics Division (UNSD)
- United Nations Statistics Division (UNSD). United Nations Demographic Yearbook 2016. New York, United States of America: United Nations (UN), 2017
- University of Ibadan (Nigeria), World Health Organization (WHO). Nigeria WHO Multi-country Survey Study on Health and Health System Responsiveness 2000-2001
- Uttah EC, Simonsen PE, Pedersen EM, Udonsi JK. Bancroftian filariasis in the lower Imo River Basin, Nigeria. Afr J Appl Zool Environ Biol. 2004; 65-75 as it appears in London School of Hygiene and Tropical Medicine. Global Atlas of Helminth Infections - Lymphatic Filariasis. London, United Kingdom: London School of Hygiene and Tropical Medicine
- Uzoewulu GN, Lawson L, Nnanna IS, Rastogi N, Goyal M. Genetic Diversity of Mycobacterium Tuberculosis Complex Strains Isolated from Patients with Pulmonary Tuberculosis in Anambra State, Nigeria. Int J Mycobacteriol. 2016; 5(1): 74¬ñ9
- Vogel JP, Lee ACC, Souza JP. Maternal morbidity and preterm birth in 22 low- and middle-income countries: a secondary analysis of the WHO Global Survey dataset. BMC Pregnancy Childbirth. 2014; 56
- Wall LL, Karshima JA, Kirschner C, Arrowsmith SD. The obstetric vesicovaginal fistula: Characteristics of 899 patients from Jos, Nigeria. Am J Obstet Gynecol. 2004; 190(4): 1011¬ñ9
- Wall LL, Karshima JA, Kirschner C, Arrowsmith SD. The obstetric vesicovaginal fistula: Characteristics of 899 patients from Jos, Nigeria. Am J Obstet Gynecol. 2004; 190(4): 1011¬ñ9 as it appears in Zheng A, Anderson F. Obstetric fistula in low-income countries. Int J Gynaecol Obstet. 2009; 104: 85-9
- Weatherall D. Sickle Cell and Thalassemias Prevalence Data, Personal Correspondence with David Weatherall. [Unpublished]
- Weldegebriel GG, Gasasira A, Harvey P, Masresha B, Goodson JL, Pate MA, Abanida E, Chevez A. Measles resurgence following a nationwide measles vaccination campaign in Nigeria, 2005-2008. J Infect Dis. 2011; 204(Suppl 1): S226-231
- WHO Department of Communicable Disease Surveillance and Response. WHO Report on Global Surveillance of Epidemic-prone Infectious Diseases 2000
- World Bank. World Development Indicators - Gross Domestic Product (GDP). Washington DC, United States of America: World Bank

- World Bank. World Development Indicators - Hospital Beds (per 1,000 People). Washington DC, United States: World Bank
- World Bank. World Development Indicators - Vitamin A Supplementation Coverage Rate. Washington DC, United States of America: World Bank
- World Energy Council. Survey of Energy Resources 2010. London, United Kingdom: World Energy Council, 2010
- World Health Organization (WHO). Dracunculiasis Eradication: Global Surveillance Summary, 2001. Wkly Epidemiol Rec. 2002; 77(18): 141-152
- World Health Organization (WHO). Dracunculiasis Eradication: Global Surveillance Summary, 2002. Wkly Epidemiol Rec. 2003; 78(18): 145-156
- World Health Organization (WHO). Dracunculiasis Eradication: Global Surveillance Summary, 2004. Wkly Epidemiol Rec. 2005; 80(19): 165-175
- World Health Organization (WHO). Dracunculiasis Eradication: Global Surveillance Summary, 2006. Wkly Epidemiol Rec. 2007; 82(16): 133-139
- World Health Organization (WHO). Dracunculiasis: Global Surveillance Summary, 1996. Wkly Epidemiol Rec. 1997; 19(72): 133-140
- World Health Organization (WHO). Dracunculiasis: Global Surveillance Summary, 2000. Wkly Epidemiol Rec. 2001; 76(18): 133-140
- World Health Organization (WHO). Global leprosy situation, 2004. Wkly Epidemiol Rec. 2005: 80(13): 118-24
- World Health Organization (WHO). Global leprosy situation, 2005. Wkly Epidemiol Rec. 2005: 80(34): 289-95.
- World Health Organization (WHO). Global leprosy situation, 2006. Wkly Epidemiol Rec. 2006: 81(32): 309-16.
- World Health Organization (WHO). Global leprosy situation, 2007. Wkly Epidemiol Rec. 2007: 82(25): 225-32
- World Health Organization (WHO). Global leprosy situation, 2009. Wkly Epidemiol Rec. 2009: 84(33): 333-40.
- World Health Organization (WHO). Global leprosy situation, 2010. Wkly Epidemiol Rec. 2010: 85(35): 337-48.
- World Health Organization (WHO). Global leprosy situation, beginning of 2008. Wkly Epidemiol Rec. 2008: 83(33): 293-300.
- World Health Organization (WHO). Global leprosy update, 2013; reducing disease burden. Wkly Epidemiol Rec. 2014; 89(36): 389-400.
- World Health Organization (WHO). Global leprosy update, 2014: need for early case detection. Wkly Epidemiol Rec. 2015; 90(36): 461¬ñ74.
- World Health Organization (WHO). Global leprosy update, 2015: time for action, accountability and inclusion. Wkly Epidemiol Rec. 2016; 92(35): 405¬ñ20.
- World Health Organization (WHO). Global leprosy: update on the 2012 situation. Wkly Epidemiol Rec. 2013: 88(35): 365-79.
- World Health Organization (WHO). Global Status Report on Road Safety 2009. Geneva, Switzerland: World Health Organization (WHO), 2009
- World Health Organization (WHO). Global Status Report on Road Safety 2013. Geneva, Switzerland: World Health Organization (WHO), 2013
- World Health Organization (WHO). Global Status Report on Road Safety 2015. Geneva, Switzerland: World Health Organization (WHO), 2015

- World Health Organization (WHO). Global Status Report on Road Safety 2018. Geneva, Switzerland: World Health Organization (WHO), 2018
- World Health Organization (WHO). Human African trypanosomiasis (sleeping sickness): epidemiological update. Wkly Epidemiol Rec. 2006; 81(8): 71-80
- World Health Organization (WHO). Leprosy update, 2011. Wkly Epidemiol Rec. 2011: 86(36): 389-99.
- World Health Organization (WHO). Nigeria National Immunization Coverage Survey 2003
- World Health Organization (WHO). Nigeria National Immunization Coverage Survey 2006
- World Health Organization (WHO). Nigeria WHO Leishmaniasis Country Profile 1994-2008. Geneva, Switzerland: World Health Organization (WHO)
- World Health Organization (WHO). Protection At Birth (PAB) Against Tetanus with Tetanus Toxoid 1980-2017. Geneva, Switzerland: World Health Organization (WHO)
- World Health Organization (WHO). WHO Disease Outbreak News: Yellow Fever - Nigeria: 22 December 2017. Geneva, Switzerland: World Health Organization (WHO), 2018
- World Health Organization (WHO). WHO Distribution of Measles Cases by Country and by Month 2011-2020
- World Health Organization (WHO). WHO Global Database on Child Growth and Malnutrition - Historical. Geneva, Switzerland: World Health Organization (WHO)
- World Health Organization (WHO). WHO Global Database on Child Growth and Malnutrition. Geneva, Switzerland: World Health Organization (WHO)
- World Health Organization (WHO). WHO Global Database on Vitamin A Deficiency. Geneva, Switzerland: World Health Organization (WHO)
- World Health Organization (WHO). WHO Global Health Expenditure Database - National Health Accounts Indicators. Geneva, Switzerland: World Health Organization (WHO)
- World Health Organization (WHO). WHO Global Health Observatory - Cholera: Number of Reported Cases by Country. Geneva, Switzerland: World Health Organization (WHO)
- World Health Organization (WHO). WHO Global Health Observatory - Cholera: Number of Reported Deaths by Country. Geneva, Switzerland: World Health Organization (WHO)
- World Health Organization (WHO). WHO Global Health Observatory - Meningiccocoal Meningitis: Number of Suspected Deaths by Country. Geneva, Switzerland: World Health Organization (WHO)
- World Health Organization (WHO). WHO Global Health Observatory - Number of New Reported Cases (T.b. Gambiense), Data by Country. Geneva, Switzerland: World Health Organization (WHO)
- World Health Organization (WHO). WHO Global Health Observatory - Population Living in Trachoma Endemic Areas. Geneva, Switzerland: World Health Organization (WHO)
- World Health Organization (WHO). WHO Global Health Observatory - Recorded Alcohol Per Capita Consumption 1960-1979. Geneva, Switzerland: World Health Organization (WHO)
- World Health Organization (WHO). WHO Global Health Observatory - Recorded Alcohol Per Capita Consumption 1980-1999. Geneva, Switzerland: World Health Organization (WHO)
- World Health Organization (WHO). WHO Global Health Observatory - Recorded Alcohol Per Capita Consumption 2000-2009 by country. Geneva, Switzerland: World Health Organization (WHO)
- World Health Organization (WHO). WHO Global Health Observatory - Visceral Leishmaniasis: Number of Reported Cases by Country. Geneva, Switzerland: World Health Organization (WHO)

- World Health Organization (WHO). WHO Global Health Observatory (GHO) Data - Hospital Bed Density Data by Country . Geneva, Switzerland: World Health Organization (WHO)
- World Health Organization (WHO). WHO Global Health Observatory Interactive Graph - Number of Cases of Cutaneous Leishmaniasis Reported 2008. Geneva, Switzerland: World Health Organization (WHO)
- World Health Organization (WHO). WHO Global Health Observatory Interactive Graph - Number of Cases of Visceral Leishmaniasis Reported 1998. Geneva, Switzerland: World Health Organization (WHO)
- World Health Organization (WHO). WHO Global Health Observatory Interactive Graph - Number of Cases of Visceral Leishmaniasis Reported 1999. Geneva, Switzerland: World Health Organization (WHO)
- World Health Organization (WHO). WHO Global Health Observatory Interactive Graph - Number of Cases of Visceral Leishmaniasis Reported 2000. Geneva, Switzerland: World Health Organization (WHO)
- World Health Organization (WHO). WHO Global Health Observatory Interactive Graph - Number of Cases of Visceral Leishmaniasis Reported 2001. Geneva, Switzerland: World Health Organization (WHO)
- World Health Organization (WHO). WHO Global Health Observatory Interactive Graph - Number of Cases of Visceral Leishmaniasis Reported 2003. Geneva, Switzerland: World Health Organization (WHO)
- World Health Organization (WHO). WHO Global Health Observatory Interactive Graph - Number of Cases of Visceral Leishmaniasis Reported 2004. Geneva, Switzerland: World Health Organization (WHO)
- World Health Organization (WHO). WHO Global Health Observatory Interactive Graph - Number of Cases of Visceral Leishmaniasis Reported 2005. Geneva, Switzerland: World Health Organization (WHO)
- World Health Organization (WHO). WHO Global Health Observatory Interactive Graph - Number of Cases of Visceral Leishmaniasis Reported 2006. Geneva, Switzerland: World Health Organization (WHO)
- World Health Organization (WHO). WHO Global Health Observatory Interactive Graph - Number of Cases of Visceral Leishmaniasis Reported 2007. Geneva, Switzerland: World Health Organization (WHO)
- World Health Organization (WHO). WHO Global Health Observatory Interactive Graph - Number of Cases of Visceral Leishmaniasis Reported 2008. Geneva, Switzerland: World Health Organization (WHO)
- World Health Organization (WHO). WHO Global Health Observatory Interactive Graph - Number of Cases of Visceral Leishmaniasis Reported 2009. Geneva, Switzerland: World Health Organization (WHO)
- World Health Organization (WHO). WHO Global Health Observatory Interactive Graph - Number of Cases of Visceral Leishmaniasis Reported 2010. Geneva, Switzerland: World Health Organization (WHO)
- World Health Organization (WHO). WHO Global Health Observatory Interactive Graph - Number of Cases of Visceral Leishmaniasis Reported 2011. Geneva, Switzerland: World Health Organization (WHO)
- World Health Organization (WHO). WHO Global Health Observatory Interactive Graph - Number of Cases of Visceral Leishmaniasis Reported 2012. Geneva, Switzerland: World Health Organization (WHO)

- World Health Organization (WHO). WHO Global Health Observatory Interactive Graph - Number of Cases of Visceral Leishmaniasis Reported 2013. Geneva, Switzerland: World Health Organization (WHO)
- World Health Organization (WHO). WHO Global Health Observatory Interactive Graph - Number of Cases of Visceral Leishmaniasis Reported 2014. Geneva, Switzerland: World Health Organization (WHO)
- World Health Organization (WHO). WHO Global Health Observatory Interactive Graph - Number of Cases of Visceral Leishmaniasis Reported 2015. Geneva, Switzerland: World Health Organization (WHO)
- World Health Organization (WHO). WHO Global Project on Anti-Tuberculosis Drug Resistance Surveillance Data 1988-2015
- World Health Organization (WHO). WHO Global Survey on Maternal and Perinatal Health 2004-2008
- World Health Organization (WHO). WHO Household Energy Database 1974-2008. Geneva, Switzerland: World Health Organization (WHO), 2010
- World Health Organization (WHO). WHO PCT Databank - Schistosomiasis. Geneva, Switzerland: World Health Organization (WHO)
- World Health Organization (WHO). WHO PCT Databank - Soil-transmitted Helminthiases. Geneva, Switzerland: World Health Organization (WHO)
- World Health Organization (WHO). WHO Tuberculosis Case Notifications. Geneva, Switzerland: World Health Organization (WHO)
- World Health Organization (WHO). WHO World Mental Health Surveys: Global Perspectives on the Epidemiology of Mental Disorders. Cambridge, United Kingdom: Cambridge University Press, 2008
- World Health Organization (WHO). Global leprosy update, 2016: accelerating reduction of disease burden. Wkly Epidemiol Rec. 2017; 92(35): 501-20
- World Health Organization (WHO). Global leprosy update, 2017: reducing the disease burden due to leprosy. Wkly Epidemiol Rec. 2018; 93(35): 445-56
- World Health Organization (WHO). Monthly Report on Dracunculiasis Cases, January-December 2017. WHO Wkly Epidemiol Rec. 2018; 93(6): 45-60
- World Health Organization Expert Committee on the Control and Surveillance of African Trypanosomiasis. Control and surveillance of human African trypanosomiasis. WHO Technical Report Series 881. Geneva, Switzerland: World Health Organization (WHO), 1995
- World Health Organization Regional Office for Africa (WHO-AFRO). Weekly Bulletins on Outbreaks and Other Emergencies 2018. Brazaville , Congo: World Health Organization Regional Office for Africa (WHO-AFRO), 2018
- World Health Organization Regional Office for Africa (WHO-AFRO). Weekly Bulletins on Outbreaks and Other Emergencies: 6-12 January 2018 to 29 December 2018 - 4 January 2019. Brazaville , Congo: World Health Organization Regional Office for Africa (WHO-AFRO)
- World Health Organization Regional Office for Africa (WHO-AFRO). WHO Africa Weekly Bulletin on Outbreaks and Other Emergencies - Week 1 : 29 December 2018 - 4 January 2019. Brazaville, Congo: World Health Organization Regional Office for Africa (WHO-AFRO), 2019
- World Health Organization. Dracunculiasis: Global Surveillance Summary, 1994. Wkly Epidemiol Rec. 1993; 70(18): 125-131

- World Malaria Report 2013 as it appears in Malaria Atlas Project. Malaria Atlas Project Annual Parasite Incidence Database
- World Malaria Report 2015 as it appears in Malaria Atlas Project. Malaria Atlas Project Annual Parasite Incidence Database
- World Malaria Report 2016 as it appears in Malaria Atlas Project. Malaria Atlas Project Annual Parasite Incidence Database
- World Malaria Report 2017 as it appears in Malaria Atlas Project. Malaria Atlas Project Interventions Database
- Wright EA. Low birthweight in the plateau region of Nigeria. East Afr Med J. 1990; 67(12): 894-9
- Wright NJ, Thacher TD, Pfitzner MA, Fischer PR, Pettifor JM. Causes of lead toxicity in a Nigerian city. Arch Dis Child. 2005; 90(3): 262-6
- Yakasai IA, Gaya SA. Maternal and fetal outcome in patients with eclampsia at Murtala Muhammad specialist Hospital Kano, Nigeria. Ann Afr Med. 2011; 10(4): 305-9
- Yilgwan CS, Okolo SN. Prevalence of diarrhea disease and risk factors in Jos University Teaching Hospital, Nigeria. Ann Afr Med. 2012; 11(4): 217-21

Figure S1. Changes in 25 leading causes of age standardised YLLs in Nigeria and percent changes in all ages, 1998–2019

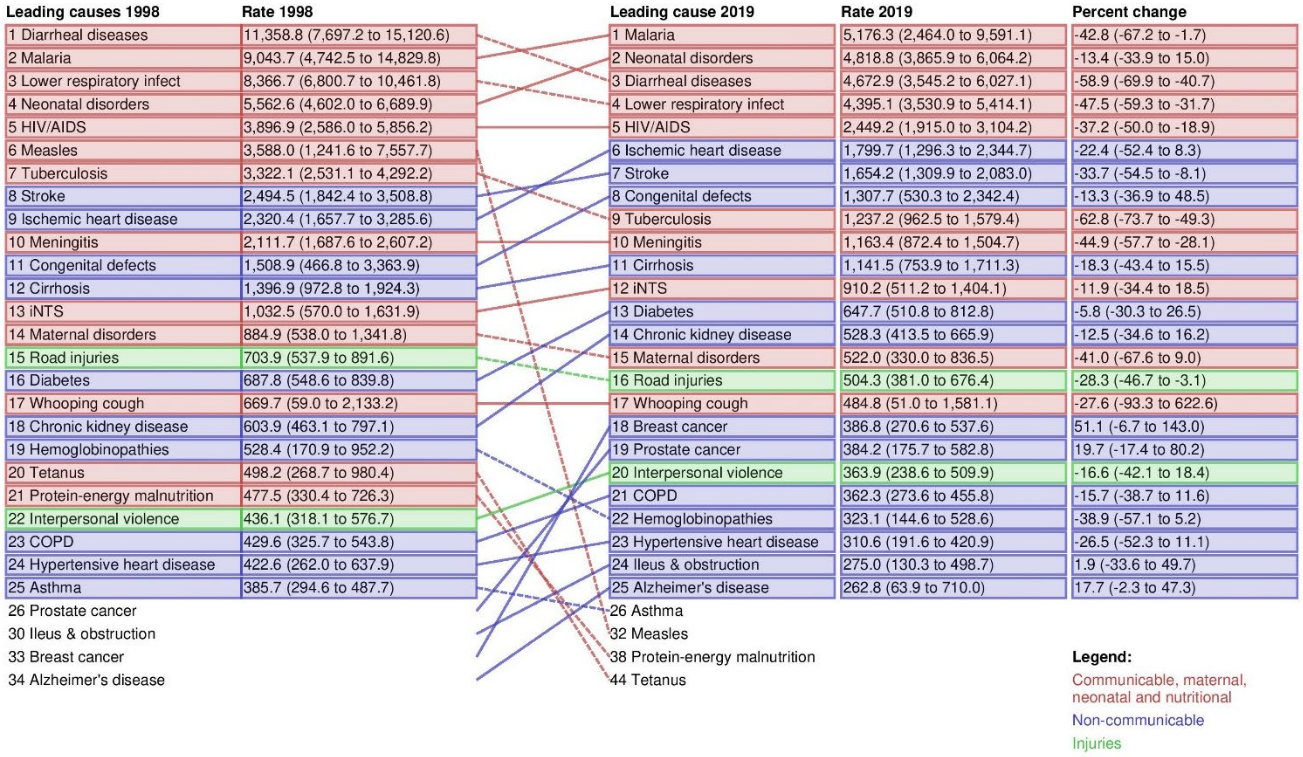

Causes are connected by lines between time periods showing the leading causes of age-standardised YLLs in Nigeria in 1998 and 2019. The rate of YLLs per 100,000 population are shown in 1998 and 2019 with 95% CI and the percent change between the years. COPD=chronic obstructive pulmonary disease. YLL=Years of life lost

Figure S2. Changes in the 25 leading causes of age-standardised YLLs in Nigeria and percent changes for age 20-54 years, 1998–2019.

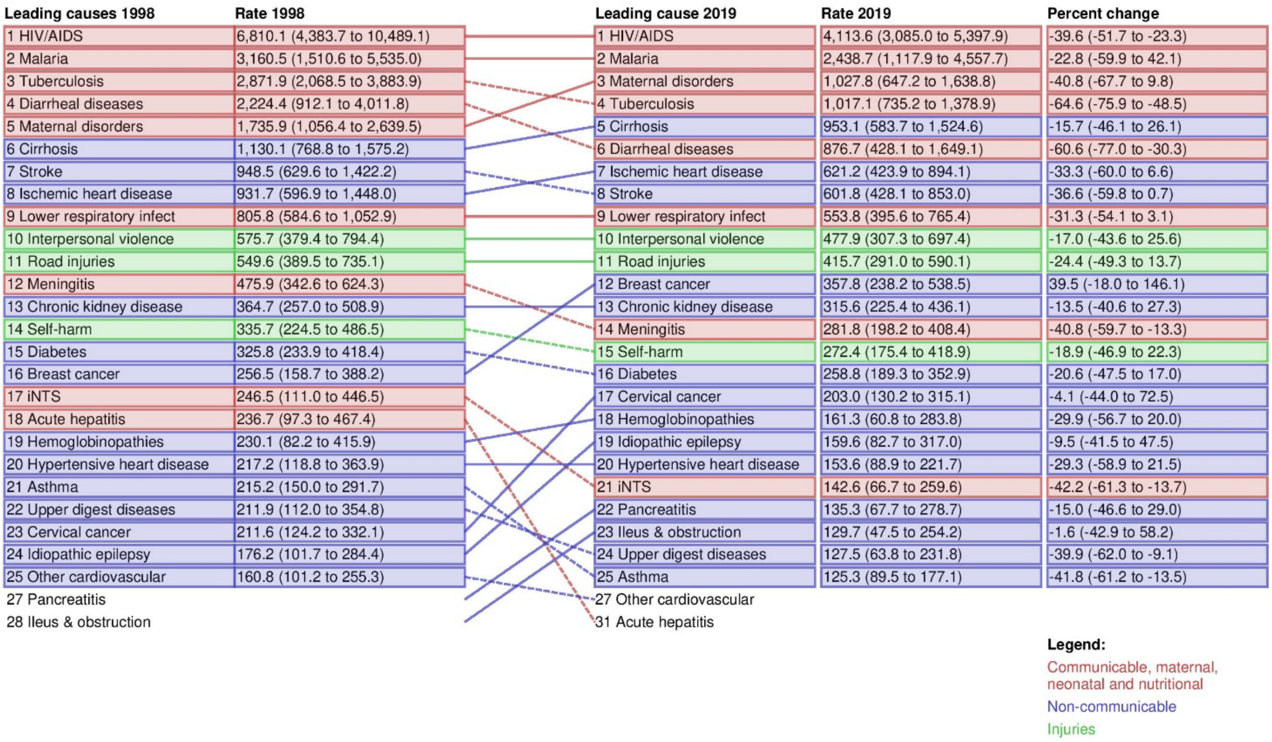

Causes are connected by lines between time periods showing the leading causes of age-standardised YLLs in 20-54 year olds in Nigeria in 1998 and 2019. The rate of YLLs per 100,000 population are shown for each year with 95% CI and the percent change between the years. COPD=chronic obstructive pulmonary disease. YLL=Years of life lost.

Figure S3 - Rank of age standardised DALYs for the top 15 causes in Nigeria across West African countries. A. 1998 B. 2018

A.

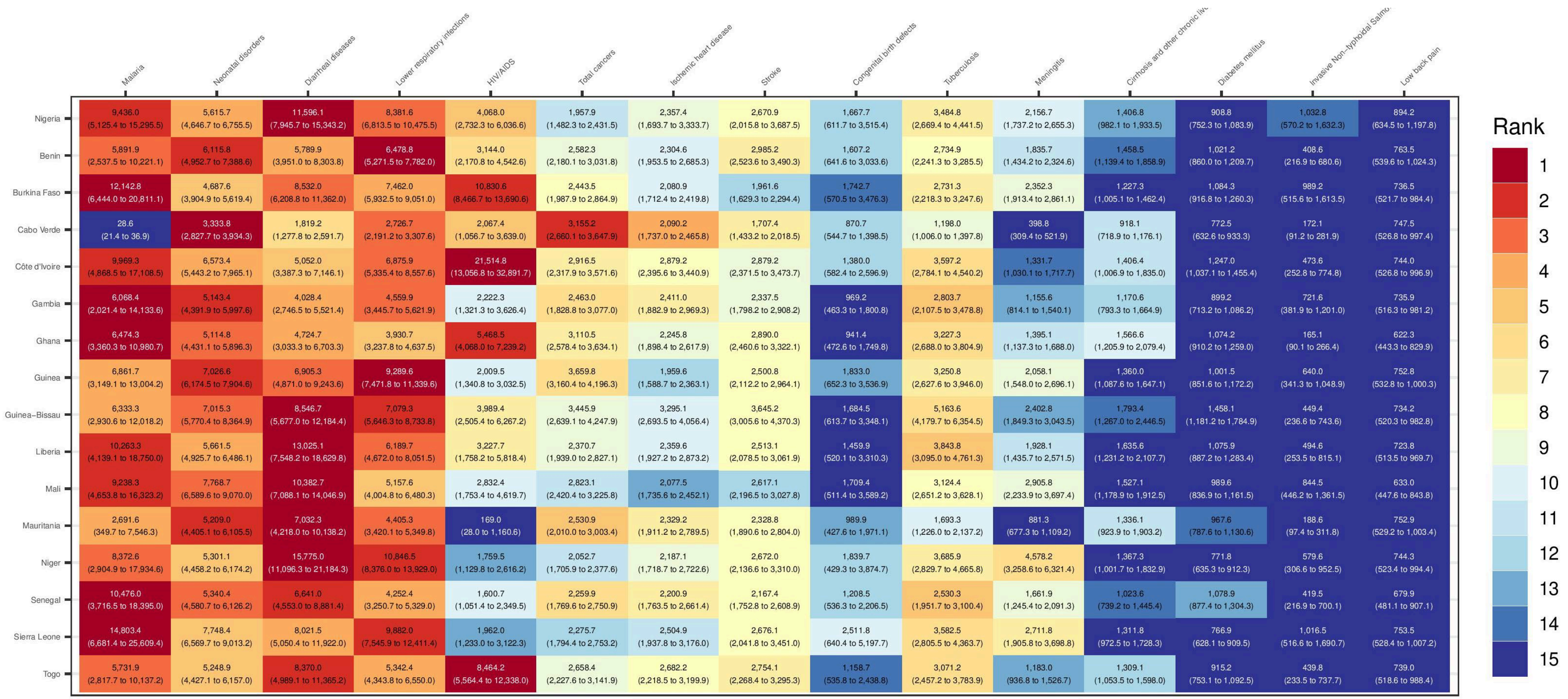

B.

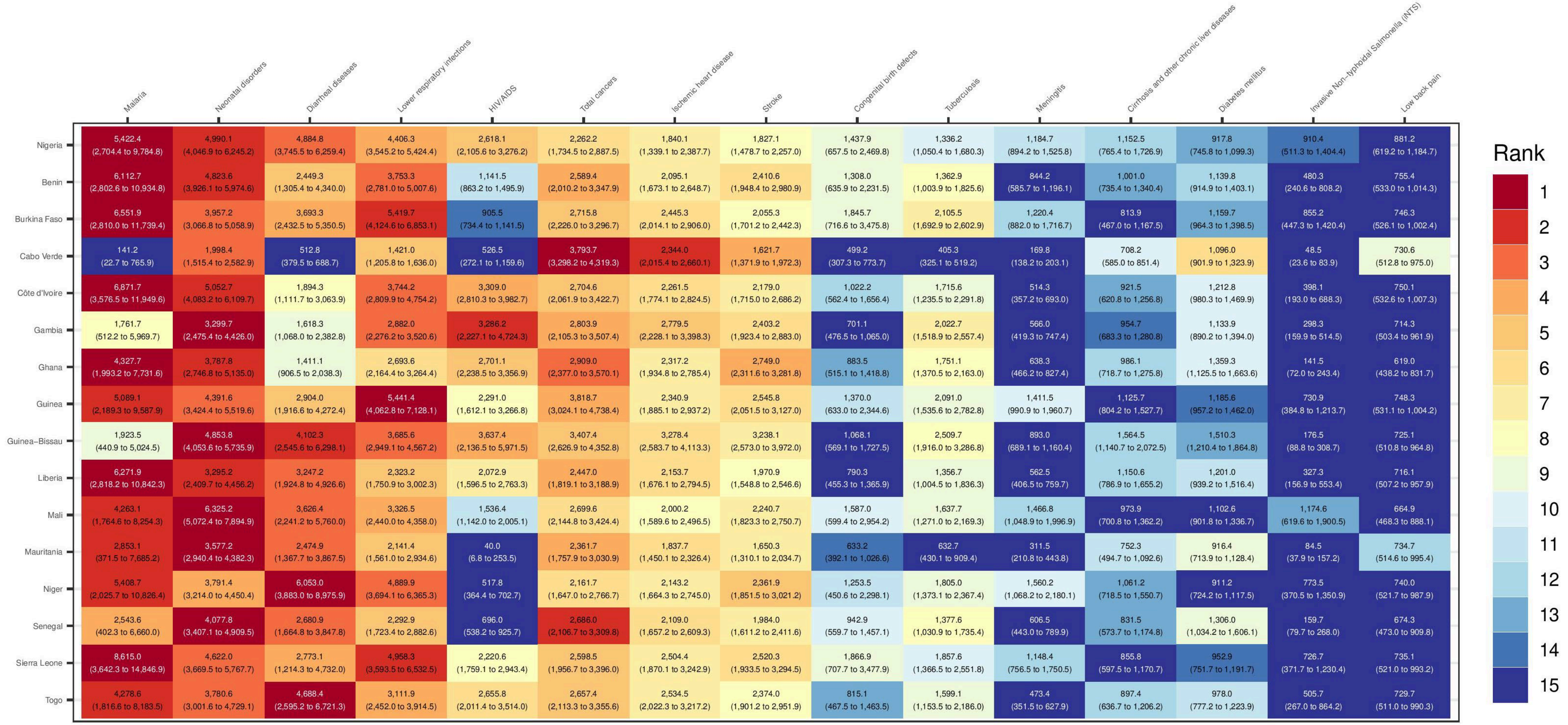
Malaria
Neonatal disorders
Diarrheal diseases
Lower respiratory infections
HIV/AIDS
Total cancers
Ischemic heart disease
Stroke
Congenital birth defects
Tuberculosis
Meningitis
Cirrhosis and other chronic liver diseases
Diabetes mellitus
Invasive Non-typhoidal Salmonella (iNTS)
Low back pain
Rank
1
2
3
4
5
6
7
8
9
10
11
12
13
14
15
Nigeria
5,422.4 (2,704.4 to 9,784.8)
4,990.1 (4,046.9 to 6,245.2)
4,884.8 (3,745.5 to 6,259.4)
4,406.3 (3,545.2 to 5,424.4)
2,618.1 (2,105.6 to 3,276.2)
2,262.2 (1,734.5 to 2,887.5)
1,840.1 (1,339.1 to 2,387.7)
1,827.1 (1,478.7 to 2,257.0)
1,437.9 (657.5 to 2,469.8)
1,336.2 (1,050.4 to 1,680.3)
1,184.7 (894.2 to 1,525.8)
1,152.5 (765.4 to 1,726.9)
917.8 (745.8 to 1,099.3)
910.4 (511.3 to 1,404.4)
881.2 (619.2 to 1,184.7)
Benin
6,112.7 (2,802.6 to 10,934.8)
4,823.6 (3,926.1 to 5,974.6)
2,449.3 (1,305.4 to 4,340.0)
3,753.3 (2,781.0 to 5,007.6)
1,141.5 (863.2 to 1,495.9)
2,589.4 (2,010.2 to 3,347.9)
2,095.1 (1,673.1 to 2,648.7)
2,410.6 (1,948.4 to 2,980.9)
1,308.0 (635.9 to 2,231.5)
1,362.9 (1,003.9 to 1,825.6)
844.2 (585.7 to 1,196.1)
1,001.0 (735.4 to 1,340.4)
1,139.8 (914.9 to 1,403.1)
480.3 (240.6 to 808.2)
755.4 (533.0 to 1,014.3)
Burkina Faso
6,551.9 (2,810.0 to 11,739.4)
3,957.2 (3,066.8 to 5,058.9)
3,693.3 (2,432.5 to 5,350.5)
5,419.7 (4,124.6 to 6,853.1)
905.5 (734.4 to 1,141.5)
2,715.8 (2,226.0 to 3,296.7)
2,445.3 (2,014.1 to 2,906.0)
2,055.3 (1,701.2 to 2,442.3)
1,845.7 (716.6 to 3,475.8)
2,105.5 (1,692.9 to 2,602.9)
1,220.4 (882.0 to 1,716.7)
813.9 (467.0 to 1,167.5)
1,159.7 (964.3 to 1,398.5)
855.2 (447.3 to 1,420.4)
746.3 (526.1 to 1,002.4)
Cabo Verde
141.2 (22.7 to 765.9)
1,998.4 (1,515.4 to 2,582.9)
512.8 (379.5 to 688.7)
1,421.0 (1,205.8 to 1,636.0)
526.5 (272.1 to 1,159.6)
3,793.7 (3,298.2 to 4,319.3)
2,344.0 (2,015.4 to 2,660.1)
1,621.7 (1,371.9 to 1,972.3)
499.2 (307.3 to 773.7)
405.3 (325.1 to 519.2)
169.8 (138.2 to 203.1)
708.2 (585.0 to 851.4)
1,096.0 (901.9 to 1,323.9)
48.5 (23.6 to 83.9)
730.6 (512.8 to 975.0)
Côte d'Ivoire
6,871.7 (3,576.5 to 11,949.6)
5,052.7 (4,083.2 to 6,109.7)
1,894.3 (1,111.7 to 3,063.9)
3,744.2 (2,809.9 to 4,754.2)
3,309.0 (2,810.3 to 3,982.7)
2,704.6 (2,061.9 to 3,422.7)
2,261.5 (1,774.1 to 2,824.5)
2,179.0 (1,715.0 to 2,686.2)
1,022.2 (562.4 to 1,656.4)
1,715.6 (1,235.5 to 2,291.8)
514.3 (357.2 to 693.0)
921.5 (620.8 to 1,256.8)
1,212.8 (980.3 to 1,469.9)
398.1 (193.0 to 688.3)
750.1 (532.6 to 1,007.3)
Gambia
1,761.7 (512.2 to 5,969.7)
3,299.7 (2,475.4 to 4,426.0)
1,618.3 (1,068.0 to 2,382.8)
2,882.0 (2,276.2 to 3,520.6)
3,286.2 (2,227.1 to 4,724.3)
2,803.9 (2,105.3 to 3,507.4)
2,779.5 (2,228.1 to 3,398.3)
2,403.2 (1,923.4 to 2,883.0)
701.1 (476.5 to 1,065.0)
2,022.7 (1,518.9 to 2,557.4)
566.0 (419.3 to 747.4)
954.7 (683.3 to 1,280.8)
1,133.9 (890.2 to 1,394.0)
298.3 (159.9 to 514.5)
714.3 (503.4 to 961.9)
Ghana
4,327.7 (1,993.2 to 7,731.6)
3,787.8 (2,746.8 to 5,135.0)
1,411.1 (906.5 to 2,038.3)
2,693.6 (2,164.4 to 3,264.4)
2,701.1 (2,238.5 to 3,356.9)
2,909.0 (2,377.0 to 3,570.1)
2,317.2 (1,934.8 to 2,785.4)
2,749.0 (2,311.6 to 3,281.8)
883.5 (515.1 to 1,418.8)
1,751.1 (1,370.5 to 2,163.0)
638.3 (466.2 to 827.4)
986.1 (718.7 to 1,275.8)
1,359.3 (1,125.5 to 1,663.6)
141.5 (72.0 to 243.4)
619.0 (438.2 to 831.7)
Guinea
5,089.1 (2,189.3 to 9,587.9)
4,391.6 (3,424.4 to 5,519.6)
2,904.0 (1,916.6 to 4,272.4)
5,441.4 (4,062.8 to 7,128.1)
2,291.0 (1,612.1 to 3,266.8)
3,818.7 (3,024.1 to 4,738.4)
2,340.9 (1,885.1 to 2,937.2)
2,545.8 (2,051.5 to 3,127.0)
1,370.0 (633.0 to 2,344.6)
2,091.0 (1,535.6 to 2,782.8)
1,411.5 (990.9 to 1,960.7)
1,125.7 (804.2 to 1,527.7)
1,185.6 (957.2 to 1,462.0)
730.9 (384.8 to 1,213.7)
748.3 (531.1 to 1,004.2)
Guinea-Bissau
1,923.5 (440.9 to 5,024.5)
4,853.8 (4,053.6 to 5,735.9)
4,102.3 (2,545.6 to 6,298.1)
3,685.6 (2,949.1 to 4,567.2)
3,637.4 (2,136.5 to 5,971.5)
3,407.4 (2,626.9 to 4,352.8)
3,278.4 (2,583.7 to 4,113.3)
3,238.1 (2,573.0 to 3,972.0)
1,068.1 (569.1 to 1,727.5)
2,509.7 (1,916.0 to 3,286.8)
893.0 (689.1 to 1,160.4)
1,564.5 (1,140.7 to 2,072.5)
1,510.3 (1,210.4 to 1,864.8)
176.5 (88.8 to 308.7)
725.1 (510.8 to 964.8)
Liberia
6,271.9 (2,818.2 to 10,842.3)
3,295.2 (2,409.7 to 4,456.2)
3,247.2 (1,924.8 to 4,926.6)
2,323.2 (1,750.9 to 3,002.3)
2,072.9 (1,596.5 to 2,763.3)
2,447.0 (1,819.1 to 3,188.9)
2,153.7 (1,676.1 to 2,794.5)
1,970.9 (1,548.8 to 2,546.6)
790.3 (455.3 to 1,365.9)
1,356.7 (1,004.5 to 1,836.3)
562.5 (406.5 to 759.7)
1,150.6 (786.9 to 1,655.2)
1,201.0 (939.2 to 1,516.4)
327.3 (156.9 to 553.4)
716.1 (507.2 to 957.9)
Mali
4,263.1 (1,764.6 to 8,254.3)
6,325.2 (5,072.4 to 7,894.9)
3,626.4 (2,241.2 to 5,760.0)
3,326.5 (2,440.0 to 4,358.0)
1,536.4 (1,142.0 to 2,005.1)
2,699.6 (2,144.8 to 3,424.4)
2,000.2 (1,589.6 to 2,496.5)
2,240.7 (1,823.3 to 2,750.7)
1,587.0 (599.4 to 2,954.2)
1,637.7 (1,271.0 to 2,169.3)
1,466.8 (1,048.9 to 1,996.9)
973.9 (700.8 to 1,362.2)
1,102.6 (901.8 to 1,336.7)
1,174.6 (619.6 to 1,900.5)
664.9 (468.3 to 888.1)
Mauritania
2,853.1 (371.5 to 7,685.2)
3,577.2 (2,940.4 to 4,382.3)
2,474.9 (1,367.7 to 3,867.5)
2,141.4 (1,561.0 to 2,934.6)
40.0 (6.8 to 253.5)
2,361.7 (1,757.9 to 3,030.9)
1,837.7 (1,450.1 to 2,326.4)
1,650.3 (1,310.1 to 2,034.7)
633.2 (392.1 to 1,026.6)
632.7 (430.1 to 909.4)
311.5 (210.8 to 443.8)
752.3 (494.7 to 1,092.6)
916.4 (713.9 to 1,128.4)
84.5 (37.9 to 157.2)
734.7 (514.6 to 995.4)
Niger
5,408.7 (2,025.7 to 10,826.4)
3,791.4 (3,214.0 to 4,450.4)
6,053.0 (3,883.0 to 8,975.9)
4,889.9 (3,694.1 to 6,365.3)
517.8 (364.4 to 702.7)
2,161.7 (1,647.0 to 2,766.7)
2,143.2 (1,664.3 to 2,745.0)
2,361.9 (1,851.5 to 3,021.2)
1,253.5 (450.6 to 2,298.1)
1,805.0 (1,373.1 to 2,367.4)
1,560.2 (1,068.2 to 2,180.1)
1,061.2 (718.5 to 1,550.7)
911.2 (724.2 to 1,117.5)
773.5 (370.5 to 1,350.9)
740.0 (521.7 to 987.9)
Senegal
2,543.6 (402.3 to 6,660.0)
4,077.8 (3,407.1 to 4,909.5)
2,680.9 (1,664.8 to 3,847.8)
2,292.9 (1,723.4 to 2,882.6)
696.0 (538.2 to 925.7)
2,686.0 (2,106.7 to 3,309.8)
2,109.0 (1,657.2 to 2,609.3)
1,984.0 (1,611.2 to 2,411.6)
942.9 (559.7 to 1,457.1)
1,377.6 (1,030.9 to 1,735.4)
606.5 (443.0 to 789.9)
831.5 (573.7 to 1,174.8)
1,306.0 (1,034.2 to 1,606.1)
159.7 (79.7 to 268.0)
674.3 (473.0 to 909.8)
Sierra Leone
8,615.0 (3,642.3 to 14,846.9)
4,622.0 (3,669.5 to 5,767.7)
2,773.1 (1,214.3 to 4,732.0)
4,958.3 (3,593.5 to 6,532.5)
2,220.6 (1,759.1 to 2,943.4)
2,598.5 (1,956.7 to 3,396.0)
2,504.4 (1,870.1 to 3,242.9)
2,520.3 (1,933.5 to 3,294.5)
1,866.9 (707.7 to 3,477.9)
1,857.6 (1,366.5 to 2,551.8)
1,148.4 (756.5 to 1,750.5)
855.8 (597.5 to 1,170.7)
952.9 (751.7 to 1,191.7)
726.7 (371.7 to 1,230.4)
735.1 (521.0 to 993.2)
Togo
4,278.6 (1,816.6 to 8,183.5)
3,780.6 (3,001.6 to 4,729.1)
4,688.4 (2,595.2 to 6,721.3)
3,111.9 (2,452.0 to 3,914.5)
2,655.8 (2,011.4 to 3,514.0)
2,657.4 (2,113.3 to 3,355.6)
2,534.5 (2,022.3 to 3,217.2)
2,374.0 (1,901.2 to 2,951.9)
815.1 (467.5 to 1,463.5)
1,599.1 (1,153.5 to 2,186.0)
473.4 (351.5 to 627.9)
897.4 (636.7 to 1,206.2)
978.0 (777.2 to 1,223.9)
505.7 (267.0 to 864.2)
729.7 (511.0 to 990.3)

Figure S4. Trends in the 25 leading causes of age-standardised YLDs in Nigeria and percent changes for all ages, 1998–2019.

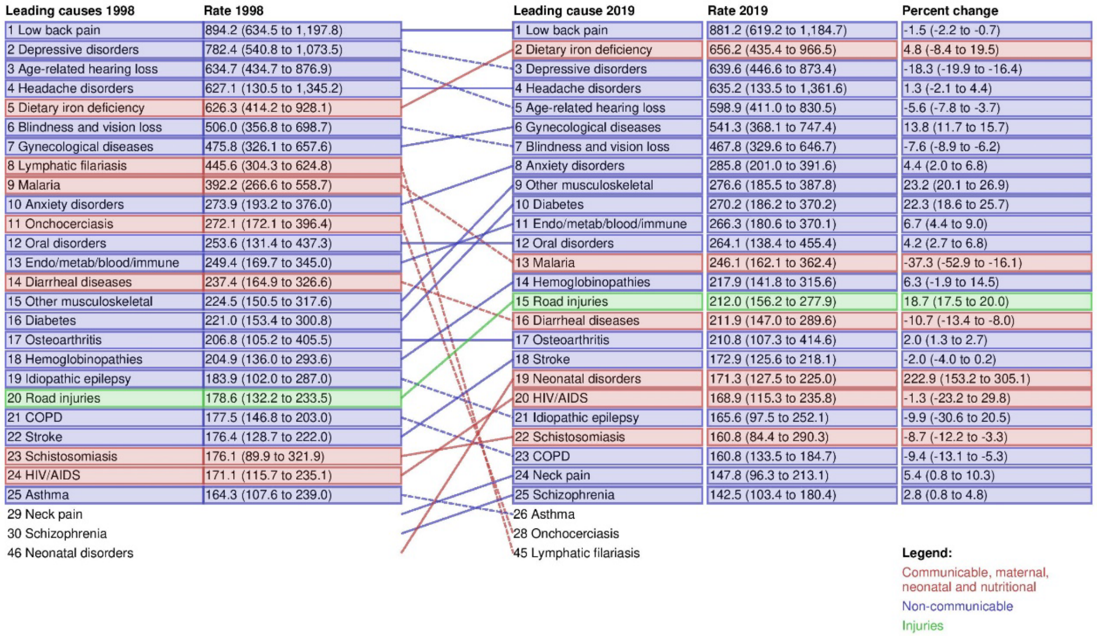

Causes are connected by lines between time periods showing the leading causes of YLDs in Nigeria in 1998 and 2019. The rate of YLDs per 100,000 population are shown for each year with 95% CI and the percent change between the years. COPD=chronic obstructive pulmonary disease. YLD=Years lived with disability.

Figure S5. Age-specific YLDs rate per 100,000 population in Nigeria for both sexes, 2019

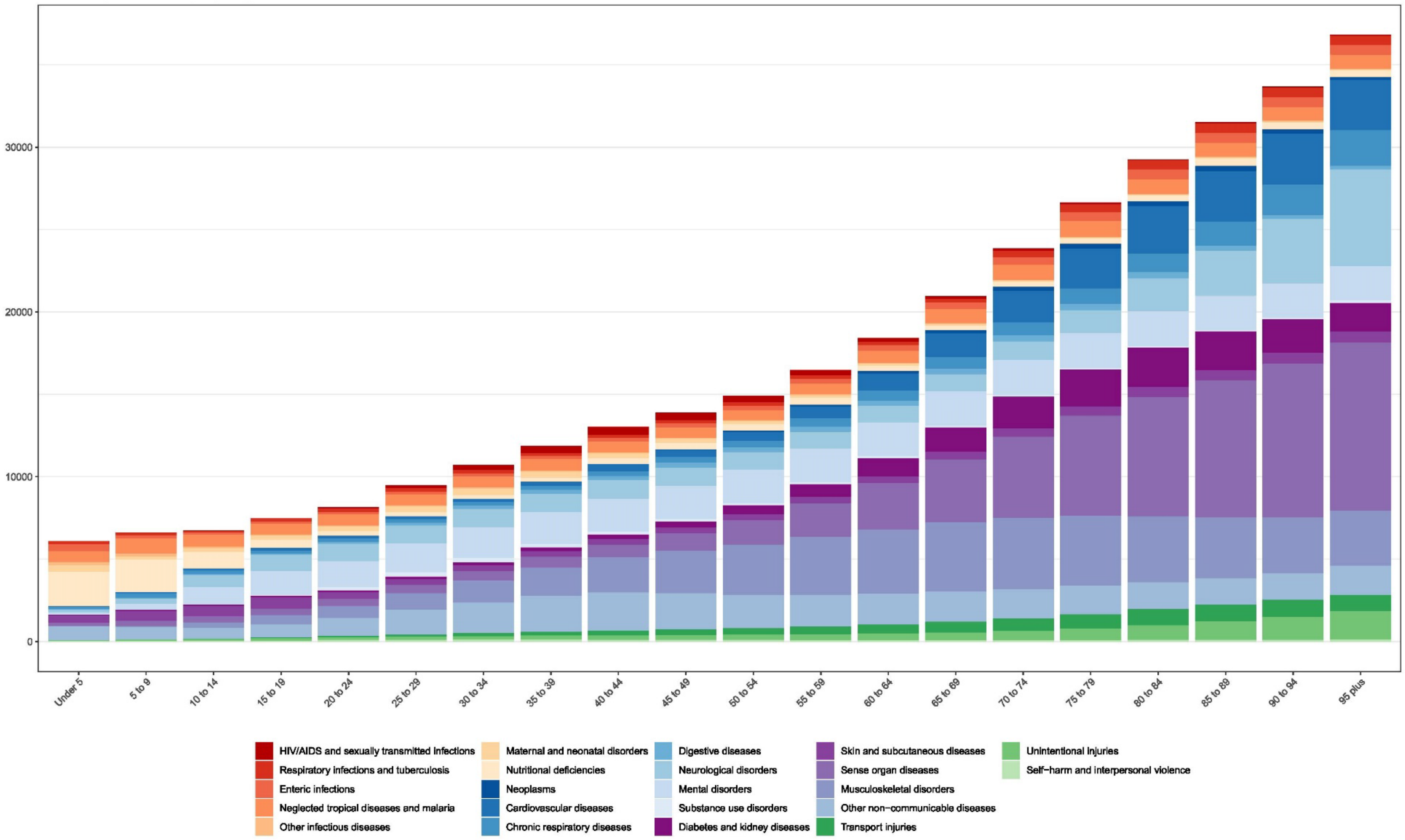

Figure S6. Percentage of DALYs attributable to 20 risk factors for all ages in Nigeria, 2019

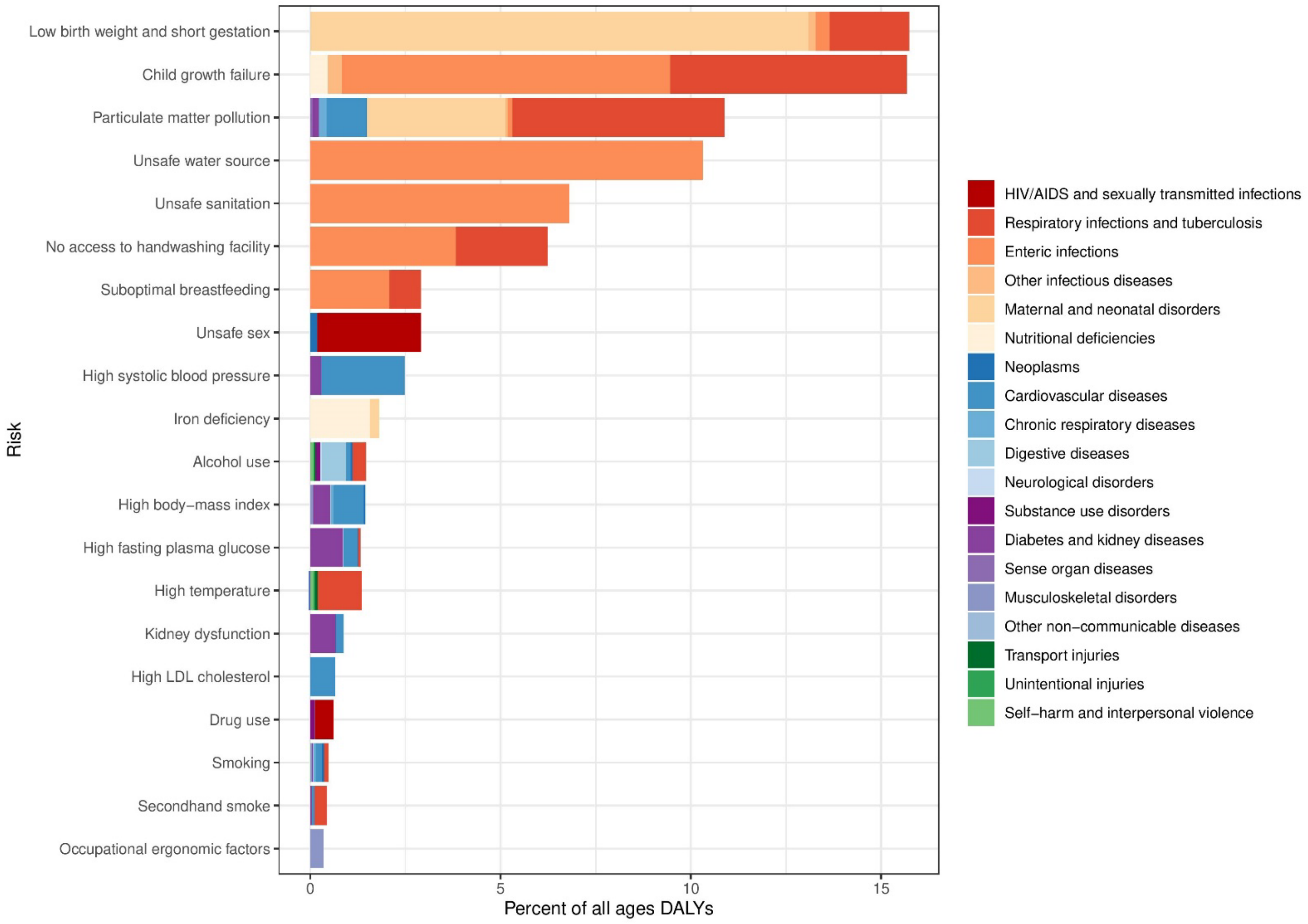

Figure S7. Historical trends in the proportion of deaths in Nigeria attributable to metabolic risk factors

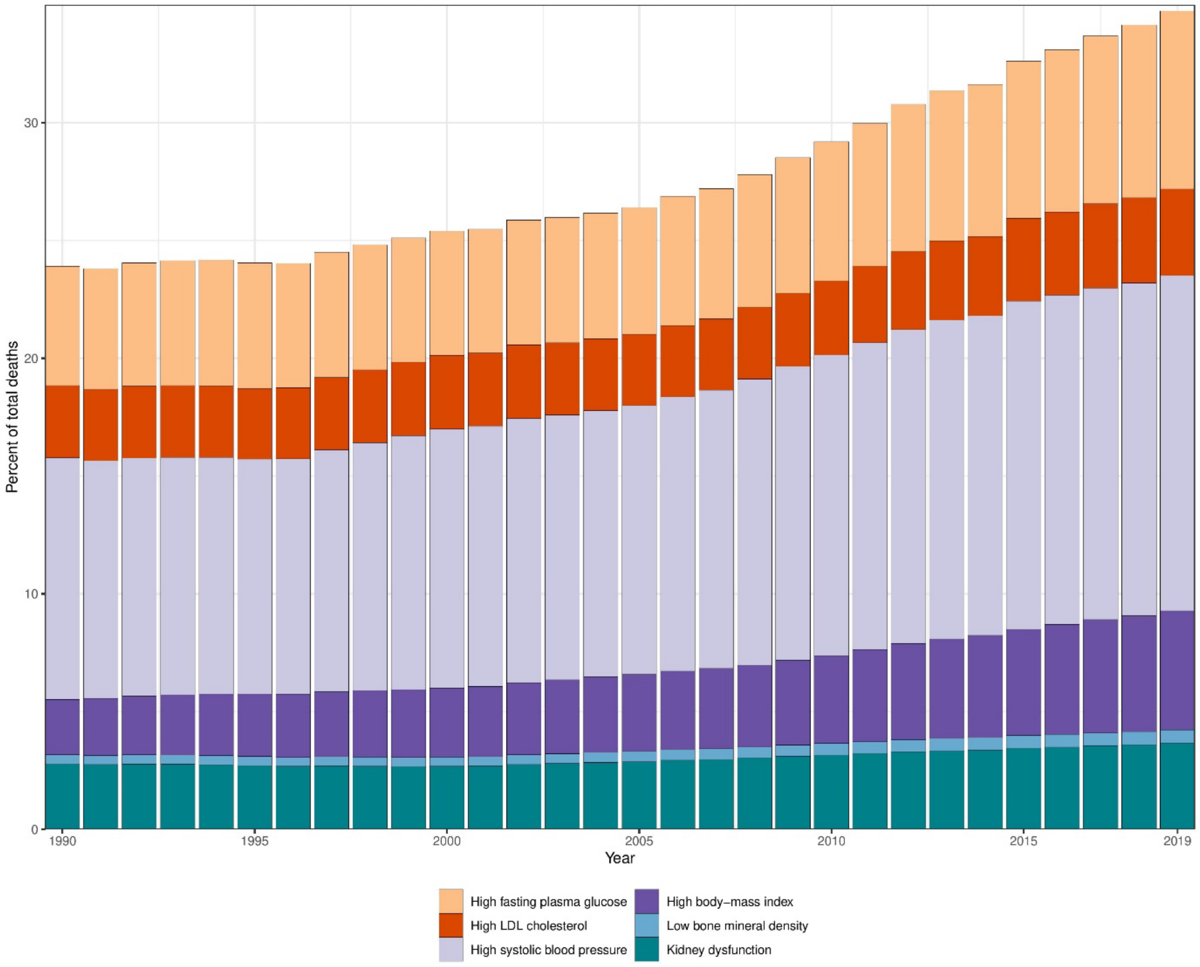

Table S2 – All age and age-standardised DALYs for all causes, Nigeria 1998 and 2019

	All ages		Age standardized	
	1998	2019	1998	2019
All causes	104,561.9 (97,567.5 to 111,625.7)	54,038.4 (46,790.0 to 62,251.9)	82,842.9 (75,793.0 to 90,156.9)	53,229.6 (46,948.0 to 59,822.3)
Communicable, maternal, neonatal, and nutritional diseases	83,401.8 (76,892.9 to 90,018.9)	37,170.3 (31,183.5 to 44,660.0)	55,633.7 (50,838.4 to 60,684.2)	29,467.8 (25,334.2 to 34,614.3)
HIV/AIDS and sexually transmitted infections	3,868.3 (2,600.0 to 5,660.5)	2,428.2 (1,872.2 to 3,148.9)	4,261.1 (2,867.7 to 6,300.0)	2,861.6 (2,294.6 to 3,550.3)
HIV/AIDS	3,500.9 (2,316.5 to 5,251.5)	2,045.2 (1,586.9 to 2,620.5)	4,068.0 (2,732.3 to 6,036.6)	2,618.1 (2,105.6 to 3,276.2)
HIV/AIDS - Drug-susceptible Tuberculosis	1,219.9 (861.6 to 1,706.8)	325.4 (211.1 to 483.7)	1,421.7 (1,006.1 to 1,971.1)	417.8 (272.3 to 616.8)
HIV/AIDS - Multidrug-resistant Tuberculosis without extensive drug resistance	57.1 (8.1 to 206.7)	34.8 (7.0 to 99.1)	66.2 (9.6 to 236.9)	44.5 (8.9 to 126.2)
HIV/AIDS - Extensively drug-resistant Tuberculosis	0.2 (0.0 to 0.6)	0.5 (0.1 to 1.3)	0.2 (0.0 to 0.7)	0.6 (0.1 to 1.7)
HIV/AIDS resulting in other diseases	2,223.7 (1,312.4 to 3,801.5)	1,684.5 (1,198.2 to 2,274.3)	2,579.9 (1,554.8 to 4,421.6)	2,155.3 (1,589.3 to 2,818.2)
Sexually transmitted infections excluding HIV	367.4 (133.3 to 777.9)	383.0 (134.6 to 850.5)	193.1 (82.2 to 380.7)	243.5 (93.1 to 521.7)
Syphilis	338.2 (104.5 to 749.1)	362.7 (115.7 to 828.8)	158.7 (50.2 to 349.6)	218.1 (70.0 to 497.5)
Chlamydial infection	4.0 (2.7 to 5.7)	2.4 (1.6 to 3.6)	4.5 (3.0 to 6.5)	2.9 (1.9 to 4.3)
Gonococcal infection	5.1 (2.4 to 8.0)	2.1 (1.5 to 2.9)	5.7 (2.8 to 8.8)	2.4 (1.7 to 3.3)
Trichomoniasis	6.6 (2.6 to 13.6)	6.1 (2.3 to 12.5)	8.0 (3.0 to 16.3)	7.7 (3.0 to 15.8)
Genital herpes	3.9 (1.3 to 9.4)	3.9 (1.3 to 9.4)	5.4 (1.7 to 13.2)	5.5 (1.8 to 13.6)
Other sexually transmitted infections	9.6 (6.5 to 13.3)	5.9 (3.9 to 8.4)	10.9 (7.3 to 15.3)	6.9 (4.6 to 10.1)
Respiratory infections and tuberculosis	17,333.9 (14,168.3 to 21,462.7)	6,935.1 (5,473.6 to 8,662.6)	11,982.3 (9,994.5 to 14,237.0)	5,844.7 (4,813.4 to 7,065.8)

Tuberculosis	2,873.7 (2,242.7 to 3,606.1)	964.7 (757.3 to 1,190.5)	3,484.8 (2,669.4 to 4,441.5)	1,336.2 (1,050.4 to 1,680.3)
Latent tuberculosis infection	0.0 (0.0 to 0.0)	0.0 (0.0 to 0.0)	0.0 (0.0 to 0.0)	0.0 (0.0 to 0.0)
Drug-susceptible tuberculosis	2,764.8 (2,103.7 to 3,493.1)	888.1 (677.6 to 1,122.8)	3,352.2 (2,538.6 to 4,278.7)	1,227.6 (933.3 to 1,582.2)
Multidrug-resistant tuberculosis without extensive drug resistance	108.6 (15.7 to 371.9)	75.6 (16.6 to 215.2)	132.2 (19.5 to 461.7)	107.2 (23.5 to 306.6)
Extensively drug-resistant tuberculosis	0.3 (0.0 to 1.1)	1.0 (0.2 to 3.0)	0.4 (0.1 to 1.3)	1.4 (0.3 to 4.2)
Lower respiratory infections	14,328.4 (11,398.9 to 18,179.7)	5,858.0 (4,522.2 to 7,485.8)	8,381.6 (6,813.5 to 10,475.5)	4,406.3 (3,545.2 to 5,424.4)
Upper respiratory infections	92.2 (48.3 to 148.0)	78.3 (47.5 to 121.7)	79.7 (44.5 to 124.4)	71.5 (43.6 to 108.5)
Otitis media	39.5 (22.8 to 64.6)	34.1 (19.8 to 55.7)	36.3 (21.0 to 59.1)	30.8 (17.8 to 50.1)
Enteric infections	19,481.5 (14,237.1 to 24,320.3)	7,884.6 (6,000.5 to 10,126.6)	12,812.7 (9,019.7 to 16,491.7)	5,896.4 (4,522.3 to 7,427.3)
Diarrheal diseases	17,640.9 (12,502.1 to 22,561.8)	6,418.6 (4,872.7 to 8,311.6)	11,596.1 (7,945.7 to 15,343.2)	4,884.8 (3,745.5 to 6,259.4)
Typhoid and paratyphoid	217.8 (92.2 to 419.7)	132.5 (56.2 to 252.7)	165.9 (72.5 to 319.1)	96.2 (41.2 to 178.4)
Typhoid fever	207.6 (88.2 to 401.6)	127.3 (54.2 to 240.4)	158.5 (68.9 to 305.9)	92.5 (39.8 to 174.2)
Paratyphoid fever	10.1 (3.7 to 22.7)	5.2 (1.8 to 11.4)	7.4 (2.8 to 16.3)	3.7 (1.3 to 7.9)
Invasive Non-typhoidal Salmonella (iNTS)	1,592.0 (868.5 to 2,530.8)	1,325.8 (747.6 to 2,080.3)	1,032.8 (570.2 to 1,632.3)	910.4 (511.3 to 1,404.4)
Other intestinal infectious diseases	30.9 (19.4 to 47.8)	7.7 (4.5 to 12.0)	17.8 (11.2 to 27.4)	5.1 (3.0 to 7.8)
Neglected tropical diseases and malaria	14,914.0 (8,987.6 to 23,430.0)	6,728.8 (3,684.6 to 11,411.7)	11,111.5 (6,785.6 to 17,038.1)	6,128.7 (3,342.2 to 10,350.0)
Malaria	13,273.8 (7,194.1 to 21,789.9)	6,027.6 (2,992.5 to 10,788.6)	9,436.0 (5,125.4 to 15,295.5)	5,422.4 (2,704.4 to 9,784.8)
Chagas disease	0.0 (0.0 to 0.0)	0.0 (0.0 to 0.0)	0.0 (0.0 to 0.0)	0.0 (0.0 to 0.0)

Leishmaniasis	0.3 (0.1 to 0.4)	0.6 (0.3 to 0.9)	0.2 (0.1 to 0.4)	0.4 (0.2 to 0.7)
Visceral leishmaniasis	0.2 (0.1 to 0.4)	0.5 (0.3 to 0.8)	0.1 (0.1 to 0.2)	0.4 (0.2 to 0.6)
Cutaneous and mucocutaneous leishmaniasis	0.1 (0.0 to 0.1)	0.0 (0.0 to 0.1)	0.1 (0.0 to 0.2)	0.1 (0.0 to 0.1)
African trypanosomiasis	0.1 (0.0 to 0.2)	0.0 (0.0 to 0.0)	0.1 (0.0 to 0.2)	0.0 (0.0 to 0.0)
Schistosomiasis	232.4 (150.2 to 372.6)	180.7 (107.2 to 301.3)	281.0 (190.0 to 437.5)	204.8 (125.6 to 335.0)
Cysticercosis	21.2 (12.5 to 32.6)	10.3 (6.2 to 15.9)	39.6 (22.7 to 61.6)	20.9 (12.2 to 33.3)
Cystic echinococcosis	3.2 (1.2 to 5.9)	0.8 (0.3 to 1.5)	2.3 (1.0 to 3.9)	0.7 (0.3 to 1.2)
Lymphatic filariasis	361.7 (243.9 to 507.5)	44.8 (26.6 to 73.9)	445.6 (304.3 to 624.8)	53.9 (32.9 to 87.2)
Onchocerciasis	210.0 (126.6 to 311.0)	90.4 (49.4 to 142.2)	272.1 (172.1 to 396.4)	126.2 (73.3 to 195.6)
Trachoma	23.2 (15.9 to 32.6)	8.6 (5.6 to 12.7)	50.1 (34.1 to 69.6)	22.2 (14.2 to 33.1)
Dengue	7.8 (3.7 to 14.2)	6.0 (1.1 to 13.9)	7.8 (3.8 to 14.0)	6.2 (1.6 to 13.5)
Yellow fever	128.7 (45.9 to 274.7)	30.0 (10.9 to 64.8)	110.4 (39.7 to 235.5)	24.5 (8.9 to 52.7)
Rabies	80.8 (17.6 to 152.4)	42.8 (9.7 to 77.1)	59.9 (12.5 to 110.0)	34.4 (7.8 to 61.4)
Intestinal nematode infections	282.9 (198.2 to 393.1)	78.6 (52.5 to 113.8)	222.3 (150.7 to 317.5)	63.2 (41.3 to 93.0)
Ascariasis	162.2 (116.6 to 218.7)	44.6 (31.0 to 62.8)	115.6 (81.1 to 158.2)	34.4 (23.2 to 49.7)
Trichuriasis	26.5 (14.4 to 45.7)	5.5 (2.7 to 9.7)	24.7 (13.4 to 42.5)	4.9 (2.4 to 8.8)
Hookworm disease	94.1 (58.6 to 142.0)	28.4 (16.6 to 45.0)	82.0 (50.7 to 124.7)	23.8 (13.8 to 38.1)
Food-borne trematodiases	0.0 (0.0 to 0.0)	0.0 (0.0 to 0.0)	0.0 (0.0 to 0.0)	0.0 (0.0 to 0.0)
Leprosy	0.3 (0.2 to 0.5)	0.5 (0.3 to 0.8)	0.5 (0.3 to 0.8)	0.8 (0.5 to 1.2)

Ebola	0.0 (0.0 to 0.0)	0.0 (0.0 to 0.0)	0.0 (0.0 to 0.0)	0.0 (0.0 to 0.0)
Zika virus	0.0 (0.0 to 0.0)	0.0 (0.0 to 0.0)	0.0 (0.0 to 0.0)	0.0 (0.0 to 0.0)
Guinea worm disease	0.1 (0.1 to 0.2)	0.0 (0.0 to 0.0)	0.2 (0.1 to 0.3)	0.0 (0.0 to 0.0)
Other neglected tropical diseases	287.7 (154.2 to 890.3)	207.1 (123.1 to 530.7)	183.5 (101.3 to 539.8)	148.2 (88.8 to 368.5)
Other infectious diseases	12,894.7 (8,227.7 to 19,758.8)	3,223.9 (2,136.6 to 5,025.6)	7,660.4 (5,020.6 to 11,600.3)	2,268.8 (1,573.5 to 3,441.1)
Meningitis	3,479.0 (2,771.7 to 4,344.3)	1,643.4 (1,216.3 to 2,167.4)	2,156.7 (1,737.2 to 2,655.3)	1,184.7 (894.2 to 1,525.8)
Encephalitis	35.9 (28.1 to 51.5)	34.9 (25.8 to 51.9)	28.5 (21.8 to 44.4)	30.3 (22.3 to 47.7)
Diphtheria	491.1 (293.1 to 758.1)	123.5 (74.4 to 186.8)	284.4 (171.4 to 439.0)	80.0 (48.3 to 120.7)
Whooping cough	1,226.8 (114.9 to 3,851.9)	770.0 (86.9 to 2,493.7)	675.3 (63.3 to 2,138.1)	489.4 (55.2 to 1,585.9)
Tetanus	899.7 (530.3 to 1,650.4)	139.9 (87.2 to 229.0)	498.4 (268.9 to 980.7)	97.6 (61.2 to 170.0)
Measles	6,292.8 (2,154.0 to 13,345.0)	270.7 (84.3 to 658.7)	3,593.3 (1,243.2 to 7,570.0)	173.6 (54.0 to 422.8)
Varicella and herpes zoster	54.1 (41.1 to 71.1)	42.9 (31.5 to 57.1)	40.1 (32.0 to 50.0)	34.0 (26.3 to 43.3)
Acute hepatitis	241.8 (112.0 to 473.0)	65.8 (46.8 to 97.5)	269.1 (120.7 to 518.3)	78.9 (56.2 to 119.8)
Acute hepatitis A	111.4 (31.4 to 277.7)	19.4 (11.4 to 39.9)	128.4 (38.0 to 299.3)	22.6 (13.1 to 47.0)
Acute hepatitis B	96.7 (44.3 to 178.3)	36.9 (25.5 to 58.0)	105.3 (47.0 to 201.5)	44.5 (31.4 to 68.9)
Acute hepatitis C	27.5 (10.4 to 53.4)	7.5 (3.5 to 14.8)	29.5 (12.2 to 61.1)	9.5 (4.4 to 19.9)
Acute hepatitis E	6.2 (1.9 to 13.2)	2.0 (0.9 to 3.9)	6.0 (1.9 to 13.1)	2.3 (1.0 to 4.6)
Other unspecified infectious diseases	173.5 (133.9 to 212.0)	132.8 (98.7 to 172.9)	114.5 (90.4 to 140.6)	100.4 (74.4 to 131.1)

Maternal and neonatal disorders	13,051.8 (10,927.3 to 15,593.3)	8,808.3 (7,201.0 to 10,901.3)	6,560.5 (5,496.6 to 7,803.4)	5,581.2 (4,588.4 to 6,810.0)
Maternal disorders	856.0 (540.6 to 1,269.9)	528.1 (351.0 to 816.0)	944.8 (596.6 to 1,401.1)	591.1 (396.2 to 902.1)
Maternal hemorrhage	249.0 (155.5 to 376.9)	148.5 (95.5 to 225.9)	271.6 (169.5 to 407.9)	163.7 (104.8 to 248.0)
Maternal sepsis and other maternal infections	73.9 (43.4 to 118.0)	25.2 (15.6 to 40.6)	77.0 (45.3 to 123.0)	26.8 (16.8 to 42.8)
Maternal hypertensive disorders	75.5 (48.0 to 111.3)	52.7 (34.9 to 80.4)	74.8 (47.5 to 109.9)	53.7 (35.7 to 81.0)
Maternal obstructed labor and uterine rupture	56.2 (37.9 to 78.3)	58.5 (41.1 to 82.7)	69.4 (47.1 to 96.3)	71.3 (50.4 to 99.5)
Maternal abortion and miscarriage	152.3 (86.9 to 238.8)	64.0 (38.5 to 104.7)	178.4 (102.0 to 283.7)	76.7 (46.3 to 122.8)
Ectopic pregnancy	32.6 (19.6 to 50.4)	19.6 (11.9 to 32.1)	34.8 (20.8 to 53.9)	20.9 (12.7 to 34.4)
Indirect maternal deaths	30.0 (17.5 to 46.9)	24.4 (14.9 to 42.0)	31.6 (18.3 to 49.8)	26.5 (16.0 to 44.8)
Late maternal deaths	75.2 (43.2 to 121.8)	60.0 (35.1 to 103.3)	82.2 (46.8 to 135.8)	66.6 (39.0 to 111.2)
Maternal deaths aggravated by HIV/AIDS	12.8 (6.1 to 21.4)	8.4 (4.0 to 14.8)	13.7 (6.5 to 23.3)	9.9 (4.8 to 17.3)
Other maternal disorders	98.5 (58.3 to 151.7)	67.0 (42.9 to 105.1)	111.2 (65.2 to 173.0)	75.2 (48.3 to 117.5)
Neonatal disorders	12,195.8 (10,090.8 to 14,661.7)	8,280.2 (6,702.7 to 10,396.3)	5,615.7 (4,646.7 to 6,755.5)	4,990.1 (4,046.9 to 6,245.2)
Neonatal preterm birth	4,361.2 (3,212.4 to 5,645.1)	3,236.2 (2,475.9 to 4,173.2)	2,013.5 (1,485.3 to 2,604.1)	1,950.2 (1,496.5 to 2,512.7)
Neonatal encephalopathy due to birth asphyxia and trauma	4,973.5 (3,775.4 to 6,384.9)	3,190.0 (2,421.5 to 4,071.3)	2,281.2 (1,731.6 to 2,926.9)	1,914.4 (1,464.6 to 2,439.5)
Neonatal sepsis and other neonatal infections	1,751.3 (1,153.3 to 2,437.9)	1,171.5 (856.7 to 1,589.6)	808.3 (533.7 to 1,122.2)	715.0 (526.2 to 965.3)
Hemolytic disease and other neonatal jaundice	240.2 (168.1 to 350.9)	113.7 (86.9 to 153.5)	112.1 (78.7 to 163.1)	70.2 (54.0 to 94.0)

Other neonatal disorders	869.6 (199.3 to 1,911.8)	568.9 (313.3 to 882.1)	400.7 (92.4 to 881.0)	340.3 (187.8 to 527.2)
Nutritional deficiencies	1,857.7 (1,397.0 to 2,477.0)	1,161.4 (825.0 to 1,611.0)	1,245.2 (937.3 to 1,654.4)	886.3 (630.8 to 1,230.6)
Protein-energy malnutrition	897.0 (631.9 to 1,346.2)	252.9 (194.6 to 321.6)	524.4 (377.1 to 775.4)	179.1 (139.0 to 222.8)
Iodine deficiency	19.9 (11.3 to 32.9)	10.3 (4.7 to 20.1)	22.7 (13.1 to 38.4)	12.5 (5.8 to 24.3)
Vitamin A deficiency	61.9 (41.4 to 88.6)	24.5 (16.2 to 35.5)	39.7 (26.5 to 56.4)	17.5 (11.7 to 25.4)
Dietary iron deficiency	835.7 (553.6 to 1,216.0)	849.8 (557.9 to 1,254.3)	626.3 (414.2 to 928.1)	656.2 (435.4 to 966.5)
Other nutritional deficiencies	43.2 (25.2 to 75.2)	23.9 (17.7 to 31.8)	32.1 (21.9 to 51.2)	21.0 (15.6 to 27.6)
Non-communicable diseases	17,668.5 (13,715.8 to 22,260.7)	14,453.7 (11,739.7 to 17,215.0)	23,844.5 (19,905.9 to 28,036.2)	21,141.6 (17,769.5 to 24,745.7)
Neoplasms	1,153.0 (873.2 to 1,455.3)	1,176.0 (891.6 to 1,535.3)	1,964.1 (1,486.5 to 2,439.2)	2,270.5 (1,740.7 to 2,895.4)
Lip and oral cavity cancer	7.9 (5.8 to 10.5)	8.1 (6.1 to 10.5)	15.9 (11.8 to 20.9)	17.2 (13.3 to 21.6)
Nasopharynx cancer	15.5 (11.0 to 21.3)	15.0 (10.9 to 19.8)	27.5 (19.9 to 37.3)	26.2 (19.3 to 34.0)
Other pharynx cancer	2.3 (1.6 to 3.2)	2.3 (1.7 to 3.2)	4.6 (3.4 to 6.4)	5.0 (3.8 to 6.7)
Esophageal cancer	10.8 (6.5 to 21.6)	9.3 (6.2 to 17.7)	23.2 (14.1 to 45.5)	21.9 (14.9 to 40.0)
Stomach cancer	46.1 (35.1 to 58.1)	35.0 (26.9 to 44.7)	101.0 (78.1 to 125.9)	85.7 (67.6 to 108.1)
Colon and rectum cancer	61.2 (44.7 to 80.3)	73.2 (54.2 to 95.5)	132.9 (98.5 to 171.4)	174.7 (132.5 to 224.0)
Liver cancer	45.9 (34.8 to 59.1)	39.3 (29.8 to 51.0)	84.4 (63.6 to 107.8)	81.2 (63.2 to 103.9)
Liver cancer due to hepatitis B	18.2 (13.4 to 24.4)	15.5 (11.3 to 20.6)	35.7 (26.3 to 47.5)	32.3 (23.9 to 42.6)

Liver cancer due to hepatitis C	6.9 (4.8 to 9.2)	6.3 (4.6 to 8.4)	16.1 (11.5 to 21.7)	16.4 (12.3 to 21.4)
Liver cancer due to alcohol use	7.7 (5.3 to 10.6)	6.9 (5.0 to 9.3)	17.2 (11.8 to 23.5)	17.1 (12.5 to 22.7)
Liver cancer due to NASH	3.2 (2.3 to 4.4)	3.3 (2.4 to 4.4)	7.0 (5.1 to 9.3)	7.8 (5.8 to 10.3)
Liver cancer due to other causes	9.9 (6.9 to 15.0)	7.4 (4.3 to 10.4)	8.4 (6.1 to 11.8)	7.6 (5.2 to 10.1)
Gallbladder and biliary tract cancer	7.3 (5.1 to 11.3)	6.7 (4.9 to 10.5)	16.4 (11.5 to 25.5)	16.7 (12.4 to 26.7)
Pancreatic cancer	25.2 (17.6 to 34.3)	36.3 (27.2 to 46.0)	55.6 (39.1 to 75.1)	88.8 (67.6 to 111.4)
Larynx cancer	17.7 (11.9 to 24.5)	13.0 (9.1 to 18.7)	36.6 (24.5 to 49.9)	28.6 (20.5 to 40.0)
Tracheal, bronchus, and lung cancer	70.2 (48.9 to 98.8)	70.3 (52.6 to 93.5)	155.0 (110.2 to 216.2)	172.7 (131.6 to 228.6)
Malignant skin melanoma	7.3 (3.6 to 12.0)	7.1 (4.2 to 10.3)	12.1 (6.3 to 19.3)	12.3 (7.2 to 17.5)
Non-melanoma skin cancer	2.6 (1.8 to 3.4)	3.2 (2.3 to 4.4)	5.4 (3.8 to 7.1)	7.1 (5.1 to 9.5)
Non-melanoma skin cancer (squamous-cell carcinoma)	2.6 (1.8 to 3.4)	3.2 (2.3 to 4.4)	5.4 (3.8 to 7.1)	7.1 (5.1 to 9.5)
Non-melanoma skin cancer (basal-cell carcinoma)	0.0 (0.0 to 0.0)	0.0 (0.0 to 0.0)	0.0 (0.0 to 0.0)	0.0 (0.0 to 0.0)
Breast cancer	138.3 (90.7 to 198.3)	203.4 (141.2 to 289.4)	262.7 (176.1 to 370.3)	398.5 (281.2 to 550.4)
Cervical cancer	104.3 (64.3 to 161.2)	104.9 (68.8 to 154.6)	184.4 (115.7 to 284.5)	191.7 (127.3 to 277.2)
Uterine cancer	3.4 (2.0 to 7.6)	4.1 (2.5 to 8.7)	7.3 (4.3 to 16.8)	9.6 (5.9 to 20.6)
Ovarian cancer	13.6 (8.2 to 22.4)	24.4 (15.9 to 36.9)	25.7 (15.8 to 42.0)	48.4 (31.5 to 72.2)
Prostate cancer	129.0 (56.1 to 198.1)	126.8 (57.7 to 194.2)	328.5 (145.5 to 500.3)	395.4 (182.6 to 597.3)
Testicular cancer	1.9 (0.8 to 6.4)	1.4 (0.7 to 3.8)	1.4 (0.6 to 4.0)	1.0 (0.5 to 2.6)

Kidney cancer	24.0 (10.5 to 38.7)	19.6 (12.1 to 29.8)	23.8 (14.0 to 35.3)	25.3 (17.9 to 34.9)
Bladder cancer	8.4 (6.0 to 11.1)	7.9 (6.1 to 10.3)	19.9 (14.4 to 26.0)	21.4 (16.9 to 27.3)
Brain and central nervous system cancer	58.3 (29.6 to 100.9)	57.8 (38.0 to 87.4)	58.2 (32.0 to 90.3)	63.8 (41.5 to 93.8)
Thyroid cancer	2.5 (1.9 to 3.2)	2.4 (1.8 to 3.1)	5.0 (3.8 to 6.2)	5.1 (3.7 to 6.4)
Mesothelioma	1.4 (0.6 to 2.7)	1.2 (0.7 to 2.1)	2.6 (1.2 to 5.1)	2.3 (1.4 to 4.1)
Hodgkin lymphoma	33.6 (13.1 to 52.4)	36.2 (11.7 to 62.4)	43.6 (16.3 to 67.6)	43.5 (14.3 to 72.9)
Non-Hodgkin lymphoma	43.1 (23.9 to 62.4)	43.3 (30.0 to 59.8)	49.5 (32.9 to 66.7)	55.8 (40.8 to 73.6)
Multiple myeloma	8.7 (6.3 to 12.0)	9.1 (6.8 to 11.9)	18.8 (13.7 to 25.7)	21.2 (15.9 to 27.4)
Leukemia	101.5 (54.3 to 163.1)	82.7 (57.3 to 116.6)	105.4 (67.9 to 149.2)	97.0 (72.2 to 128.4)
Acute lymphoid leukemia	24.1 (11.8 to 43.7)	20.2 (13.2 to 29.5)	16.9 (8.9 to 28.7)	15.4 (10.4 to 22.2)
Chronic lymphoid leukemia	5.1 (3.5 to 6.5)	5.6 (4.0 to 7.2)	11.6 (8.1 to 14.9)	14.4 (10.2 to 18.2)
Acute myeloid leukemia	24.5 (11.7 to 51.5)	22.2 (13.0 to 39.3)	19.0 (10.2 to 38.9)	19.3 (11.9 to 32.8)
Chronic myeloid leukemia	11.2 (6.5 to 17.5)	9.1 (6.6 to 12.1)	13.4 (9.1 to 18.6)	12.5 (9.2 to 16.7)
Other leukemia	36.6 (16.9 to 60.2)	25.6 (17.5 to 35.6)	44.5 (25.0 to 63.5)	35.4 (26.2 to 47.2)
Other malignant neoplasms	157.9 (113.3 to 209.6)	128.0 (93.1 to 168.1)	150.8 (114.3 to 192.6)	144.4 (109.1 to 186.1)
Other neoplasms	3.1 (2.1 to 4.1)	3.7 (2.8 to 4.9)	6.2 (4.0 to 8.1)	8.3 (6.6 to 10.7)
Myelodysplastic, myeloproliferative, and other hematopoietic neoplasms	3.1 (2.1 to 4.1)	3.7 (2.8 to 4.9)	6.2 (4.0 to 8.1)	8.3 (6.6 to 10.7)
Benign and in situ intestinal neoplasms	0.0 (0.0 to 0.0)	0.0 (0.0 to 0.0)	0.0 (0.0 to 0.0)	0.0 (0.0 to 0.0)

Benign and in situ cervical and uterine neoplasms	0.0 (0.0 to 0.0)	0.0 (0.0 to 0.0)	0.0 (0.0 to 0.0)	0.0 (0.0 to 0.0)
Other benign and in situ neoplasms	0.0 (0.0 to 0.0)	0.0 (0.0 to 0.0)	0.0 (0.0 to 0.0)	0.0 (0.0 to 0.0)
Cardiovascular diseases	3,127.0 (2,374.0 to 4,250.6)	2,040.4 (1,635.3 to 2,516.0)	6,464.5 (4,861.1 to 8,815.1)	4,675.0 (3,792.5 to 5,620.2)
Rheumatic heart disease	137.2 (94.5 to 190.9)	77.0 (55.3 to 108.4)	175.0 (119.8 to 248.0)	96.3 (70.9 to 133.4)
Ischemic heart disease	1,013.1 (710.3 to 1,466.0)	701.6 (506.6 to 928.7)	2,357.4 (1,693.7 to 3,333.7)	1,840.1 (1,339.1 to 2,387.7)
Stroke	1,269.4 (959.9 to 1,735.4)	794.2 (633.4 to 995.5)	2,670.9 (2,015.8 to 3,687.5)	1,827.1 (1,478.7 to 2,257.0)
Ischemic stroke	338.5 (263.4 to 477.1)	244.5 (199.4 to 300.9)	823.7 (635.6 to 1,169.6)	658.0 (538.7 to 794.3)
Intracerebral hemorrhage	881.3 (646.7 to 1,218.4)	519.6 (401.9 to 678.7)	1,776.0 (1,303.6 to 2,453.1)	1,124.4 (877.0 to 1,450.2)
Subarachnoid hemorrhage	49.6 (24.4 to 102.9)	30.1 (19.7 to 59.2)	71.2 (37.9 to 155.5)	44.7 (27.4 to 85.0)
Hypertensive heart disease	199.6 (123.1 to 306.7)	139.2 (88.1 to 191.2)	444.0 (283.5 to 662.1)	334.1 (214.8 to 445.2)
Non-rheumatic valvular heart disease	19.0 (10.0 to 31.8)	13.8 (9.6 to 19.3)	33.2 (18.2 to 53.8)	24.2 (17.3 to 33.1)
Non-rheumatic calcific aortic valve disease	9.0 (4.3 to 16.0)	6.0 (3.9 to 8.9)	17.0 (8.3 to 28.8)	11.7 (7.9 to 17.0)
Non-rheumatic degenerative mitral valve disease	9.7 (5.3 to 15.9)	7.7 (5.3 to 10.8)	16.0 (9.3 to 24.9)	12.4 (8.7 to 16.7)
Other non-rheumatic valve diseases	0.2 (0.1 to 0.4)	0.1 (0.1 to 0.2)	0.3 (0.1 to 0.5)	0.2 (0.1 to 0.3)
Cardiomyopathy and myocarditis	135.0 (72.1 to 204.9)	76.8 (54.4 to 103.4)	226.4 (127.8 to 338.4)	137.1 (100.7 to 177.9)
Myocarditis	8.2 (3.3 to 15.5)	5.6 (3.5 to 8.6)	9.9 (4.9 to 16.6)	6.7 (4.6 to 9.5)
Alcoholic cardiomyopathy	11.1 (5.8 to 18.8)	3.0 (1.8 to 4.8)	20.4 (10.7 to 35.1)	5.5 (3.3 to 8.7)
Other cardiomyopathy	115.7 (61.7 to 174.3)	68.2 (48.5 to 91.7)	196.0 (111.2 to 291.6)	124.9 (92.0 to 161.4)

Atrial fibrillation and flutter	30.8 (23.6 to 39.4)	26.0 (20.2 to 32.2)	92.2 (71.7 to 117.8)	87.7 (68.7 to 107.8)
Aortic aneurysm	19.7 (12.3 to 31.2)	12.5 (9.0 to 17.1)	43.7 (28.1 to 68.9)	30.4 (22.6 to 40.6)
Peripheral artery disease	3.5 (2.4 to 5.2)	3.4 (2.0 to 5.1)	9.6 (6.6 to 13.9)	10.5 (6.4 to 15.7)
Endocarditis	46.7 (21.4 to 70.4)	22.6 (16.0 to 33.1)	42.6 (24.2 to 65.6)	23.7 (16.6 to 33.3)
Other cardiovascular and circulatory diseases	252.9 (193.0 to 326.5)	173.3 (135.7 to 220.7)	369.4 (282.3 to 502.6)	263.8 (207.6 to 330.1)
Chronic respiratory diseases	736.1 (610.6 to 883.1)	547.5 (452.7 to 651.4)	1,225.4 (1,007.7 to 1,454.8)	966.6 (805.0 to 1,134.9)
Chronic obstructive pulmonary disease	287.4 (238.6 to 342.8)	223.2 (185.2 to 263.6)	607.1 (499.2 to 724.9)	523.0 (430.7 to 623.3)
Pneumoconiosis	0.6 (0.3 to 0.8)	0.6 (0.4 to 0.8)	1.1 (0.7 to 1.6)	1.1 (0.7 to 1.5)
Silicosis	0.2 (0.1 to 0.3)	0.2 (0.1 to 0.3)	0.4 (0.1 to 0.6)	0.3 (0.2 to 0.5)
Asbestosis	0.1 (0.1 to 0.2)	0.1 (0.1 to 0.1)	0.2 (0.1 to 0.3)	0.2 (0.1 to 0.3)
Coal workers pneumoconiosis	0.0 (0.0 to 0.1)	0.0 (0.0 to 0.1)	0.1 (0.0 to 0.2)	0.1 (0.0 to 0.1)
Other pneumoconiosis	0.2 (0.1 to 0.3)	0.3 (0.2 to 0.5)	0.4 (0.3 to 0.6)	0.5 (0.4 to 0.8)
Asthma	393.1 (309.8 to 492.9)	266.8 (201.0 to 344.5)	550.0 (435.6 to 678.0)	375.8 (297.4 to 460.3)
Interstitial lung disease and pulmonary sarcoidosis	9.6 (5.1 to 15.5)	7.5 (5.2 to 10.5)	19.8 (10.0 to 32.8)	15.9 (10.7 to 22.0)
Other chronic respiratory diseases	45.4 (30.7 to 70.6)	49.4 (37.5 to 64.5)	47.4 (33.6 to 67.5)	50.9 (39.8 to 65.6)
Digestive diseases	1,757.8 (1,356.9 to 2,267.1)	1,386.0 (902.7 to 2,070.7)	2,537.9 (1,844.2 to 3,429.7)	2,116.1 (1,392.6 to 3,131.1)
Cirrhosis and other chronic liver diseases	848.0 (608.5 to 1,138.6)	679.7 (442.8 to 1,033.9)	1,406.8 (982.1 to 1,933.5)	1,152.5 (765.4 to 1,726.9)
Cirrhosis and other chronic liver diseases due to hepatitis B	372.2 (251.9 to 522.5)	277.3 (175.3 to 428.8)	673.5 (458.8 to 938.8)	511.4 (329.3 to 771.7)

Cirrhosis and other chronic liver diseases due to hepatitis C	37.0 (24.5 to 53.8)	32.1 (20.0 to 51.5)	67.6 (45.0 to 98.9)	59.3 (38.1 to 92.5)
Cirrhosis and other chronic liver diseases due to alcohol use	152.7 (97.5 to 229.0)	122.5 (76.0 to 192.6)	305.5 (194.7 to 460.5)	253.8 (160.9 to 394.1)
Cirrhosis and other chronic liver diseases due to NAFLD	47.0 (28.0 to 75.0)	41.5 (23.6 to 70.1)	93.2 (56.6 to 149.4)	85.5 (50.0 to 139.3)
Cirrhosis and other chronic liver diseases due to other causes	239.1 (174.8 to 322.2)	206.3 (135.6 to 311.2)	266.9 (193.7 to 357.9)	242.5 (160.5 to 369.5)
Upper digestive system diseases	262.5 (175.6 to 390.6)	201.7 (125.2 to 314.4)	377.0 (251.4 to 557.2)	301.5 (191.2 to 457.2)
Peptic ulcer disease	157.5 (95.8 to 264.3)	108.5 (59.3 to 200.1)	236.7 (141.8 to 384.9)	171.1 (97.1 to 301.7)
Gastritis and duodenitis	45.8 (28.6 to 68.6)	34.2 (22.6 to 51.4)	52.7 (33.8 to 81.2)	42.2 (28.5 to 63.8)
Gastroesophageal reflux disease	59.3 (30.8 to 104.7)	58.9 (30.7 to 104.5)	87.6 (44.9 to 157.1)	88.2 (45.3 to 158.5)
Appendicitis	46.3 (20.0 to 75.4)	18.5 (9.9 to 34.1)	40.8 (21.3 to 64.5)	18.5 (10.4 to 32.2)
Paralytic ileus and intestinal obstruction	273.2 (180.3 to 398.7)	244.2 (124.3 to 442.6)	271.9 (159.2 to 423.3)	277.4 (132.8 to 500.7)
Inguinal, femoral, and abdominal hernia	71.4 (47.0 to 93.2)	54.5 (38.2 to 75.1)	82.2 (59.7 to 118.6)	69.8 (47.7 to 105.6)
Inflammatory bowel disease	16.1 (6.6 to 24.9)	12.5 (7.0 to 19.8)	19.5 (10.3 to 30.4)	17.1 (10.2 to 26.7)
Vascular intestinal disorders	18.1 (12.1 to 26.2)	13.3 (7.7 to 22.2)	23.2 (16.5 to 32.7)	22.8 (13.1 to 40.0)
Gallbladder and biliary diseases	46.8 (25.0 to 62.5)	41.1 (21.3 to 68.8)	66.1 (41.4 to 95.8)	68.7 (35.9 to 127.6)
Pancreatitis	81.9 (45.6 to 144.1)	69.3 (36.9 to 132.9)	132.6 (74.0 to 239.7)	114.2 (61.8 to 220.5)
Other digestive diseases	93.6 (43.5 to 151.1)	51.3 (31.9 to 72.7)	117.6 (58.5 to 162.5)	73.6 (45.9 to 112.7)
Neurological disorders	1,085.5 (604.5 to 1,784.4)	1,053.3 (566.9 to 1,739.5)	1,413.2 (816.9 to 2,273.3)	1,443.2 (848.0 to 2,303.2)

Alzheimer's disease and other dementias	83.3 (33.5 to 203.2)	84.1 (32.5 to 198.1)	286.9 (112.3 to 706.2)	326.6 (123.9 to 780.6)
Parkinson's disease	24.0 (19.8 to 29.6)	25.2 (20.3 to 30.3)	69.9 (58.0 to 84.9)	87.0 (70.7 to 103.7)
Idiopathic epilepsy	350.5 (257.7 to 471.5)	297.5 (209.8 to 446.2)	352.4 (256.5 to 477.6)	308.0 (218.5 to 465.3)
Multiple sclerosis	3.8 (2.8 to 5.5)	4.9 (3.6 to 6.8)	6.2 (4.6 to 9.0)	7.9 (5.7 to 11.2)
Motor neuron disease	1.2 (0.9 to 1.5)	1.3 (1.0 to 1.7)	1.7 (1.2 to 2.3)	1.9 (1.4 to 2.5)
Headache disorders	540.6 (97.4 to 1,195.4)	551.0 (100.2 to 1,204.5)	627.1 (130.5 to 1,345.2)	635.2 (133.5 to 1,361.6)
Migraine	494.0 (63.7 to 1,135.1)	502.8 (65.7 to 1,163.8)	567.6 (85.2 to 1,290.7)	574.0 (87.5 to 1,288.3)
Tension-type headache	46.6 (13.7 to 177.6)	48.3 (14.1 to 179.8)	59.5 (18.7 to 206.6)	61.2 (19.4 to 207.8)
Other neurological disorders	82.1 (58.8 to 111.0)	89.4 (63.0 to 122.3)	69.0 (50.4 to 92.6)	76.6 (54.7 to 102.9)
Mental disorders	1,321.0 (964.4 to 1,748.4)	1,214.5 (883.9 to 1,614.2)	1,604.7 (1,185.7 to 2,107.9)	1,472.7 (1,086.1 to 1,941.2)
Schizophrenia	103.1 (75.0 to 131.5)	105.5 (77.0 to 134.8)	138.6 (100.7 to 175.0)	142.5 (103.4 to 180.4)
Depressive disorders	577.1 (393.1 to 795.8)	452.7 (309.0 to 618.9)	782.4 (540.8 to 1,073.5)	639.6 (446.6 to 873.4)
Major depressive disorder	480.4 (323.8 to 674.5)	354.0 (238.8 to 497.8)	655.3 (445.6 to 916.3)	509.7 (348.2 to 710.6)
Dysthymia	96.8 (63.0 to 144.0)	98.6 (63.8 to 147.2)	127.2 (83.5 to 189.6)	129.9 (85.3 to 194.4)
Bipolar disorder	96.1 (59.2 to 147.9)	97.2 (59.5 to 150.1)	118.7 (74.0 to 181.8)	119.3 (74.2 to 181.6)
Anxiety disorders	252.5 (175.5 to 349.7)	263.4 (183.1 to 361.9)	273.9 (193.2 to 376.0)	285.8 (201.0 to 391.6)
Eating disorders	20.7 (12.7 to 30.8)	28.1 (17.3 to 42.1)	20.2 (12.5 to 30.0)	27.6 (17.1 to 41.3)

Anorexia nervosa	6.7 (3.9 to 10.7)	9.0 (5.3 to 14.6)	6.2 (3.6 to 9.7)	8.1 (4.8 to 12.9)
Bulimia nervosa	13.9 (8.1 to 22.0)	19.1 (11.2 to 30.1)	14.1 (8.1 to 22.2)	19.4 (11.1 to 30.5)
Autism spectrum disorders	61.1 (40.1 to 88.1)	60.3 (39.5 to 87.6)	57.4 (37.7 to 82.6)	55.9 (36.8 to 81.2)
Attention-deficit/hyperactivity disorder	8.7 (4.8 to 15.4)	9.4 (5.1 to 16.5)	7.2 (4.1 to 12.8)	7.4 (4.0 to 12.7)
Conduct disorder	94.8 (53.7 to 150.2)	105.7 (59.4 to 166.4)	71.4 (40.6 to 113.0)	72.7 (40.6 to 114.4)
Idiopathic developmental intellectual disability	38.6 (17.1 to 66.1)	25.3 (10.3 to 44.8)	33.5 (14.6 to 57.8)	22.4 (9.1 to 39.8)
Other mental disorders	68.3 (43.7 to 104.0)	66.8 (42.5 to 101.9)	101.3 (65.4 to 154.4)	99.6 (63.9 to 151.7)
Substance use disorders	120.0 (90.4 to 152.7)	122.1 (90.8 to 155.7)	159.0 (122.0 to 199.7)	161.1 (123.6 to 203.6)
Alcohol use disorders	60.7 (46.0 to 77.6)	57.1 (42.0 to 75.6)	89.9 (69.3 to 113.5)	84.1 (63.5 to 109.3)
Drug use disorders	59.3 (41.8 to 79.6)	65.0 (45.9 to 86.0)	69.1 (49.4 to 91.4)	77.0 (55.3 to 101.2)
Opioid use disorders	43.6 (29.4 to 60.7)	48.5 (33.1 to 67.0)	51.4 (35.1 to 69.3)	58.1 (40.2 to 78.5)
Cocaine use disorders	0.9 (0.6 to 1.4)	1.0 (0.6 to 1.5)	1.3 (0.8 to 1.9)	1.5 (1.0 to 2.2)
Amphetamine use disorders	6.0 (3.3 to 9.9)	6.1 (3.4 to 9.9)	6.4 (3.6 to 10.5)	6.7 (3.8 to 10.9)
Cannabis use disorders	3.3 (1.9 to 5.3)	3.4 (2.0 to 5.6)	3.5 (2.0 to 5.4)	3.5 (2.1 to 5.5)
Other drug use disorders	5.5 (3.5 to 8.2)	5.9 (3.8 to 8.7)	6.6 (4.4 to 9.6)	7.2 (4.9 to 10.3)
Diabetes and kidney diseases	867.7 (706.3 to 1,059.7)	750.3 (604.5 to 908.1)	1,576.5 (1,296.3 to 1,919.1)	1,531.1 (1,240.1 to 1,824.4)
Diabetes mellitus	426.7 (349.8 to 512.5)	392.2 (314.3 to 476.8)	908.8 (752.3 to 1,083.9)	917.8 (745.8 to 1,099.3)
Diabetes mellitus type 1	52.2 (36.9 to 74.0)	49.2 (36.7 to 63.3)	69.3 (45.6 to 106.6)	66.6 (47.1 to 89.3)

Diabetes mellitus type 2	374.5 (304.7 to 450.9)	343.0 (272.4 to 418.8)	839.5 (691.6 to 999.4)	851.2 (691.2 to 1,029.1)
Chronic kidney disease	439.7 (345.7 to 564.9)	357.4 (284.3 to 447.5)	666.8 (524.9 to 856.8)	612.7 (495.4 to 755.2)
Chronic kidney disease due to diabetes mellitus type 1	23.1 (13.9 to 35.6)	21.5 (13.9 to 32.6)	36.7 (21.8 to 57.7)	33.9 (21.6 to 51.4)
Chronic kidney disease due to diabetes mellitus type 2	53.2 (36.5 to 74.6)	45.6 (32.7 to 61.1)	125.0 (86.6 to 172.0)	118.7 (86.4 to 156.8)
Chronic kidney disease due to hypertension	95.4 (71.7 to 129.7)	74.6 (56.8 to 96.8)	212.1 (161.6 to 284.9)	181.0 (141.8 to 229.8)
Chronic kidney disease due to glomerulonephritis	159.2 (114.1 to 217.8)	119.8 (86.6 to 160.4)	171.5 (126.9 to 234.6)	152.4 (111.9 to 203.9)
Chronic kidney disease due to other and unspecified causes	108.9 (80.6 to 142.9)	95.9 (73.7 to 121.7)	121.6 (94.5 to 157.6)	126.7 (98.3 to 159.9)
Acute glomerulonephritis	1.3 (0.2 to 3.6)	0.7 (0.2 to 1.6)	0.9 (0.2 to 2.3)	0.5 (0.2 to 1.2)
Skin and subcutaneous diseases	540.8 (358.3 to 840.0)	545.4 (356.0 to 848.7)	526.7 (348.3 to 820.6)	529.1 (349.2 to 830.9)
Dermatitis	86.6 (51.8 to 135.1)	87.4 (51.9 to 137.1)	82.6 (50.4 to 126.1)	83.2 (50.6 to 127.6)
Atopic dermatitis	60.2 (32.6 to 101.1)	61.1 (32.8 to 102.4)	48.1 (26.0 to 81.1)	48.6 (26.0 to 81.9)
Contact dermatitis	21.1 (13.2 to 31.7)	21.0 (13.3 to 32.1)	28.6 (17.9 to 42.7)	28.7 (17.9 to 42.5)
Seborrhoeic dermatitis	5.3 (3.0 to 8.4)	5.3 (3.0 to 8.5)	5.9 (3.3 to 9.3)	5.9 (3.4 to 9.4)
Psoriasis	22.5 (15.9 to 30.0)	16.9 (11.9 to 22.6)	27.3 (19.4 to 36.2)	20.3 (14.3 to 26.8)
Bacterial skin diseases	41.2 (30.5 to 58.4)	33.9 (24.8 to 45.2)	46.4 (36.1 to 59.5)	43.0 (31.8 to 55.9)
Cellulitis	3.1 (1.3 to 5.5)	2.4 (1.3 to 3.7)	3.9 (1.8 to 5.8)	3.5 (1.8 to 5.1)
Pyoderma	38.1 (27.5 to 55.1)	31.4 (22.6 to 42.9)	42.5 (32.6 to 55.0)	39.6 (29.0 to 51.5)

Scabies	41.4 (22.4 to 66.2)	38.5 (20.8 to 61.8)	38.3 (20.8 to 61.2)	36.4 (20.0 to 57.9)
Fungal skin diseases	107.9 (43.8 to 222.6)	107.8 (43.8 to 221.1)	112.0 (45.2 to 231.8)	111.5 (44.9 to 229.6)
Viral skin diseases	67.1 (42.5 to 99.1)	68.5 (43.4 to 101.3)	51.9 (33.1 to 76.5)	52.1 (33.3 to 77.0)
Acne vulgaris	69.6 (41.8 to 109.9)	86.5 (52.1 to 136.8)	58.2 (35.1 to 91.8)	67.9 (41.0 to 107.5)
Alopecia areata	5.8 (3.7 to 8.7)	6.0 (3.7 to 8.9)	6.6 (4.2 to 9.8)	6.8 (4.3 to 10.3)
Pruritus	8.3 (3.9 to 14.8)	8.8 (4.1 to 15.7)	9.6 (4.6 to 17.1)	10.2 (4.9 to 18.1)
Urticaria	58.1 (37.9 to 84.8)	58.0 (37.9 to 84.6)	50.1 (32.7 to 72.1)	50.8 (33.4 to 73.3)
Decubitus ulcer	0.8 (0.6 to 1.0)	1.1 (0.7 to 1.5)	1.9 (1.4 to 2.5)	3.5 (2.0 to 4.9)
Other skin and subcutaneous diseases	31.6 (15.5 to 56.8)	32.0 (15.4 to 58.0)	41.7 (20.5 to 74.7)	43.3 (21.2 to 78.0)
Sense organ diseases	709.0 (489.0 to 975.4)	635.1 (436.7 to 878.8)	1,181.1 (834.3 to 1,616.4)	1,108.8 (779.4 to 1,520.1)
Blindness and vision loss	239.0 (168.0 to 332.5)	206.7 (143.9 to 290.3)	506.0 (356.8 to 698.7)	467.8 (329.6 to 646.7)
Glaucoma	14.8 (10.1 to 20.8)	10.8 (7.4 to 15.1)	41.4 (28.7 to 58.2)	35.2 (24.2 to 49.1)
Cataract	94.8 (67.1 to 129.9)	76.3 (53.8 to 104.4)	229.6 (162.9 to 311.0)	204.8 (145.4 to 276.1)
Age-related macular degeneration	8.5 (5.9 to 11.7)	6.7 (4.6 to 9.3)	21.3 (14.7 to 29.4)	19.2 (13.3 to 26.7)
Refraction disorders	42.4 (28.8 to 60.3)	42.6 (28.7 to 61.1)	60.3 (42.0 to 83.7)	61.4 (42.5 to 85.4)
Near vision loss	49.4 (22.1 to 98.7)	43.4 (19.2 to 86.5)	106.6 (48.4 to 212.0)	99.2 (45.4 to 196.6)
Other vision loss	29.1 (20.0 to 40.5)	26.9 (18.5 to 37.9)	46.7 (32.4 to 64.7)	48.1 (33.6 to 66.2)

Age-related and other hearing loss	443.0 (296.6 to 613.1)	401.1 (268.5 to 561.2)	634.7 (434.7 to 876.9)	598.9 (411.0 to 830.5)
Other sense organ diseases	27.0 (16.5 to 40.9)	27.3 (16.5 to 41.6)	40.4 (24.0 to 62.5)	42.1 (25.1 to 65.0)
Musculoskeletal disorders	963.8 (692.3 to 1,275.9)	985.0 (702.6 to 1,301.4)	1,505.4 (1,075.0 to 2,000.8)	1,561.3 (1,115.8 to 2,072.7)
Rheumatoid arthritis	5.5 (4.1 to 7.1)	6.6 (4.7 to 8.7)	10.3 (7.7 to 13.2)	13.2 (9.3 to 17.3)
Osteoarthritis	94.9 (48.2 to 186.9)	92.2 (46.9 to 183.0)	206.8 (105.2 to 405.5)	210.8 (107.3 to 414.6)
Osteoarthritis hip	4.7 (2.2 to 9.7)	4.0 (1.9 to 8.3)	10.1 (4.7 to 21.0)	9.2 (4.2 to 19.2)
Osteoarthritis knee	55.4 (27.4 to 112.5)	54.5 (27.0 to 111.2)	119.6 (59.2 to 242.7)	123.2 (61.0 to 250.8)
Osteoarthritis hand	24.4 (12.1 to 50.9)	24.2 (12.0 to 50.8)	53.2 (26.4 to 110.3)	55.1 (27.4 to 115.1)
Osteoarthritis other	10.4 (5.1 to 21.4)	9.5 (4.6 to 19.5)	23.8 (11.6 to 49.3)	23.4 (11.4 to 48.2)
Low back pain	605.3 (427.1 to 807.4)	592.8 (417.9 to 796.3)	894.2 (634.5 to 1,197.8)	881.2 (619.2 to 1,184.7)
Neck pain	91.8 (59.8 to 133.0)	95.6 (62.0 to 139.5)	140.2 (92.7 to 202.2)	147.8 (96.3 to 213.1)
Gout	8.0 (5.0 to 11.5)	6.5 (4.1 to 9.4)	15.9 (9.9 to 23.1)	13.5 (8.5 to 19.4)
Other musculoskeletal disorders	158.3 (108.8 to 221.6)	191.2 (130.5 to 265.8)	238.1 (163.1 to 333.2)	294.9 (203.1 to 407.8)
Other non-communicable diseases	5,286.6 (2,521.9 to 9,056.1)	3,998.0 (2,364.7 to 5,874.2)	3,686.1 (2,090.7 to 5,655.8)	3,306.2 (2,134.8 to 4,556.9)
Congenital birth defects	3,156.7 (1,113.2 to 6,794.8)	2,265.6 (1,007.5 to 3,930.1)	1,667.7 (611.7 to 3,515.4)	1,437.9 (657.5 to 2,469.8)
Neural tube defects	1,057.6 (422.4 to 2,278.3)	707.6 (333.6 to 1,366.6)	534.0 (208.3 to 1,161.1)	437.4 (205.1 to 846.4)
Congenital heart anomalies	614.6 (136.0 to 1,484.2)	466.7 (158.2 to 1,018.7)	319.6 (75.4 to 772.5)	292.8 (101.8 to 635.0)
Orofacial clefts	22.7 (6.0 to 75.1)	12.4 (5.5 to 27.7)	12.1 (4.0 to 36.5)	8.5 (4.3 to 17.9)

Down syndrome	82.0 (17.8 to 283.4)	72.4 (23.4 to 205.9)	44.8 (10.8 to 153.3)	46.9 (16.0 to 130.4)
Turner syndrome	0.2 (0.1 to 0.3)	0.2 (0.1 to 0.4)	0.1 (0.1 to 0.3)	0.2 (0.1 to 0.3)
Klinefelter syndrome	0.0 (0.0 to 0.0)	0.0 (0.0 to 0.0)	0.0 (0.0 to 0.0)	0.0 (0.0 to 0.0)
Other chromosomal abnormalities	97.5 (26.6 to 404.0)	96.8 (33.4 to 269.8)	48.9 (13.8 to 198.3)	59.9 (21.0 to 166.1)
Congenital musculoskeletal and limb anomalies	102.3 (51.0 to 184.3)	93.7 (52.8 to 157.1)	63.8 (34.5 to 108.5)	65.9 (38.5 to 106.4)
Urogenital congenital anomalies	45.9 (13.9 to 104.3)	38.8 (15.7 to 69.5)	23.0 (7.5 to 51.3)	24.1 (10.1 to 42.7)
Digestive congenital anomalies	314.2 (70.6 to 804.3)	218.2 (71.3 to 475.2)	155.3 (34.1 to 405.1)	133.7 (43.6 to 292.9)
Other congenital birth defects	819.8 (208.1 to 2,269.1)	559.0 (196.2 to 1,182.0)	465.9 (136.1 to 1,201.2)	368.5 (139.9 to 751.8)
Urinary diseases and male infertility	113.2 (91.2 to 138.6)	80.7 (62.9 to 102.5)	157.0 (127.2 to 198.7)	130.2 (103.5 to 165.6)
Urinary tract infections and interstitial nephritis	52.0 (38.5 to 71.6)	34.2 (24.6 to 48.0)	61.9 (47.0 to 83.0)	47.6 (34.8 to 67.9)
Urolithiasis	2.1 (1.4 to 2.9)	1.8 (1.3 to 2.5)	3.4 (2.3 to 4.7)	3.0 (2.1 to 4.0)
Benign prostatic hyperplasia	5.0 (2.9 to 7.9)	3.9 (2.3 to 6.0)	12.0 (6.9 to 18.6)	10.8 (6.3 to 16.7)
Male infertility	4.9 (1.8 to 11.9)	5.8 (2.1 to 13.6)	5.7 (2.1 to 13.5)	6.7 (2.4 to 15.6)
Other urinary diseases	49.2 (35.7 to 66.1)	35.0 (25.8 to 47.2)	74.1 (53.8 to 106.1)	62.2 (45.7 to 87.8)
Gynecological diseases	370.3 (252.4 to 516.4)	411.6 (281.3 to 575.4)	481.2 (330.8 to 662.4)	543.0 (370.0 to 748.7)
Uterine fibroids	12.8 (6.4 to 22.8)	13.1 (6.5 to 23.8)	17.7 (9.0 to 31.9)	18.4 (9.1 to 33.6)
Polycystic ovarian syndrome	2.4 (1.0 to 5.0)	2.8 (1.2 to 5.8)	2.6 (1.1 to 5.3)	3.0 (1.3 to 6.1)
Female infertility	6.5 (1.8 to 17.1)	9.7 (3.1 to 24.0)	7.5 (1.9 to 18.6)	11.4 (3.5 to 27.6)
Endometriosis	33.2 (19.4 to 53.7)	27.3 (16.0 to 43.8)	36.4 (21.5 to 58.2)	30.6 (18.2 to 48.4)

Genital prolapse	4.4 (2.3 to 7.7)	4.1 (2.0 to 7.5)	8.2 (4.2 to 14.9)	7.8 (3.9 to 14.5)
Premenstrual syndrome	81.3 (49.3 to 128.0)	90.0 (54.4 to 141.4)	85.8 (52.6 to 134.4)	94.8 (57.7 to 147.9)
Other gynecological diseases	229.8 (148.5 to 327.3)	264.7 (171.7 to 376.2)	323.1 (210.4 to 457.7)	377.0 (246.5 to 531.8)
Hemoglobinopathies and hemolytic anemias	1,021.6 (474.4 to 1,683.7)	679.4 (418.7 to 953.3)	733.2 (368.0 to 1,173.2)	541.0 (335.8 to 752.3)
Thalassemias	2.6 (0.6 to 6.4)	1.8 (0.6 to 4.0)	1.5 (0.3 to 3.6)	1.2 (0.4 to 2.5)
Thalassemias trait	20.7 (13.5 to 32.0)	23.3 (15.0 to 36.0)	17.8 (11.7 to 27.3)	20.5 (13.2 to 31.4)
Sickle cell disorders	802.4 (281.0 to 1,435.3)	454.7 (231.3 to 720.6)	529.5 (193.6 to 934.9)	328.1 (167.6 to 512.8)
Sickle cell trait	133.3 (87.0 to 191.1)	143.5 (92.3 to 209.3)	113.2 (74.4 to 164.2)	126.2 (81.2 to 183.9)
G6PD deficiency	15.3 (5.5 to 27.7)	12.2 (5.5 to 20.6)	17.8 (6.4 to 32.3)	15.4 (6.7 to 26.0)
G6PD trait	0.1 (0.0 to 0.1)	0.3 (0.2 to 0.4)	0.1 (0.0 to 0.1)	0.3 (0.2 to 0.4)
Other hemoglobinopathies and hemolytic anemias	47.2 (32.1 to 64.7)	43.6 (29.0 to 61.6)	53.3 (35.3 to 73.4)	49.3 (32.6 to 67.6)
Endocrine, metabolic, blood, and immune disorders	245.8 (179.0 to 324.7)	242.1 (174.8 to 324.8)	299.0 (213.7 to 396.9)	309.0 (222.0 to 415.0)
Oral disorders	182.3 (95.9 to 315.0)	186.3 (98.1 to 320.6)	253.6 (131.4 to 437.3)	264.1 (138.4 to 455.4)
Caries of deciduous teeth	3.7 (1.5 to 8.0)	3.7 (1.6 to 8.1)	2.3 (1.0 to 5.1)	2.4 (1.0 to 5.3)
Caries of permanent teeth	19.4 (8.7 to 37.4)	20.0 (9.0 to 38.6)	21.7 (9.9 to 41.4)	21.8 (9.9 to 41.5)
Periodontal diseases	93.1 (35.9 to 200.9)	93.4 (36.0 to 202.4)	138.6 (55.0 to 291.9)	139.5 (55.3 to 293.7)
Edentulism	18.7 (11.6 to 28.4)	21.2 (13.2 to 32.4)	38.1 (23.9 to 57.0)	46.9 (29.6 to 71.0)
Other oral disorders	47.4 (29.6 to 69.7)	48.0 (29.9 to 70.5)	52.9 (33.1 to 77.5)	53.5 (33.5 to 78.2)

Sudden infant death syndrome	196.8 (15.0 to 608.7)	132.2 (14.0 to 383.0)	94.4 (7.2 to 291.9)	80.9 (8.5 to 234.2)
Injuries	3,491.6 (2,879.7 to 4,118.9)	2,414.3 (1,935.5 to 2,924.3)	3,364.6 (2,769.7 to 3,984.8)	2,620.2 (2,127.5 to 3,152.4)
Transport injuries	943.1 (753.1 to 1,160.7)	657.7 (512.5 to 835.4)	956.1 (777.3 to 1,168.6)	759.1 (606.2 to 936.2)
Road injuries	868.0 (689.3 to 1,077.3)	616.5 (482.1 to 783.4)	882.4 (714.2 to 1,077.7)	716.4 (571.3 to 881.0)
Pedestrian road injuries	302.0 (214.7 to 407.8)	184.1 (136.5 to 252.5)	297.6 (225.5 to 387.4)	216.7 (169.6 to 286.3)
Cyclist road injuries	51.7 (38.7 to 73.9)	45.4 (34.9 to 58.8)	60.4 (46.9 to 78.4)	62.2 (47.7 to 78.2)
Motorcyclist road injuries	89.5 (64.3 to 134.4)	63.1 (47.4 to 90.2)	89.4 (68.2 to 127.9)	74.0 (56.2 to 105.2)
Motor vehicle road injuries	417.8 (309.8 to 530.0)	318.8 (241.1 to 415.4)	427.2 (327.6 to 535.9)	356.9 (278.3 to 454.5)
Other road injuries	7.1 (5.1 to 10.1)	5.1 (3.8 to 6.6)	7.8 (5.9 to 10.1)	6.5 (5.1 to 8.2)
Other transport injuries	75.1 (56.7 to 97.2)	41.2 (30.5 to 53.6)	73.6 (59.7 to 96.4)	42.7 (32.6 to 56.4)
Unintentional injuries	1,821.0 (1,487.2 to 2,195.4)	1,078.8 (836.8 to 1,377.9)	1,595.2 (1,326.7 to 1,940.0)	1,112.1 (879.1 to 1,383.9)
Falls	183.1 (151.7 to 220.2)	167.6 (129.4 to 212.8)	269.8 (219.1 to 328.7)	268.1 (210.3 to 334.2)
Drowning	240.0 (177.7 to 318.5)	114.0 (80.0 to 165.9)	155.9 (116.7 to 214.9)	83.0 (58.9 to 121.1)
Fire, heat, and hot substances	212.8 (142.0 to 300.2)	129.6 (92.0 to 186.4)	169.5 (122.5 to 231.4)	121.5 (88.9 to 170.5)
Poisonings	208.1 (158.9 to 266.4)	113.8 (83.6 to 167.5)	158.9 (121.1 to 220.6)	99.4 (74.2 to 145.3)
Poisoning by carbon monoxide	33.1 (19.4 to 66.7)	27.0 (15.9 to 56.8)	34.7 (21.0 to 69.4)	29.4 (18.5 to 56.9)
Poisoning by other means	175.0 (123.3 to 217.2)	86.9 (65.2 to 115.4)	124.3 (93.3 to 158.0)	70.0 (52.3 to 93.0)

Exposure to mechanical forces	262.4 (199.2 to 347.0)	129.3 (89.4 to 177.8)	236.1 (186.8 to 305.2)	130.5 (94.8 to 172.1)
Unintentional firearm injuries	70.2 (43.1 to 102.0)	33.1 (19.9 to 50.1)	62.8 (40.8 to 91.0)	33.9 (21.1 to 49.0)
Other exposure to mechanical forces	192.2 (145.0 to 259.5)	96.3 (66.7 to 132.7)	173.3 (136.4 to 234.0)	96.6 (69.6 to 127.4)
Adverse effects of medical treatment	217.4 (125.7 to 296.8)	110.8 (71.7 to 155.2)	167.5 (108.4 to 220.1)	100.4 (71.6 to 133.8)
Animal contact	156.8 (109.7 to 204.8)	94.2 (65.5 to 142.5)	139.1 (101.5 to 189.3)	92.4 (65.9 to 137.6)
Venomous animal contact	95.5 (63.4 to 135.8)	57.9 (37.0 to 107.2)	92.2 (63.6 to 138.0)	60.9 (41.2 to 104.9)
Non-venomous animal contact	61.4 (28.0 to 88.4)	36.3 (23.3 to 51.0)	46.9 (26.4 to 65.5)	31.5 (22.4 to 43.1)
Foreign body	143.1 (117.6 to 174.9)	105.0 (83.2 to 127.4)	120.4 (101.0 to 144.3)	101.8 (82.4 to 121.4)
Pulmonary aspiration and foreign body in airway	81.5 (59.3 to 105.5)	57.1 (42.1 to 77.2)	60.9 (46.0 to 77.7)	49.5 (37.7 to 64.3)
Foreign body in eyes	7.1 (3.7 to 12.2)	6.9 (3.6 to 11.9)	9.0 (5.2 to 14.7)	8.8 (5.0 to 14.2)
Foreign body in other body part	54.5 (34.5 to 81.4)	41.0 (29.3 to 55.2)	50.4 (36.0 to 69.5)	43.5 (32.3 to 56.4)
Environmental heat and cold exposure	48.9 (33.1 to 70.1)	31.9 (21.4 to 46.1)	48.6 (32.9 to 69.8)	34.2 (23.3 to 48.9)
Exposure to forces of nature	0.9 (0.8 to 1.0)	0.4 (0.3 to 0.6)	0.9 (0.8 to 1.0)	0.5 (0.4 to 0.7)
Other unintentional injuries	147.7 (108.8 to 202.4)	82.2 (62.2 to 107.6)	128.6 (93.5 to 183.7)	80.3 (60.9 to 104.9)
Self-harm and interpersonal violence	727.5 (576.4 to 900.7)	677.8 (531.0 to 858.3)	813.3 (626.4 to 1,032.5)	749.0 (588.4 to 966.3)
Self-harm	195.6 (133.3 to 279.3)	156.3 (99.7 to 240.7)	283.5 (194.0 to 410.7)	227.9 (146.9 to 350.5)
Self-harm by firearm	11.8 (6.9 to 20.8)	9.6 (5.9 to 17.0)	16.4 (9.5 to 28.9)	13.1 (8.0 to 23.6)

Self-harm by other specified means	183.7 (126.0 to 266.0)	146.7 (93.7 to 225.7)	267.1 (182.5 to 389.2)	214.7 (138.1 to 330.1)
Interpersonal violence	469.8 (367.1 to 595.9)	394.8 (266.9 to 535.8)	473.0 (353.9 to 615.2)	401.3 (273.8 to 546.3)
Physical violence by firearm	118.2 (86.2 to 155.1)	100.0 (61.2 to 143.9)	115.2 (79.3 to 155.4)	98.5 (59.8 to 143.5)
Physical violence by sharp object	168.3 (115.3 to 230.4)	138.6 (81.6 to 195.3)	182.6 (119.7 to 253.8)	149.8 (90.7 to 211.1)
Sexual violence	17.2 (11.2 to 24.5)	17.4 (11.4 to 24.8)	19.3 (12.7 to 27.6)	19.4 (12.8 to 27.8)
Physical violence by other means	166.1 (127.9 to 214.7)	138.8 (90.0 to 192.4)	155.8 (116.2 to 212.2)	133.7 (87.6 to 186.0)
Conflict and terrorism	55.0 (50.1 to 60.9)	115.2 (101.9 to 132.0)	49.6 (45.2 to 55.0)	106.9 (94.0 to 123.2)
Executions and police conflict	7.1 (6.5 to 7.8)	11.7 (9.8 to 14.1)	7.2 (6.6 to 7.9)	12.9 (10.7 to 15.9)
Total cancers	1,149.9 (870.5 to 1,451.7)	1,172.3 (888.7 to 1,531.1)	1,957.9 (1,482.3 to 2,431.5)	2,262.2 (1,734.5 to 2,887.5)
Total burden related to hepatitis B	487.1 (335.8 to 682.8)	329.6 (215.9 to 491.7)	814.5 (552.4 to 1,155.3)	588.2 (393.1 to 863.2)
Total burden related to hepatitis C	71.3 (45.8 to 106.5)	45.9 (30.2 to 70.5)	113.2 (75.4 to 165.2)	85.2 (57.6 to 125.9)
Total burden related to Non-alcoholic fatty liver disease (NAFLD)	50.2 (30.8 to 78.3)	44.8 (26.6 to 73.4)	100.3 (62.9 to 156.9)	93.3 (56.4 to 148.3)

Table S3 – All age and age-standardised DALYs for all risk factors, Nigeria 1998 and 2019

	All ages		Age-standardized rates	
	1998	2019	1998	2019
All risk factors	57,260.5 (50,475.0 to 63,536.2)	26,509.1 (22,059.8 to 31,690.1)	42,883.6 (37,976.7 to 47,599.0)	25,353.7 (21,544.1 to 29,055.9)
Environmental/occupational risks	32,766.7 (27,255.6 to 38,426.0)	13,430.6 (10,654.1 to 16,628.4)	22,826.0 (19,168.6 to 26,610.2)	11,553.9 (9,522.3 to 13,768.0)
Unsafe water, sanitation, and handwashing	20,340.2 (14,976.0 to 25,465.6)	7,467.7 (5,652.7 to 9,734.0)	13,137.4 (9,386.9 to 16,869.6)	5,663.5 (4,364.1 to 7,218.4)
Unsafe water source	15,497.2 (10,680.6 to 20,266.1)	5,576.7 (4,052.1 to 7,446.5)	10,188.1 (6,818.4 to 13,802.1)	4,239.8 (3,078.7 to 5,501.5)
Unsafe sanitation	11,430.9 (8,137.4 to 14,587.0)	3,674.6 (2,817.4 to 4,835.9)	7,517.6 (5,145.2 to 9,974.1)	2,766.6 (2,123.4 to 3,572.5)
No access to handwashing facility	8,941.7 (6,283.2 to 11,372.5)	3,366.1 (2,326.3 to 4,577.3)	5,644.3 (3,919.2 to 7,270.9)	2,545.0 (1,777.9 to 3,382.5)
Air pollution	13,211.4 (10,442.9 to 17,032.1)	5,885.6 (4,653.6 to 7,415.8)	9,417.5 (7,576.5 to 11,977.3)	5,394.5 (4,405.4 to 6,523.4)
Particulate matter pollution	13,208.2 (10,440.3 to 17,030.4)	5,878.7 (4,648.3 to 7,409.2)	9,409.7 (7,564.4 to 11,970.4)	5,374.8 (4,388.7 to 6,495.1)
Ambient particulate matter pollution	1,625.8 (729.5 to 3,294.7)	1,805.5 (1,018.6 to 2,861.5)	1,283.3 (604.6 to 2,477.3)	1,869.2 (1,125.4 to 2,738.7)
Household air pollution from solid fuels	11,585.6 (8,243.0 to 15,725.0)	4,073.2 (2,842.4 to 5,517.2)	8,129.4 (5,800.5 to 10,867.8)	3,505.6 (2,435.9 to 4,638.5)
Ambient ozone pollution	12.2 (4.8 to 20.8)	14.9 (6.1 to 24.1)	29.7 (11.8 to 50.6)	42.3 (17.7 to 68.0)
Non-optimal temperature	1,867.4 (700.5 to 6,185.3)	780.8 (303.2 to 2,426.9)	1,075.0 (499.9 to 3,137.2)	568.9 (288.6 to 1,560.1)

High temperature	1,694.3 (466.5 to 6,177.1)	705.4 (198.0 to 2,412.8)	957.7 (362.2 to 3,125.9)	500.8 (202.9 to 1,541.4)
Low temperature	182.2 (3.4 to 530.8)	78.9 (2.6 to 232.4)	121.9 (7.0 to 324.5)	70.2 (6.6 to 177.1)
Other environmental risks	135.1 (67.2 to 223.6)	78.9 (39.8 to 124.2)	265.2 (122.2 to 445.2)	176.8 (87.2 to 285.4)
Residential radon	3.2 (0.6 to 6.7)	3.2 (0.7 to 6.7)	7.0 (1.3 to 14.5)	7.8 (1.6 to 16.4)
Lead exposure	132.0 (64.7 to 220.2)	75.7 (37.3 to 120.6)	258.2 (115.3 to 438.0)	169.1 (79.1 to 274.4)
Occupational risks	680.7 (538.5 to 853.8)	441.5 (334.3 to 555.1)	1,005.4 (805.9 to 1,253.4)	688.5 (527.7 to 849.8)
Occupational carcinogens	6.7 (4.1 to 10.6)	5.5 (3.7 to 8.4)	13.3 (8.3 to 21.9)	11.6 (7.6 to 18.3)
Occupational exposure to asbestos	2.1 (0.9 to 5.5)	1.6 (0.8 to 3.9)	4.6 (2.0 to 12.4)	4.3 (2.2 to 10.3)
Occupational exposure to arsenic	0.2 (0.1 to 0.4)	0.2 (0.1 to 0.4)	0.5 (0.2 to 0.8)	0.4 (0.2 to 0.8)
Occupational exposure to benzene	0.5 (0.1 to 0.9)	0.5 (0.1 to 0.9)	0.6 (0.2 to 1.1)	0.6 (0.2 to 1.2)
Occupational exposure to beryllium	0.0 (0.0 to 0.0)	0.0 (0.0 to 0.0)	0.0 (0.0 to 0.0)	0.0 (0.0 to 0.0)
Occupational exposure to cadmium	0.0 (0.0 to 0.0)	0.0 (0.0 to 0.0)	0.0 (0.0 to 0.1)	0.0 (0.0 to 0.1)
Occupational exposure to chromium	0.0 (0.0 to 0.1)	0.0 (0.0 to 0.1)	0.1 (0.1 to 0.1)	0.1 (0.1 to 0.1)
Occupational exposure to diesel engine exhaust	0.7 (0.5 to 1.1)	0.7 (0.5 to 1.0)	1.4 (1.0 to 2.1)	1.4 (1.0 to 2.0)
Occupational exposure to formaldehyde	0.2 (0.2 to 0.3)	0.2 (0.2 to 0.3)	0.3 (0.2 to 0.5)	0.3 (0.2 to 0.4)
Occupational exposure to nickel	0.3 (0.1 to 0.6)	0.2 (0.0 to 0.5)	0.6 (0.1 to 1.3)	0.5 (0.1 to 1.1)
Occupational exposure to polycyclic aromatic hydrocarbons	0.2 (0.1 to 0.3)	0.2 (0.1 to 0.3)	0.4 (0.2 to 0.5)	0.4 (0.3 to 0.5)

Occupational exposure to silica	1.9 (0.8 to 3.4)	1.4 (0.6 to 2.4)	3.8 (1.7 to 6.7)	2.9 (1.3 to 5.0)
Occupational exposure to sulfuric acid	0.6 (0.2 to 1.1)	0.3 (0.1 to 0.7)	1.1 (0.4 to 2.2)	0.6 (0.2 to 1.3)
Occupational exposure to trichloroethylene	0.0 (0.0 to 0.0)	0.0 (0.0 to 0.0)	0.0 (0.0 to 0.0)	0.0 (0.0 to 0.0)
Occupational asthmagens	38.1 (28.6 to 49.0)	22.1 (17.0 to 28.4)	66.0 (48.2 to 85.3)	38.3 (29.4 to 49.3)
Occupational particulate matter, gases, and fumes	43.4 (33.8 to 54.9)	32.1 (25.2 to 39.7)	101.6 (78.7 to 129.0)	86.0 (67.2 to 107.1)
Occupational noise	80.0 (52.8 to 112.1)	70.7 (47.4 to 99.5)	123.3 (83.8 to 171.8)	113.3 (78.2 to 158.5)
Occupational injuries	281.0 (198.0 to 390.3)	124.7 (84.3 to 179.0)	344.1 (240.8 to 481.3)	153.4 (103.6 to 221.0)
Occupational ergonomic factors	231.6 (158.8 to 315.1)	186.4 (127.3 to 256.2)	357.1 (246.5 to 484.8)	285.9 (196.5 to 393.4)
Behavioral risks	48,410.8 (42,619.9 to 53,945.9)	21,752.3 (17,822.4 to 26,650.6)	31,069.7 (27,547.5 to 34,722.4)	17,616.7 (14,789.6 to 20,906.9)
Child and maternal malnutrition	43,011.4 (37,315.4 to 48,447.9)	18,171.9 (14,542.9 to 22,858.0)	22,312.5 (19,303.0 to 25,119.9)	11,360.7 (9,136.6 to 14,260.3)
Suboptimal breastfeeding	4,215.7 (3,054.1 to 5,485.7)	1,573.7 (1,084.1 to 2,143.2)	2,029.7 (1,475.6 to 2,635.4)	961.9 (662.5 to 1,310.4)
Non-exclusive breastfeeding	3,995.8 (2,829.5 to 5,209.0)	1,526.7 (1,040.8 to 2,101.0)	1,908.9 (1,351.3 to 2,488.4)	932.1 (635.2 to 1,282.6)
Discontinued breastfeeding	258.3 (76.9 to 472.9)	56.7 (19.0 to 109.3)	139.3 (41.3 to 257.6)	35.7 (12.0 to 68.9)
Child growth failure	27,258.2 (22,450.8 to 31,790.1)	8,466.0 (6,290.5 to 11,241.2)	14,660.3 (12,016.9 to 17,194.5)	5,329.7 (3,964.2 to 7,076.6)
Child underweight	8,765.1 (6,109.4 to 12,212.5)	1,941.2 (1,388.4 to 2,754.0)	4,828.1 (3,350.2 to 6,764.8)	1,242.1 (894.0 to 1,751.0)

Child wasting	23,268.2 (18,222.2 to 28,830.6)	7,871.2 (5,801.8 to 10,556.1)	12,440.3 (9,698.3 to 15,527.2)	4,952.6 (3,648.6 to 6,641.7)
Child stunting	7,111.5 (3,947.7 to 11,379.5)	1,752.2 (827.5 to 3,071.3)	3,918.4 (2,190.2 to 6,257.9)	1,108.0 (523.3 to 1,936.8)
Low birth weight and short gestation	13,867.9 (11,627.9 to 16,181.2)	8,503.1 (6,845.2 to 10,631.6)	6,380.1 (5,351.4 to 7,446.0)	5,092.5 (4,100.7 to 6,363.1)
Short gestation	11,046.4 (9,263.5 to 13,003.1)	7,069.4 (5,694.1 to 8,880.1)	5,083.0 (4,262.2 to 5,979.7)	4,236.8 (3,416.7 to 5,319.5)
Low birth weight	12,685.6 (10,667.8 to 14,841.7)	7,838.2 (6,302.3 to 9,819.4)	5,838.0 (4,909.5 to 6,829.1)	4,696.2 (3,777.5 to 5,878.9)
Iron deficiency	1,029.9 (693.1 to 1,440.9)	979.0 (654.4 to 1,404.0)	839.2 (559.1 to 1,178.5)	799.5 (534.5 to 1,126.8)
Vitamin A deficiency	1,571.7 (205.1 to 3,281.8)	121.5 (36.9 to 232.6)	871.2 (116.2 to 1,813.9)	78.5 (25.2 to 149.1)
Zinc deficiency	121.5 (15.2 to 314.1)	18.0 (0.3 to 57.6)	70.1 (8.7 to 181.3)	11.6 (0.2 to 37.1)
Tobacco	900.5 (632.2 to 1,190.0)	488.3 (362.0 to 632.5)	1,295.1 (1,002.0 to 1,625.6)	851.1 (666.6 to 1,085.1)
Smoking	437.7 (332.5 to 562.7)	258.7 (196.6 to 337.2)	894.2 (687.7 to 1,137.3)	578.8 (443.3 to 748.1)
Chewing tobacco	0.2 (0.1 to 0.4)	0.2 (0.1 to 0.4)	0.5 (0.3 to 0.8)	0.6 (0.4 to 0.9)
Secondhand smoke	471.2 (252.5 to 732.0)	235.2 (143.3 to 341.2)	418.5 (257.9 to 596.9)	285.4 (192.4 to 387.8)
Alcohol use	1,154.6 (807.8 to 1,569.1)	790.2 (551.4 to 1,103.5)	2,030.3 (1,413.0 to 2,772.7)	1,434.4 (999.2 to 2,015.9)
Drug use	433.1 (283.7 to 645.1)	332.9 (247.4 to 444.0)	597.7 (391.5 to 893.7)	474.6 (355.4 to 627.3)
Dietary risks	944.6 (644.7 to 1,406.2)	655.1 (460.9 to 930.5)	2,099.2 (1,451.6 to 3,110.0)	1,597.4 (1,132.0

				to 2,256.4)
Diet low in fruits	188.4 (116.3 to 296.8)	127.4 (82.7 to 185.8)	402.4 (251.1 to 627.7)	293.2 (190.5 to 420.2)
Diet low in vegetables	143.4 (75.0 to 234.6)	78.8 (42.3 to 122.1)	310.2 (167.7 to 495.4)	186.1 (103.3 to 283.6)
Diet low in legumes	138.6 (18.6 to 271.4)	88.1 (11.6 to 162.0)	306.7 (41.8 to 596.0)	215.6 (29.7 to 399.5)
Diet low in whole grains	229.7 (107.5 to 352.9)	163.8 (85.1 to 235.6)	506.9 (235.5 to 770.1)	397.1 (208.0 to 562.1)
Diet low in nuts and seeds	10.9 (4.3 to 23.9)	3.2 (2.0 to 5.5)	24.8 (10.0 to 53.4)	8.7 (5.5 to 15.0)
Diet low in milk	12.0 (7.4 to 17.6)	14.3 (8.9 to 21.0)	26.5 (16.2 to 38.4)	34.8 (22.0 to 50.1)
Diet high in red meat	59.6 (24.1 to 102.4)	51.7 (23.0 to 83.1)	125.1 (51.4 to 211.4)	115.8 (52.3 to 184.4)
Diet high in processed meat	47.3 (23.2 to 90.9)	43.9 (23.2 to 72.6)	101.5 (50.4 to 192.6)	102.5 (55.0 to 168.7)
Diet high in sugar-sweetened beverages	54.1 (37.8 to 76.8)	35.1 (25.2 to 47.2)	126.3 (89.5 to 175.8)	91.3 (66.2 to 122.0)
Diet low in fiber	26.9 (13.3 to 47.7)	10.2 (6.1 to 17.0)	58.7 (29.6 to 101.7)	24.7 (15.2 to 39.6)
Diet low in calcium	15.2 (10.5 to 21.3)	16.5 (11.6 to 22.9)	33.6 (23.6 to 46.4)	40.3 (28.9 to 55.0)
Diet low in seafood omega-3 fatty acids	44.9 (14.9 to 76.0)	30.8 (10.4 to 47.2)	99.3 (33.5 to 166.0)	75.0 (25.7 to 113.5)
Diet low in polyunsaturated fatty acids	65.8 (9.6 to 141.4)	45.4 (6.0 to 94.1)	144.5 (21.5 to 307.5)	109.6 (14.7 to 226.4)
Diet high in trans fatty acids	54.4 (8.1 to 94.8)	35.2 (5.2 to 59.4)	121.7 (18.6 to 210.6)	87.1 (13.8 to 145.5)
Diet high in sodium	132.9 (6.4 to 548.9)	94.1 (4.5 to 360.6)	297.6 (14.5 to 1,225.4)	231.8 (11.1 to 886.3)

Intimate partner violence	265.7 (134.3 to 453.8)	186.3 (105.0 to 295.7)	336.1 (174.8 to 559.8)	259.6 (151.1 to 403.1)
Childhood sexual abuse and bullying	99.9 (52.5 to 162.9)	94.6 (49.3 to 154.6)	117.8 (62.3 to 189.2)	109.8 (58.7 to 175.7)
Childhood sexual abuse	61.8 (31.2 to 101.6)	52.8 (26.8 to 85.4)	85.6 (44.1 to 141.9)	75.7 (39.2 to 122.9)
Bullying victimization	39.9 (10.9 to 88.8)	43.6 (12.4 to 94.5)	33.8 (8.6 to 77.7)	35.7 (9.2 to 80.8)
Unsafe sex	2,525.2 (1,666.7 to 3,841.2)	1,569.0 (1,215.8 to 2,005.1)	3,325.6 (2,246.5 to 4,892.0)	2,213.4 (1,784.2 to 2,733.0)
Low physical activity	65.9 (29.3 to 142.2)	53.9 (25.2 to 108.3)	166.9 (75.6 to 352.6)	154.1 (72.9 to 296.6)
Metabolic risks	2,913.6 (2,290.9 to 3,712.7)	2,388.4 (1,895.8 to 2,935.7)	6,204.2 (4,938.1 to 7,909.7)	5,571.8 (4,505.4 to 6,738.7)
High fasting plasma glucose	821.3 (654.6 to 1,016.8)	714.5 (551.1 to 893.2)	1,872.0 (1,494.8 to 2,342.4)	1,826.3 (1,403.0 to 2,278.4)
High LDL cholesterol	493.6 (320.2 to 742.6)	354.3 (247.6 to 484.7)	1,073.7 (703.3 to 1,612.2)	831.9 (562.1 to 1,137.8)
High systolic blood pressure	1,551.3 (1,160.2 to 2,134.3)	1,339.6 (1,043.2 to 1,676.9)	3,522.3 (2,656.7 to 4,764.8)	3,285.4 (2,607.5 to 4,029.8)
High body-mass index	668.5 (329.2 to 1,133.6)	778.9 (468.1 to 1,138.5)	1,340.4 (655.4 to 2,289.6)	1,655.8 (971.3 to 2,454.6)
Low bone mineral density	69.2 (55.8 to 84.7)	60.2 (48.5 to 73.6)	166.1 (135.3 to 201.0)	161.3 (130.2 to 195.6)
Kidney dysfunction	586.1 (468.1 to 724.2)	472.5 (374.5 to 581.3)	1,012.2 (815.6 to 1,246.4)	921.7 (735.6 to 1,122.7)
